# The genome assembly and annotation of yellowhorn (*Xanthoceras sorbifolium* Bunge)

**DOI:** 10.1093/gigascience/giz071

**Published:** 2019-06-26

**Authors:** Qiang Liang, Huayang Li, Shouke Li, Fuling Yuan, Jingfeng Sun, Qicheng Duan, Qingyun Li, Rui Zhang, Ya Lin Sang, Nian Wang, Xiangwen Hou, Ke Qiang Yang, Jian Ning Liu, Long Yang

**Affiliations:** 1 College of Forestry, Shandong Agricultural University, Daizong Road No.61,Tai'an 271018, China; 2 College of Plant Protection, Shandong Agricultural University, Daizong Road No.61, Tai'an 271018, China; 3 Worth Agricultural Development Co. Ltd.,Taishanxi Road No. 17, Anqiu city, Weifang 262100, China; 4 KeGene Science & Technology Co. Ltd., Nantianmen Middle Road, Tai'an 271018, China

**Keywords:** yellowhorn (*Xanthoceras sorbifolium* Bunge), PacBio sequencing, BioNano Genomics, 10X Genomics Chromium, high-throughput chromosome conformation capture, Illumina paired-end sequencing

## Abstract

**Background:**

Yellowhorn (*Xanthoceras sorbifolium* Bunge), a deciduous shrub or small tree native to north China, is of great economic value. Seeds of yellowhorn are rich in oil containing unsaturated long-chain fatty acids that have been used for producing edible oil and nervonic acid capsules. However, the lack of a high-quality genome sequence hampers the understanding of its evolution and gene functions.

**Findings:**

In this study, a whole genome of yellowhorn was sequenced and assembled by integration of Illumina sequencing, Pacific Biosciences single-molecule real-time sequencing, 10X Genomics linked reads, Bionano optical maps, and Hi-C. The yellowhorn genome assembly was 439.97 Mb, which comprised 15 pseudo-chromosomes covering 95.42% (419.84 Mb) of the assembled genome. The repetitive fractions accounted for 56.39% of the yellowhorn genome. The genome contained 21,059 protein-coding genes. Of them, 18,503 (87.86%) genes were found to be functionally annotated with ≥1 "annotation" term by searching against other databases. Transcriptomic analysis showed that 341, 135, 125, 113, and 100 genes were specifically expressed in hermaphrodite flower, staminate flower, young fruit, leaf, and shoot, respectively. Phylogenetic analysis suggested that yellowhorn and *Dimocarpus longan* diverged from their most recent common ancestor ∼46 million years ago.

**Conclusions:**

The availability and subsequent annotation of the yellowhorn genome, as well as the identification of tissue-specific functional genes, provides a valuable reference for plant comparative genomics, evolutionary studies, and molecular design breeding.

## Data Description

### Introduction

Yellowhorn (*Xanthoceras sorbifolium* Bunge, NCBI:txid99658), the single species of genus *Xanthocera* (Sapindaceae), is a deciduous shrub or small tree, naturally occurring on hills and slopes in Northern China [1–3]. Yellowhorn is resistant to cold, drought, and salinity [4, 5] and is of important ecological, economic, and pharmacological value [6]. Yellowhorn is an andromonoecious plant that has both hermaphrodite and staminate flowers, and produces capsular fruits from hermaphrodite with seeds rich in oil (50–68% of kernel), which contains 85–93% unsaturated fatty acids, being especially remarkable in nervonic acid content [5, 7]. The stems and fruits of yellowhorn have been used in folk medicine in Inner Mongolia for the treatment of rheumatism, gout, and enuresis in children [8]. Moreover, different yellowhorn tissues contain multiple bioactive compounds, including triterpenoid saponins, barringenol-like triterpenoids, which have been found to possess antitumor and anti-inflammatory activities, as well as potentiality against Alzheimer disease [8–12].

The Sapindaceae family (also known as the soapberry family) comprises 142 genera and 1,900 species including important tropical fruits and woody oil-bearing plants, such as *Dimocarpus longan, Litchi chinensis, Nephelium lappaceum, Sapindus mukorossi*, and yellowhorn [13, 14]. The genome of *D. longan* has been sequenced and assembled recently [15]. The chloroplast genome of yellowhorn has been assembled and characterized using Illumina pair-end sequencing data [16]. Genes regulating oil accumulation and fertilized ovule development have been identified in yellowhorn [17, 18]. Despite the increasing availability of genetic resources with research and economic value, a fully annotated genome is currently unavailable for yellowhorn.

In this study, a high-quality draft genome of yellowhorn was sequenced and assembled by integration of Illumina sequencing, Pacific Biosciences (PacBio) single-molecule real-time sequencing, 10X Genomics linked reads, Bionano optical maps, and high-throughput chromosome conformation capture (Hi-C). Functional annotation for protein coding genes was performed. Tissue-specific genes were identified and analyzed through transcriptomic approaches. Our study will facilitate comparative genomics, gene functional studies, and molecular assisted breeding in the near future.

### Methods

#### Plant material

**Figure 1:**
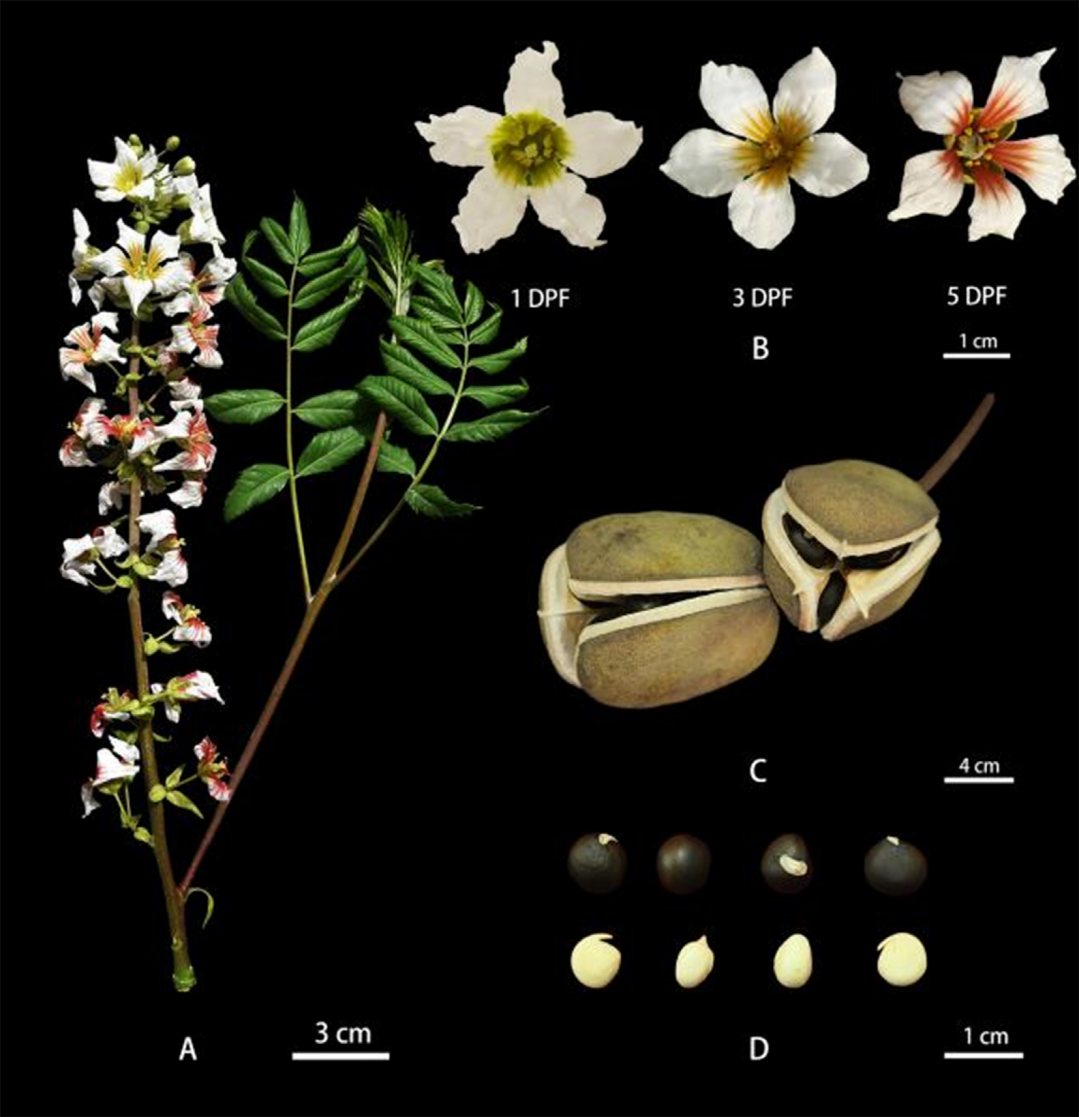
Morphological characteristic of yellowhorn superior “WF18”. (A) Raceme and shoot. (B) Hermaphrodite flower at 1, 3, and 5 DPF (days post flower). (C) Capsular fruits. (D) Seeds and kernel.

The yellowhorn superior tree (voucher No. “WF18”) with high seed yield and high oil content in the kernel was conserved at the Forestry Experimental Station of Shandong Agricultural University, Tai'an, Shandong, China (36.171 E, 117.149 N), and was used for genome sequencing (Fig. 1). Genomic DNA was extracted from freshly flushed leaf of the WF18 tree using NucleoSpin Plant II (MachereyeNagel, Düren, Germany) and Bionano Prep Plant Tissue DNA Isolation Protocol (Bionano Genomics, San Diego, CA, USA). The quality and quantity of DNA was assessed using 0.8% agarose gels and Qubit fluorimeter (Invitrogen, Carlsbad, CA, USA). Total RNA was isolated using the GeneJET Plant RNA Purification Mini Kit (Thermo Fisher Scientific, Waltham, Massachusetts, USA) from 5 tissues of the WF18 tree including hermaphrodite flower, staminate flower, young fruit, leaf, and shoot, and quantified by NanoDrop ND-2000 (Thermo Fisher Scientific, Waltham, MA, USA). RNA integrity was assessed using the Agilent Bioanalyzer 2100 (Agilent Technologies, Santa Clara, California, USA). The sample with integrity number >8 was used for library construction. For Hi-C library construction, ∼5 g freshly flushed leaves were cross-linked with 1% formaldehyde for 10 minutes at room temperature, which was then quenched with a final concentration of 0.125 mol/L glycine. The cross-linked leaf tissues were used for isolating intact nuclei according to the previously reported method [19].

#### Genomic DNA sequencing

For Illumina sequencing, 2 libraries with insert sizes of 280 and 450 bp were constructed using NEBNext Ultra II DNA Library Prep Kit (New England Biolabs, Ipswich, MA, USA). The libraries were then sequenced on an Illumina HiSeq X Ten System using a PE-150 module, and 172 Gb raw data were generated. The quality of all raw reads was assessed using FASTQC v. 0.11.7 with default parameter settings. The adapters and low-quality bases were trimmed using Trimmomatic v. 0.38 (Trimmomatic, RRID:SCR_011848) with default parameter settings [20]. Approximately 164.79 Gb (∼375× of assembled genome size 439.97 Mb) clean reads were obtained for pre−*de novo* genome assembly (Table 1).

**Table 1:** Statistics of Illumina, 10X Genomics, and Hi-C sequencing data

Platform	Library type	Read length (bp)	No. of raw reads (Mb)	Reads retained after trimming (Mb)	Total valid base (Gb)
Illumina	280 bp size	150	451.34	439.84	65.98
	450 bp size	150	696.88	658.72	98.81
10X Genomics	350 bp size	150	457.40	457.40	63.35
Hi-C	600 bp size	150	932.76	891.72	133.76

For PacBio long-read sequencing, a 20-kb single-molecule real-time DNA sequencing library was constructed according to the manufacturer's protocol (Pacific Biosciences, Menlo Park, CA, USA). The libraries were used for sequencing on the PacBio Sequel platform and yielded >70.62 Gb (∼160× the assembled genome size) subreads.

The library of 10X Genomics was prepared using the Chromium Gel Bead and Library Kit (10X Genomics, Pleasanton, CA, USA) and the Chromium instrument (10X Genomics) following the manufacturer's protocol. The barcoded library was sequenced on an Illumina NovaSeq 6000 system. The BCL files were demultiplexed and converted to fastq files using Supernova mkfastq (v. 2.0.0) with default parameter settings. After the first 23 bases were trimmed from the beginning of read 1 of each pair (the 16-base 10X barcode plus 7 additional bases) by Supernova (v. 2.0.0) with default parameter settings, ∼457.40 Mb reads with a mean length of 138.5 bp were generated. The fraction of Q30 in read 2 was 83.42% (Table 1).

Two Bionano optical maps were analyzed with Saphyr's streamlined workflow (BioNano Genomics, San Diego, CA, USA). High molecular weight DNA was treated with Nt.BspQI and Nt.BssSI nicking endonucleases (New England Biolabs), respectively. Fluorescent nucleotides were incorporated by nick translation (Bionano Prep Labeling—NLRS Protocol). After the nicks were repaired, the DNA sample was electrophoresed into massively parallel nanochannel imaging. More than 325 Gb (from Nt.BspQI) and 266 Gb (from Nt.BssSI) image data were collected with a minimum molecule length of 150 kb, respectively (Table 1).

A Hi-C library was generated using DpnII restriction enzyme following *in situ* ligation protocols [21]. The DpnII-digested chromatin was end-labeled with biotin-14-dATP (Thermo Fisher Scientific, Waltham, MA, USA) and used for *in situ* DNA ligation. The DNA sample was extracted and purified, and then sheared using Covaris S2 (Covaris, Woburn, MA, USA). After A-tailing, pull-down, and adapter ligation, the DNA library was sequenced on an Illumina HiSeq X Ten System using a PE-150 module. More than 133.76 Gb (∼304× of assembled genome size) clean data were generated after trimming low-quality reads and removing adapters by Trimmomatic v. 0.38 with default parameter settings (Table 1).

#### Transcriptome sequencing

The RNA library was constructed using the TruSeq RNA Sample Preparation Kit v2 (Illumina, San Diego, CA, USA) and the deoxyuridine triphosphate (dUTP) method [22]. The 5 RNA libraries with insert size ∼350 bp were sequenced on an Illumina HiSeq 4000 System using a PE-150 module. The quality of all raw reads was assessed using FASTQC v. 0.11.7 with default parameter settings. The adapters and low-quality bases were trimmed using Trimmomatic v. 0.38 with default parameter settings [20]. As a result, >44.42 Gb clean data were generated (Table S1). The quality-checked sequencing reads were aligned to assembled genome using HISAT2 v.2.1.0 in strand-specific mode [23, 24], and the result showed that the genome mapping rate was 75.68%. The quality-checked reads were also aligned to assembled genome by Tophat v. 2.1.2 in strand-specific mode with a minimum intron length of 20 bp and a maximum intron length of 20 kb [25]. The transcripts were assembled using StringTie v. 1.3.4d with default parameters [23, 26]. The abundance of gene expression was estimated using the “scaledTPM” method in the txImport v. 1.8.0 package with default parameter settings [27]. Gene Ontology (GO) functional enrichment analysis was performed based on the comparison with all protein-coding genes assigned to the GO terms using Fisher's exact test implemented in topGO package v. 2.3.4 with default parameters. In addition, the quality-checked sequencing reads were also *de novo* assembled with genome-guided or *de novo* model using Trinity v. 2.5.1 (Trinity, RRID:SCR_013048) [28] in strand-specific mode with min_kmer_cov 2 and min_glue 5. All assembled transcritps were further incorporated to train *ab initio* predictors for gene prediction (see below, “Genome annotation”).

#### Karyotype analysis and genome size estimation

Flower buds (2–2.5 mm) of the WF18 tree were collected between 8:00 and 11:00 a.m. of early April days in 2017 and fixed directly in Carnoy's solution (ethanol: acetic acid, 3:1) at 4°C for 24 h. Flower buds were hydrolyzed in 1 mol/L HCl at 60°C for 5 min, and then washed in distilled water for 3 min. Anthers were obtained as previously described [29]. At least 5 dispersive meiosis metaphase plates for each karyotype were observed using an Eclipse Ni-U photomicroscope (Nikon, Tokyo, Japan) equipped with a DS-Ri2 high-sensitivity camera (Nikon) with a Y-TV55 TV adapter (Nikon) on the trinocular tube. The images were captured and the chromosome length (CL), long arm length (LL), and short arm length (SL) of each chromosome were measured by imaging software NIS-Elements D v5.11.00 (Nikon). Then karyotypes were organized with Photoshop .v CS2 (Adobe, San Jose, CA, USA) and an ideogram was generated on the basis of the haploid set length (HSL), the relative length of the short arm (S = SL/HSL  ×  100%), the relative length of the long arm (L =  LL/HSL  ×  100%), and the total chromosome (TL = S + L) using Excel 2010 (package of Microsoft Office 2010). The chromosomes were classified according to the specifications [30], based on the chromosome arm ratio *r* between the long and short arms (*r* = L/S): m = median (*r* = 1–1.7), sm = submedian (*r* = 1.7–3), st = subterminal (*r* = 3–7), and t = terminal (*r* > 7).

The yellowhorn genome size was estimated by flow cytometry [31]. Fresh leaves were chopped with a razor blade in a Petri dish containing 1 mL of Otto I buffer (0.1 mol/L citric acid monohydrate, 0.5% (v/v) Tween­20, pH 2­.3) and then filtered through a 50-µm nylon mesh and centrifuged at 100 g for 8 min. The pellet was resuspended in 200 μL buffer of a 1:2 mixture of Otto I and Otto II (0.4 mol/L Na_2_HPO_4_·12H_2_O, pH 8.9) and stained with 50 μg/mL propidium iodide including 50 μg/mL RNase. Four replicates were analyzed. For each replicate, >5,000 nuclei were measured using an Elite flow cytometer (Becton Dickinson, San Jose, CA, USA). The coefficient of variation of the histogram peak was <5%. The species of *"Solanum pimpinellifolium*" LA1589 with draft genome size of 739 Mb was used as external reference standard [32]. The yellowhorn genome size was estimated on the basis of the *k*-mer frequency spectrum. The sequence reads from Illumina insert size of 280 and 450 bp libraries were prepared to construct the *k*-mer library using KMC v. 3.1.0 [33] with *k*-mer length ranging from 17 to 200 and parameter settings as follows: “-m50 -cs12000”. GenomeScope v. 1.0 [34] was used to estimate genome size and evaluate genome heterozygosity based on the *k*-mer frequency spectrum calculation from KMC.

#### Genome assembly by PacBio long reads

The genomic contigs were assembled on the basis of PacBio subreads using Falcon v. 0.7.0 (Falcon, RRID:SCR_016089) [35]. First, raw subreads were aligned to each other for error correction using Daligner v. 1.0 (Daligner, RRID:SCR_016066) [36] with the following parameter settings: “sge_option_da = ‐pe smp 4 ‐q bigmem; sge_option_la = ‐pe smp 20 ‐q bigmem; pa_DBsplit_option = ‐a ‐x500 ‐s100; pa_HPCdaligner_option = ‐v ‐B128 ‐t16 ‐e0.8 ‐M24 ‐l3200 ‐k18 ‐h480 ‐w8 ‐s100; pa_concurrent_jobs = 8”. Then overlapped error-corrected reads were processed to generate consensus reads by a binary executable LA4Falcon to script “fc_consensus.py” with the following parameter settings: “falcon_sense_option = ‐ output_multi ‐min_cov_aln 4 ‐min_idt 0.70 ‐min_cov 4 ‐max_n_read 200 ‐n_core 8; cns_concurrent_jobs = 8”. Furthermore, length_cutoff values of 2,000, 3,000, and 5,000 were chosen, respectively, to filter raw reads in the first round for error correction. In the second round, length_cutoff_pr values of 5,000, 8,000, and 10,000 were chosen for assembling the overlapping step, respectively, to obtain consensus overlapping reads the with following parameter settings: “sge_option_pda = ‐pe smp 6 ‐q bigmem; sge_option_pla = ‐pe smp 16 ‐q bigmem; ovlp_concurrent_jobs = 8; ovlp_DBsplit_option = ‐s100; ovlp_HPCdaligner_option = ‐v ‐B128 ‐M24 ‐k24 ‐h1024 ‐e.9 ‐l2500 ‐s100”. The consensus overlapping reads were filtered with the following parameters: “overlap_filtering_setting = ‐max_diff 80 ‐max_cov 80 ‐min_cov 2 ‐n_core 12” and used to construct string graphs by script “fc_ovlp_to_graph.py” using the default parameters.

The draft genomic contigs were polished using PacBio long reads and Illumina paired-end reads. First, the PacBio long reads were mapped to the genomic contigs using Pbalign v. 0.3.1 with default parameter settings. The self-polished consensus contigs were generated using the Arrow algorithm of the variantCaller tool within GenomicConsensus package v. 2.3.2 with default parameters. Second, the Illumina paired-end libraries of 280 and 450 bp were aligned to the self-polished consensus contigs with BWA-MEM algorithm with default parameter settings in the BWA package v. 0.7.17 (BWA, RRID:SCR_010910) [37] and final polished contigs were obtained using Pilon v. 1.22 (Pilon, RRID:SCR_014731) with default parameters [38].

#### Pseudo-chromosome construction using 10X Genomics, BioNano optical maps, and Hi-C

The polished contigs were first scaffolded with the 10X Genomics linked reads by fragScaff v. 140 324.1 [39]. By mapping the linked reads to polished contigs with the BWA MEM algorithm with the default parameter settings, the alignment of each library was sorted and merged into a bamParse file using samtools v. 1.3.1 (SAMTOOLS, RRID:SCR_002105) with default parameters and filtered with parameter “min N spacer size 3000, contig end node size 5000 and max contig end node size 10000”.

The 10X Genomics scaffold was *in silico* digested with the nicking enzymes Nt.BspQI and Nt.BssSI, respectively, using perl script “fa2cmap_multi_color.pl” with default parameters in the Bionano Solve v. 3.1 (BioNano Genomics). Scaffold genome of *in silico* maps and each BioNano Genomics map were processed using the hybrid scaffold algorithm with default parameter settings in Bionano Solve v. 3.1 to directly generate a hybrid scaffold.

The gaps distributed in hybrid super-scaffolds were filled with PacBio consensus long reads by PBJelly v. 15.2.20 (PBJelly, RRID:SCR_012091) [40] with the following parameter settings: “‐minMatch 8 ‐minPctIdentity 70 ‐bestn 1 ‐nCandidates 20 ‐maxScore -500 ‐nproc 20 ‐noSplitSubreads”. Subsequently, the gaps were further filled with Illumina insert size of 280 and 450 bp library paired-end reads by GMcloser v. 1.6.2 [41] with parameter settings “‐l 150 ‐i 280 ‐c ‐n 20” for insert size of 280 bp library and “‐l 150 ‐i 450 ‐c ‐n 20” for insert size of 450 bp library.

The gap-closed hybrid scaffolds were aligned to generate duplicate free Hi-C contacts based on *in situ* Hi-C data using Juicer pipeline v. 1.6.2 [42]. The gap-closed hybrid scaffolds were first *in silico* digested with the restriction enzyme DpnII using the python script “generate_site_positions.py” with default parameters in the Juicer pipeline. The cleaned Hi-C reads were then mapped to the hybrid scaffolds and processed to generate Hi-C contacts by Juicer pipeline with the following parameter settings: “‐s DpnII ‐t 20”. The duplicate free Hi-C contacts file (merged_nodups.txt) was used for *de novo* assembly by the 3D-DNA pipeline v. 180 419 [43] with the default parameters. For the pre-processing stage, a range of iterative steps and algorithms were performed to eliminate misjoins in the input hybrid scaffolds. The scaffolding algorithm was first applied to order and orient the scaffolds. With 2 iterations of the misjoin correction algorithm, the revised scaffolds were used as input for the scaffolding algorithm to output “megascaffold” that concatenates all the pseudo-chromosomes. The megascaffold was imported to Juicebox Assembly Tools (JBAT) v. 1.8.8 [44] for manual review and refinement.

#### Genome annotation

For repetitive element detection, the RepBase plant repeat database (v. 23.06) and a *de novo* repeat library were used to annotate repeat sequences in the yellowhorn genome assembly. *De novo* repetitive element annotation was performed using RepeatModeler v. 1.0.11 (RepeatModeler, RRID:SCR_015027) with default parameter settings. All Modelerunknown repeat family's sequences were searched against the UniProt plant protein database (accessed 31 January 2018) using BLASTX with E value setting of 1 e–10 in BLAST v. 2.7.1+. The blastx result was then used to exclude gene fragments from *de novo* predict repeats using ProtExcluder v. 1.2 with default parameters. Finally, the *de novo* reliable predict repeats in the genome assembly and repetitive elements in RepBase were annotated by running RepeatMasker v. 4.07 (RepeatMasker, RRID:SCR_012954) with default parameter settings.

Gene prediction was performed by combining the evidence obtained from *ab initio* predictors based on hidden Markov Model, spliced transcript evidence from the transcript assembly by Trinity, and protein homology evidence from the proteins of related plants aligned against the yellowhorn genome assembly. For *ab initio* gene prediction, 3 predictors, namely, Augustus v. 3.2.2 (Augustus: Gene Prediction, RRID:SCR_008417) [45], SNAP (accessed 28 July 2006) gene finder [46], and GeneMark-ES/ET v. 4.3.5 [47], were performed on repeat-masked yellowhorn genome. First, spliced transcripts generated from Trinity following a *de novo* and genome-guided model were aligned against the yellowhorn genome with PASA v. 2.3.3 [48] following default parameter settings to get reliable open reading frames (ORFs) used for training *ab initio* predictors. The Augustus *ab initio* model was generated by running the Augustus program with 5 rounds of training and 8-fold cross validation based on the best ORFs obtained from PASA. Final gene models were predicted using the *ab initio* trained model with the intron hints from RNA sequencing junctions and Trinity assembled transcripts. SNAP *ab initio* models were obtained using the same gene sets as Augustus with 1 round. The gene models were finally predicted with the trained model following default parameters. GeneMark-ES/ET gene models were predicted with intron hints under unsupervised training following default parameter settings. To predict genes based on similarity, protein sequences of *Citrus sinensis, D. longan, Theobroma cacao, Olea europaea, Anacardium occidental, Vitis vinifera, Glycine max, Populus tremula, Oryza sativa*, and *Arabidopsis thaliana* were spliced-mapped to the repeat-masked yellowhorn genome assembly using Exonerate v. 2.2.0 [49] with protein2genome model at 90% identity. Gene models from *ab initio* and homology predictions were combined to get a single high-confidence gene model by EVidenceModeler (EVM) v. 2.4.0 following the developer’s suggestions [50]. Weights were set according to the confidence of the PASA Trinity set, weight 10; Augustus gene set, weight 6; Exonerate protein homology set, weight 2; SNAP gene model set, weight 2; and GeneMark-ES/ET gene set, weight 1.

The functions of predicted protein coding genes were annotated by searching against the NCBI NR database (accessed 31 January 2018) and UniProt (accessed 31 January 2018) using BLASTX with E value setting of 1 e–5, coverage ≥ 50%, and identity ≥ 30% in BLAST v. 2.7.1+. Pfam domain annotation was performed by alignment with the Pfam database (Pfam 28) (accessed 20 May 2015) using HMMER v. 3.1b2 (Hmmer, RRID:SCR_005305) with default parameters [51]. GO terms of the predicted protein coding genes were assigned using Blast2GO v. 4.1.9 with default parameters [52]. KEGG annotation was assigned by searching against the KEGG GENES database in the KEGG Automatic Annotation Server (KAAS) web server with bi-directional best hit [53]. CAZy annotation was implemented by alignment with the CAZy database (accessed 20 July 2017) using dbSCAN v. 6.0 [54] following default parameter settings.

#### Comparative phylogenomics

The protein sequences of yellowhorn, together with *C. sinensis, D. longan, T. cacao, O. europaea, A. occidentale, V. vinifera, G. max, P. tremula, O. sativa*, and *A. thaliana* containing only 1 transcript per gene, were retrieved and filtered by removing redundancy of alternative spliced and low-quality proteins using the program orthomclFilterFasta in OrthoMCL v. 2.0.9 (OrthoMCL DB: Ortholog Groups of Protein Sequences, RRID:SCR_007839) [55] with “min_length 30 and max_percent_stop 20”. The produced proteins were also manually checked and the mitochondrial and plastid genes were filtered away by searching against all conserved mitochondrial and plastid genes available from GenBank (accessed 10 July 2018) using BLASTP in BLAST v. 2.7.1+ with default parameters. The all-vs-all alignment based on the filtered proteins was performed using BLASTP in BLAST v. 2.7.1+ with the following parameters: “-evalue e-5 ‐seg yes ‐outfmt 6”. The blast collections were used to find pairs of proteins that are potentially orthologs, in-paralogs, or co-orthologs by means of the program orthomclPairs in OrthoMCL using a cut-off of 1 e–5 and 50% match. All of the pairs were further clustered into groups using the program mcl in OrthoMCL with parameters “‐abc ‐I 1.5”.

The protein sequences of 195 single-copy orthologous genes that shared single-copy genes among the plant species were performed to generate multiple sequence alignment using MAFFT v. 7.158b (MAFFT, RRID:SCR_011811) with an accurate option (L-INS-i) [56]. After each alignment merging, GBlocks v. 0.91b [57] with default parameters was used to remove poorly aligned positions, divergent regions, and selected conserved blocks. Phylogeny was constructed using RAxML v. 8.1.24 [58] with the evolutionary model GTR+GAMMA. A total of 1,000 rapid bootstrap inferences were performed. Divergence time of species was estimated using MCMCTree in the PAML 4.9h (PAML, RRID:SCR_014932) package [59] with correlated rates clock and JC69 model settings following 5 MCMCTree runs. The Markov chain Monte Carlo analysis was run on 20,000 generations with a burn-in of 2,000 iterations. Divergence time estimates were extrapolated using secondary calibration points from the TimeTree database [60] for *A. thaliana–T. cacao* split (median 85 million years ago [MYA]; 95% confidence interval [CI]: 81*–*94 MYA), *P. tremula–A. thaliana* split (median 108 MYA; 95% CI: 97*–*109 MYA) and *O. sativa–O. europaea* split (median 149 MYA; 95% CI: 148*–*­173 MYA). The phylogenetic tree was visualized in FigTree v. 1.4.3 [61].

## Results and Discussion

### Genomic karyotype analysis and size estimation

Morphometric analysis of the chromosome pairs revealed that the chromosome length ranged from 1.93 to 5.07 µm, with an arm ratio ranging from 1.02 to 2.26. Nine chromosome pairs (chromosomes 2, 5, 8, 9, 10, 11, 12, 13, 14) were m, and 6 (chromosome 1, 3, 4, 6, 7, 15) were sm. An obvious satellite was found to be located at the second pair of the 1 chromosome pairs. Genomic karyotype analysis showed that yellowhorn “WF18” was a diploid plant with karyotype formula 2n = 2X = 30 = 18m (2SAT) + 12 sm (Fig. S1).

The genomic size was also estimated on the basis of the *k*-mer frequency spectrum with different *k*-mer length ranging from 17 to 200. A *k*-mer statistics algorithm of KMC was introduced to count and manipulate *k*-mer sizes. With *k*-mer length of 61, the genomic size was estimated to be 442.33 Mb with a relatively high heterozygosis rate of 0.81% (Fig. S2). The haploid genome size of yellowhorn was also measured by means of flow cytometry and showed a 1C genomic sequence of 433.57 Mb.

### Genome sequencing and assembly

**Figure 2:**
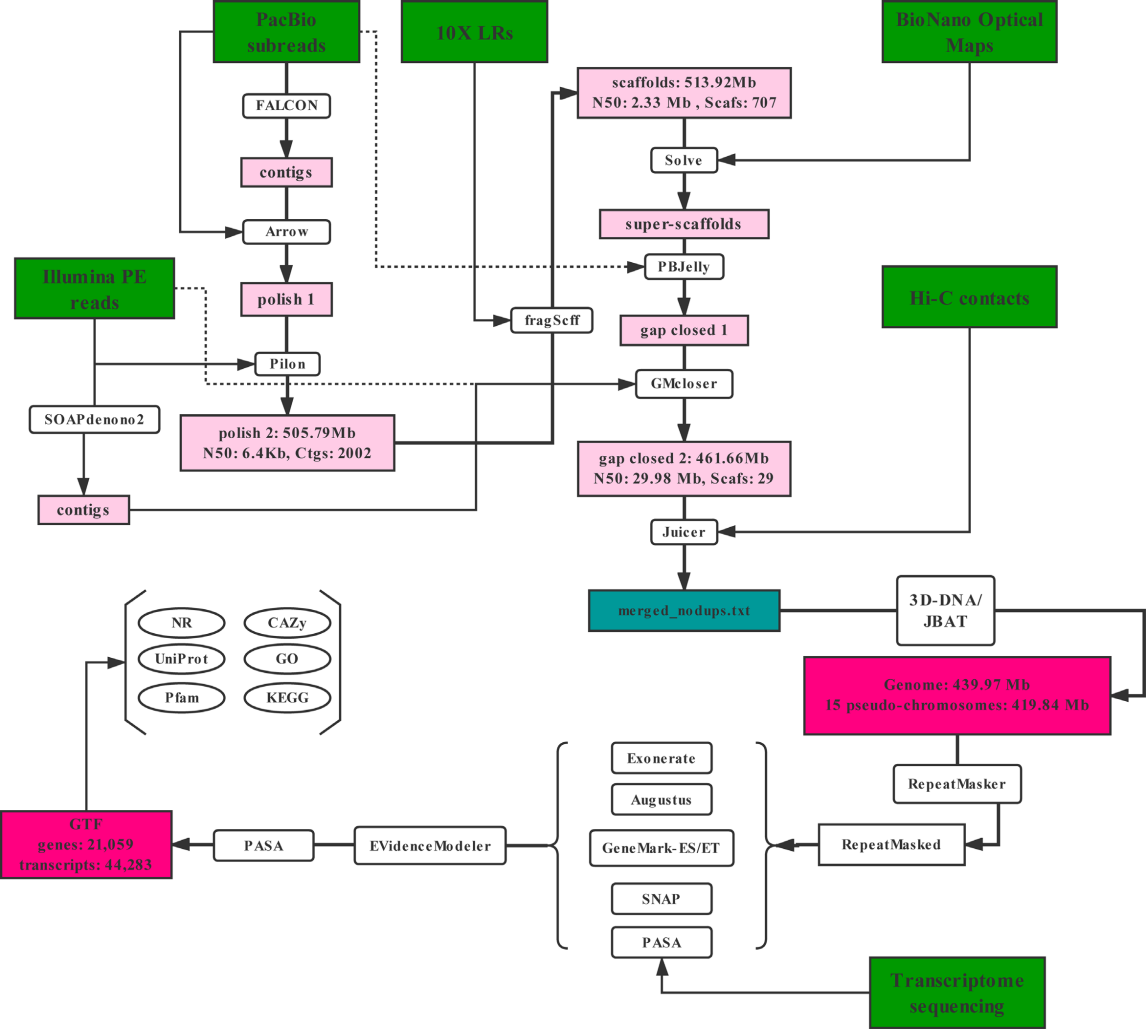
Flowchart of genome assembly and annotation. GTF: gene transfer format; LR: long read; PE: paired end.

The flow chart of genome assembly and annotation is shown in Fig. 2. The yellowhorn genome was assembled by integration of Illumina short reads, PacBio long reads, 10X Genomics link reads, Bionano optical maps2, and Hi-C short reads.

#### PacBio long-read assembly

Using PacBio long-read sequencing, ∼70.62 Gb high-quality subreads were generated from a 20-kb DNA sequencing library with mean length >8 kb and N50 length >15 kb (Table 2). By finding a single path of each contig graph with optimal parameter “length_cutoff 2000 and length_cutoff_pr 8000” using the graph to contig script “fc_graph_to_contig.py”, the draft genomic contigs were created to be 505.79 Mb in length with N50 value of 642,338 bp for 2,002 contigs (Table 3). Assembled contigs were polished with PacBio long reads and high-quality Illumina paired-end reads, resulting in 2,002 assembled contigs with 508.45 Mb in length and N50 value of 645,453 bp (Table 3).

**Table 2:** Statistics of PacBio Sequel sequencing data

Index	PacBio
Total No. of reads	7,062,244
Mean length of raw reads (bp)	226,712
N50 of raw reads (bp)	374,500
Mean length of subreads (bp)	156,717
N50 of subreads (bp)	237,539
Coverage (X)*	160.51

^*^Coverage (X) = (read count * read length)/estimated genome size.

**Table 3:** Summary of yellowhorn genome assembly

Statistics	Contigs	Contigs (polished)	10X Genomics	BioNano	Hi-C	
					Scaffold	Chromosome
Total No.	2,002	2,002	707	29	267	15
Length (bp)						
Total	505,787,109	508,445,799	513,924,146	461,662,473	439,965,977	419,835,445
N50	642,338	645,453	2,334,658	29,979,918	29,432,808	29,432,808
N90	113,799	114,103	492,748	15,941,042	17,893,618	17,893,618
Maximum	4,375,484	4,395,303	21,312,255	75,772,594	39,123,600	39,123,600
GC content (%)	35.25	35.13	34.67	32.39	32.76	34.18

#### Pseudo-chromosome construction

**Figure 3:**
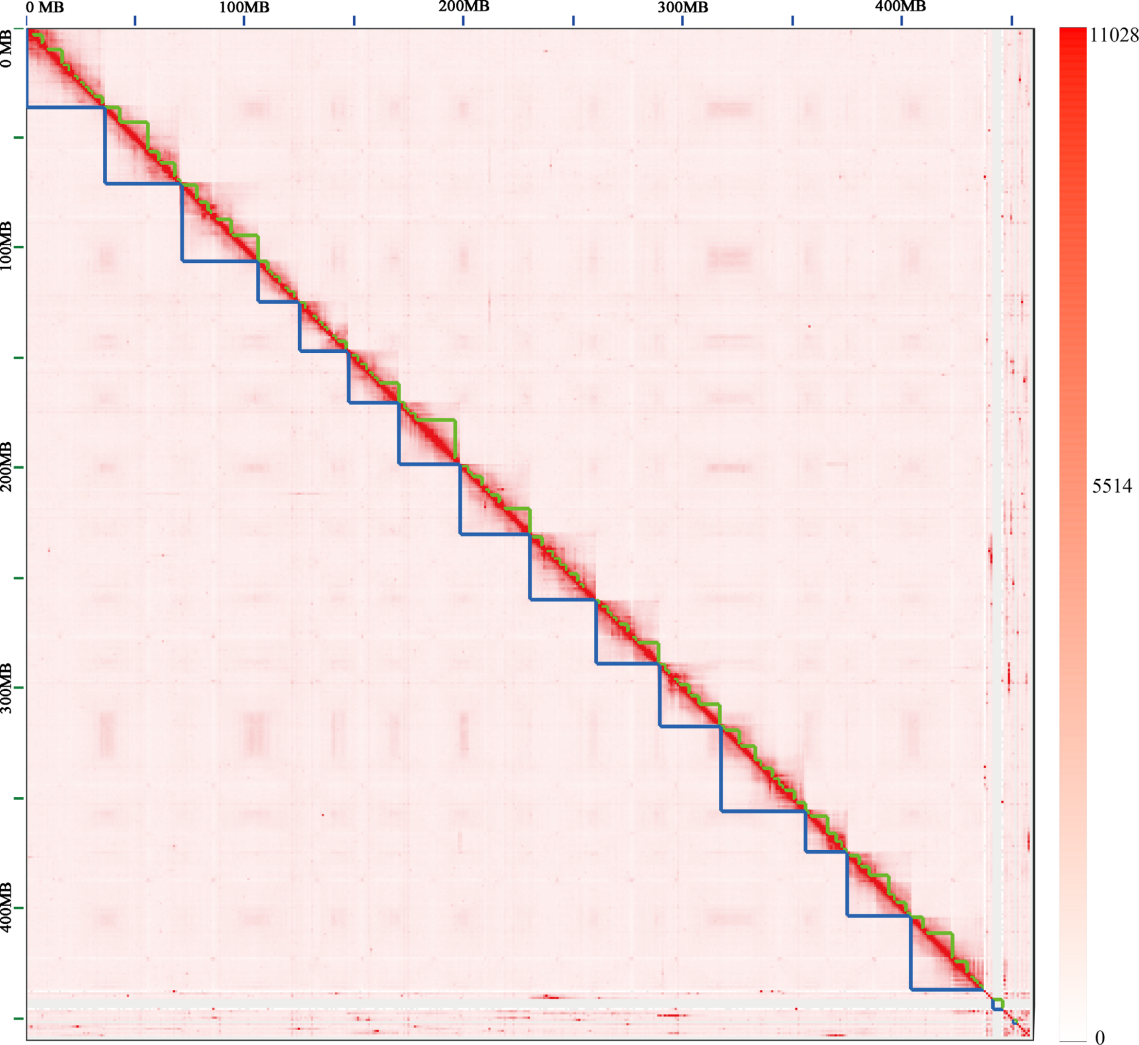
Contact maps of Hi-C links among chromosomes. The blue squares represent the draft scaffold. The green squares represent the chromosome-length superscaffold. The color bar illuminates the Hi-C contact density in the plot.

**Figure 4:**
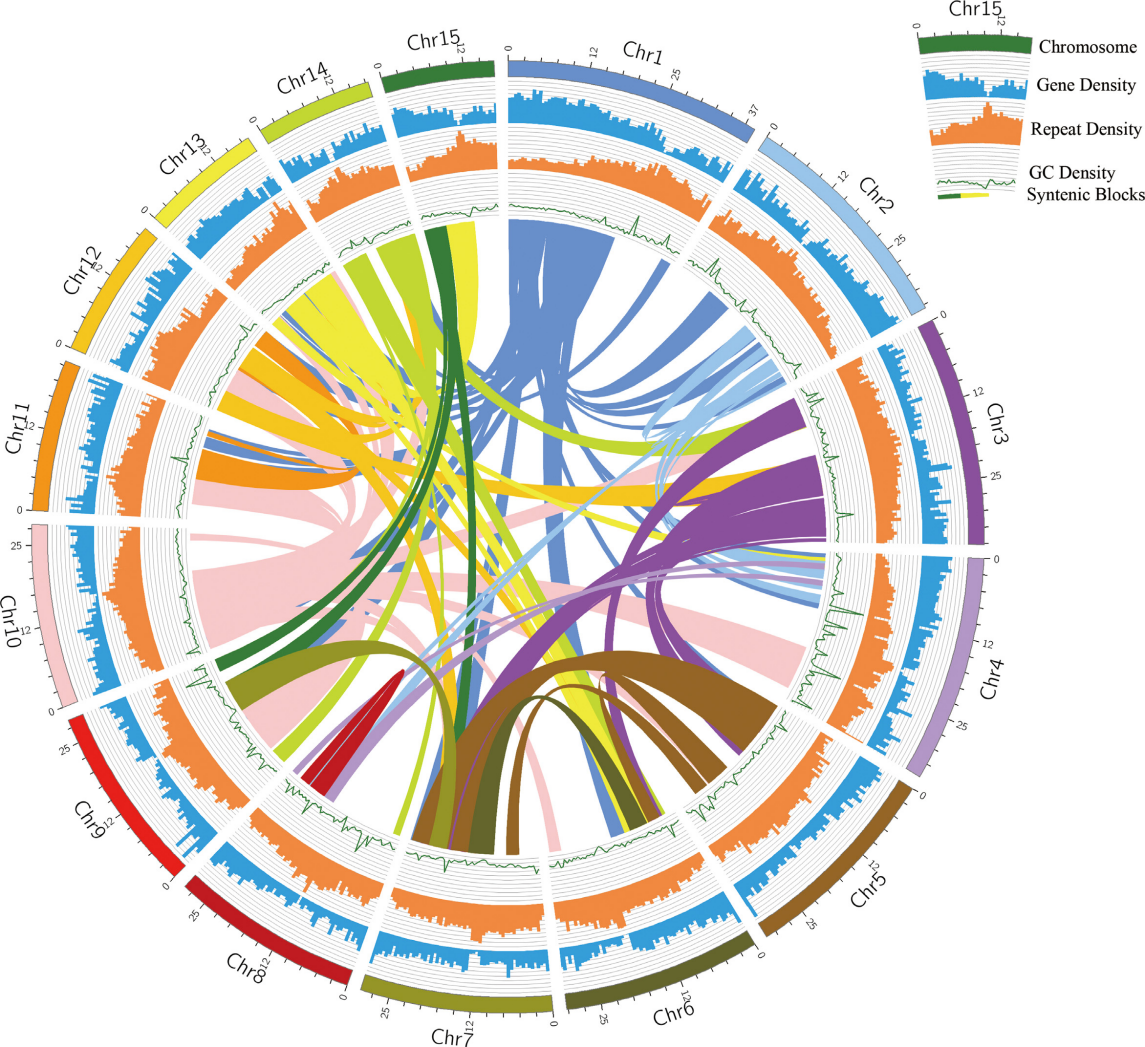
Yellowhorn genome features. The chromosome size is shown in Mb scale. The syntenic blocks are represented by curves in the center of the graph. The figure was created using the circos software package v. 0.69. GC: guanine-cytosine.

The polished contigs were scaffolded with the 10X Genomics linked reads and assembled to be 513.92 Mb in length with N50 value of 2,334,658 bp for 707 scaffolds (Table 3). By hybridizing the 2 BioNano Genomic maps with the *in silico* maps of genome assembly, 29 super-scaffolds were generated with length of 461.66 Mb with N50 value of 29.98 Mb (Table 3). The 7,192 (34.73 Mb) gaps distributed in hybrid super-scaffolds were first filled with PacBio consensus long reads, leading to 6,015 gaps being resolved. Subsequently, the gaps were further filled with Illumina insert size of 280 and 450 bp library paired-end reads, giving rise to the closure of 77 gaps. Overall, 6,092 gaps were filled, which reduced the N bases to 29.06 Mb, representing 6.29% of hybrid super-scaffolds. To get the chromosome length of scaffolds, the *in situ* Hi-C data were used to generate yellowhorn pseudo-chromosomes with 439.97 Mb in final genome assembly size. Fifteen pseudo-chromosomes were assembled, which covered 95.42% (419.84 Mb) of the genome assembly (Fig. 3). The maximal length of the pseudo-chromosomes was 39.12 Mb and minimum was 17.23 Mb (Fig. 4, Table 3, and Table S2).

### Genome assembly assessment

The completeness of the genome assembly was assessed by searching against 1,440 embryophyta-specific single-copy orthologs in genome assembly assessment mode using BUSCO v. 3.0.2 (BUSCO, RRID:SCR_015008) [62] with default parameters. In total, 1,218 (84.58%) complete BUSCOs and 23 (1.60%) fragmented BUSCOs were identified in the yellowhorn genome (Table 4). A total of 85.10% *de novo* assembled RNA-sequencing transcripts of 5 tissue types were mapped to the yellowhorn genome using BLAT v. 3.2.19 (BLAT, RRID:SCR_011919) [63] with identity ≥98% and coverage ≥50% of each transcript. The genome assembly was also evaluated by QUAST v. 5.0.0 [64] with default parameters. The result showed an NG50 value of 28.89 Mb, which represents that the length of contigs covering at least half of genome assembly was close to the N50 value of 29.43 Mb. This indicates that the genome assembly was of high quality (Table S3).

**Table 4:** BUSCO assessment of yellowhorn genome assembly

Description	No. (%)
Complete BUSCOs (C)	
Complete and single-copy BUSCOs (S)	1,175 (81.60)
Complete and duplicated BUSCOs (D)	43 (2.98)
Fragmented BUSCOs (F)	23 (1.60)
Missing BUSCOs (M)	199 (13.82)
Total BUSCO groups	1,440 (100)

### Genome characterization

The repetitive fractions represented 56.39% of the yellowhorn genome assembly, with repetitive elements and simple sequence repeats (SSRs) accounting for 54.81% and 1.58%, respectively. Therefore, comparing the content of repetitive elements with other reported closely related species revealed that the content of repeat fractions in the current yellowhorn genome assembly was relatively higher than that of *A. thaliana* (13.2%) [65], *Thellungiella salsuginea* (52%) [66], *Brassica oleracea* (48.8%) [67], *Arabidopsis lyrata* (35%) [68], *Brassica napus* (55.59%) [69], *Citrus sinensis* (20.5%) [70], *Theobroma cacao* (25.7%) [71], *D. longan* (52.87%) [15], and *Durio zibethinus* (54.8%) [72] but lower than that of *Gossypium raimondii* (57%) [73]. Moreover, LTR/Copia and LTR/Gypsy repeats were the most abundant repetitive elements, accounting for 11.91% and 11.68% of the assembled genome, respectively (Table 5).

**Table 5:** Repeat content of yellowhorn genome assembly

Category	Term	Length (bp)	Percentage of genome (%)
DNA transposons	DNA	374,909	0.09
	DNA/CMC-EnSpm	1,699,637	0.39
	DNA/MuLE-MuDR	3,896,024	0.89
	DNA/PIF-Harbinger	1,104,979	0.25
	DNA/TcMar-Pogo	94,067	0.02
	DNA/hAT-Ac	4,103,980	0.93
	DNA/hAT-Tag1	890,950	0.20
	DNA/hAT-Tip100	1,213,576	0.28
SINEs	SINE	343	0
	SINE/tRNA	10,674	0
LINEs	LINE/L1	16,861,661	3.83
LTRs	LTR	2,861	0
	LTR/Caulimovirus	1,360,538	0.31
	LTR/Copia	52,384,264	11.91
	LTR/Gypsy	51,370,228	11.68
	LTR/Pao	88	0
Low_complexity		1,516,978	0.34
RCs		4,215	0
RC/Helitron		5,949	0
Ribosomal RNA		64,618	0.01
SSRs		6,971,711	1.58
Unknown		104,792,508	23.76
Total		248,724,758	56.39
Genome size		439,965,977	100

LINE: long interspersed nuclear element; LTR: long terminal repeat; RC: rolling circle replication; SINE: short interspersed nuclear element; SSR: simple sequence repeat.

To annotate the yellowhorn genome for protein-coding genes, a comprehensive strategy was implemented that integrated *ab initio* predictors, protein homology searches, and *de novo* assembled transcripts. After *ab initio* gene prediction with the trained optimal parameters, 20,980 genes from Augustus prediction, 28,134 genes from SNAP, and 32,205 genes from GeneMark-ES/ET were predicted. For protein-based homology searches, 61,138 protein sequences were collected and spliced aligned to the yellowhorn genome assembly to get homology gene sets. A total of 21,157 predicted genes were obtained by integration of all gene sets using EVM. After UTR updating by running PASA on 3 rounds, 21,059 predicted protein coding genes with 44,283 transcripts were obtained in the final gene models. Among these gene sets, 20,952 gene models with 44,078 transcripts were allocated in the 15 pseudo-chromosomes. All transcripts had a mean length of ∼7,040 bp, a mean coding sequence length of 201.62 bp, and a mean of 15.61 exons per gene model. To explore the function of predicted gene models, all predicted genes were annotated by searching against the NR, UniProt, Pfam, GO, KEGG, and CAZy databases. Finally, 18,503 gene models accounting for 87.86% of all gene sets were functionally annotated with ≥1 term.

### Comparative phylogenomics

**Figure 5:**
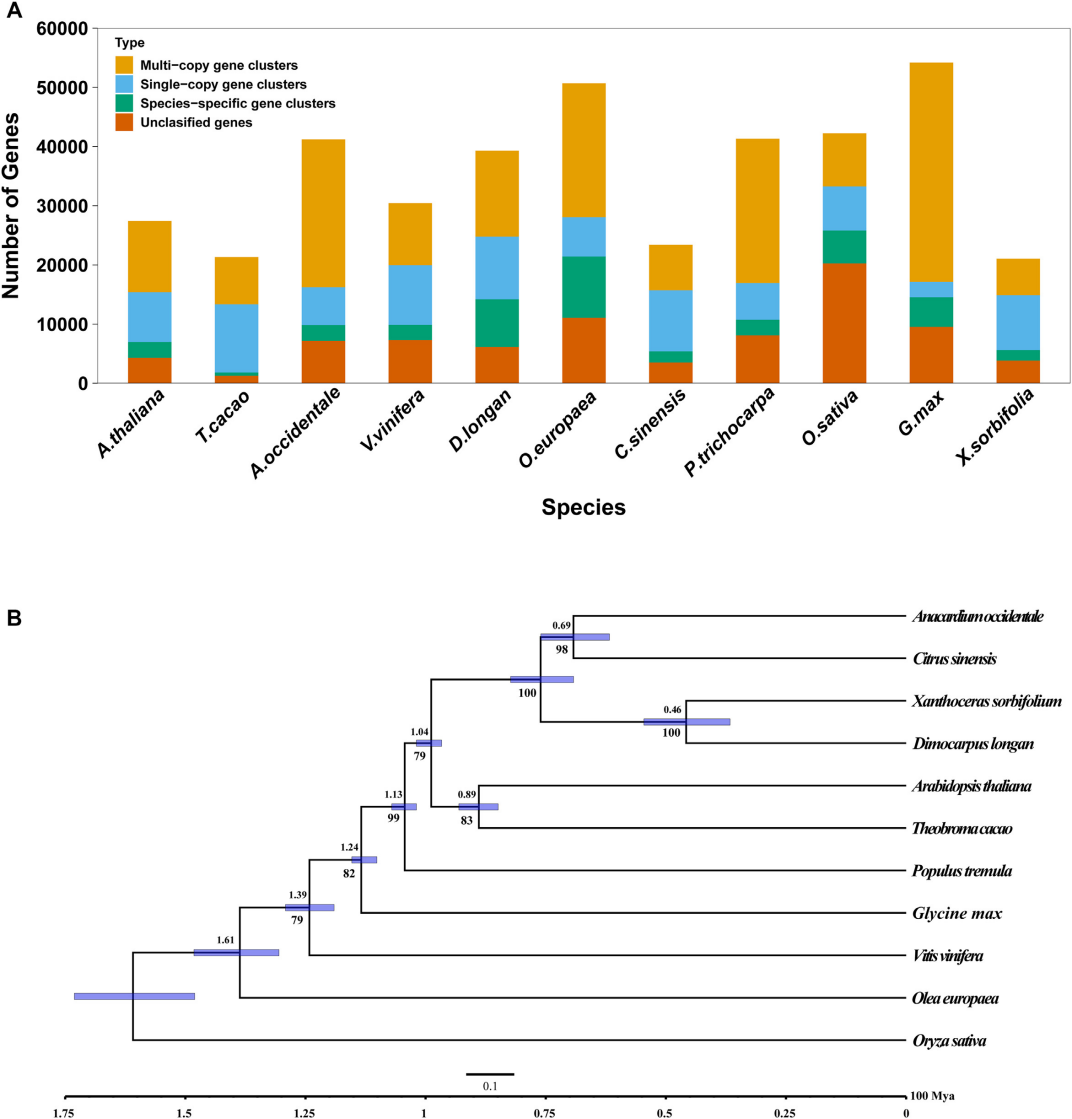
Phylogenomics analysis of yellowhorn genome. (A) OrthoMCL clusters of yellowhorn and 10 other species. (B) Phylogenetic tree and estimated divergence time of yellowhorn and 10 other species. The numbers above the branches are the predicted divergence time. The numbers below the branches are the bootstrap support value. The light blue bars at the internodes represent the 95% confidence interval. The bottom scale bar shows divergence time, with 1 time unit representing 100 MYA.

Gene families were clustered on the basis of yellowhorn and other plant species using OrthoMCL. In total, 27,347 groups were constructed, of which 5,484 groups contained sequences from all species, 1,496 groups from ≥2 species, and 10,367 groups from only 1 species (Fig. 5a). Meanwhile, 462 groups containing 1,789 genes were further identified as yellowhorn specific. GO enrichment by topGO showed that “oxidation-reduction process” (*P* = 1.7 × 10^­10^), “defense response” (*P* = 1.8 × 10^−6^), “oxidoreductase activity” (*P* = 5.8 × 10^−12^), and “membrane” (*P* = 6.5 × 10^−6^) were the extremely significantly enriched function categories (Table S4).

The 195 single-copy orthologous genes in the yellowhorn genome assembly and 10 other plant species were used to investigate the evolution of yellowhorn (Table S5). RaxML was used to construct phylogenetic trees with the evolutionary model GTR+GAMMA. The divergence time was estimated using MCMCTree in 5 independent MCMCTree runs and extrapolated using secondary calibration points from the TimeTree database. The phylogenetic tree was visualized in FigTree and suggested that yellowhorn and *D. longan* diverged from their most recent common ancestor approximately median 46 MYA (95% CI, 36.64–54.58 MYA) (Fig. 5b).

### Transcriptome analysis of tissue-specific expression

**Figure 6:**
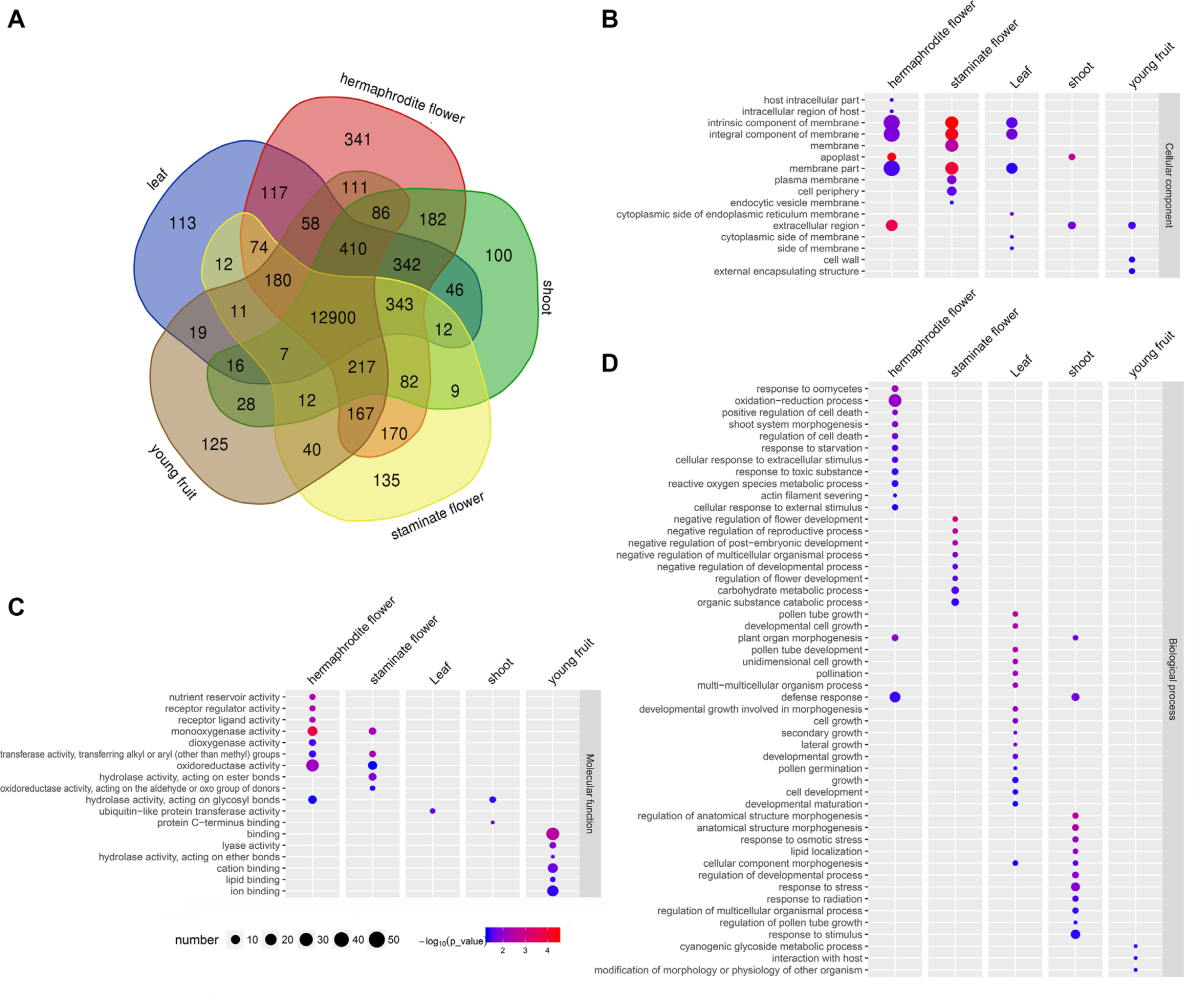
Tissue-specific gene analysis. (A) Venn diagram showing shared and unique genes among 5 tissues. Numbers represent the number of genes that were unique or shared. (B−D) GO enrichment of tissue-specific genes. The node size represents the gene numbers enriched in each GO category. The color bar illuminates *P*-value from red (low) to blue (high) in the plot.

To explore the tissue-specific genes, we performed transcriptomic analysis of 5 yellowhorn tissues including hermaphrodite flower, staminate flower, young fruit, leaf, and shoot. A total of 814 tissue-specific genes including 45 transcription factors were obtained. Of these, 341, 135, 113, 125, and 100 genes were specifically expressed in hermaphrodite flower, staminate flower, young fruit, leaf, and shoot, respectively (Fig. 6a, Table S6). GO enrichment of hermaphrodite flower−specific genes showed that the functions of “oxidation-reduction process” (*P* = 8.83 × 10^−3^), “defense response” (*P* = 3.87 × 10^−2^), “monooxygenase activity” (*P* = 1.6 × 10^−4^), “oxidoreductase activity” (*P* = 6.78 × 10^−3^), and “membrane part” (*P* = 3.47 × 10^−2^) were significantly enriched. “Growth related” (*P* = 3.6 × 10^−3^) and “membrane part” (*P* = 4.4 × 10^−2^) were significantly enriched functions in leaf. For shoot-specific genes, “response to stress” (*P* = 1.31 × 10^−2^), “regulation of developmental process” (*P* = 1.16 × 10^−2^), and “extracellular region” (*P* = 1.9 × 10^−2^) were significantly enriched. The GO terms of “negative regulation of flower development and reproductive process” (*P* = 4.9 × 10^−4^), “oxidoreductase activity” (*P* = 4.99 × 10^−2^), and “membrane” (*P* = 3.0 × 10^−3^) were mostly enriched in staminate flower. Additionally, GO enrichment of young fruit-specific genes showed that “metabolic process” (*P* = 4.3 × 10^−2^), “binding” (*P* = 2.7 × 10^−3^), and “lyase activity” (*P* = 1.29 × 10^−2^) were the most enriched functions (Fig. 6b, Table S7).

Functions of the specific genes revealed correlate well with the biological roles of the tissues found by previous studies. For instance, hermaphrodite flower contains both stamens and pistils and gives rise to fruits after fertilization [74]. Consistently, a number of hermaphrodite flower−specific genes have been shown to be involved in gametophytic development, fertilization, and seed development. Of them, *AGL66* (XS01G01870) is expressed preferentially in pollen and participates in the regulation of male gametophytes in the model plant Arabidopsis. Double mutations of *AGL66* and *AGL104* lead to decreased pollen viability [75]. *MYB39* (XS01G02268) is involved in microsporogenesis in apple (*Malus domestica*); e.g., suppressing *MYB39* expression in pollen reduced pollen tube growth [76]. *MYB64* (XS02G10689), together with *MYB119*, regulates cellularization and differentiation during female gametogenesis becuase ametophytes of *myb64 myb119* double mutant fail to initiate the FG5 transition, giving rise to uncellularized gametophytes with supernumerary nuclei [77]. Moreover, egg cell−secreted protein 1, which is known as EC1 (XS05G15286), is responsible for sperm activation during fertilization [78]. *Exo70A1* (XS05G14582), which encodes a putative exocyst subunit, regulates both pollen-pistil interaction and localized deposition of seed coat pectin [79, 80]. *AGL62* (XS03G11771) encodes a MADS domain transcription factor, controlling cellularization during endosperm development [81]. Another MADS gene, *PHERES1* (XS14G07914), has also been proven to be involved in seed development [82]. In addition, *DIVARICATA* (XS07G17645), a MYB family transcription factor controlling the dorsoventral asymmetry of flowers in *Antirrhinum*, was specifically expressed in hermaphrodite flower, implying that the regulatory mechanisms underlying corolla formation in the 2 flower types of yellowhorn might be different [83]. These results indicate that identification and analyses of tissue-specific genes provided clues for understanding the molecular functions of separate tissues of yellowhorn.

## Availability of supporting data and materials

Raw data are available via NCBI (Bioproject accession PRJNA496350, Biosample: SAMN10239523). Other supporting data, including the genome assembly, annotations, VCF files, and alignments, are available via the *GigaScience* database, GigaDB [84]. For a list of software resources utilised see Table 6.

**Table 6:** software resources list.

**Software**	**URLs**
FASTQC	http://www.bioinformatics.babraham.ac.uk/projects/fastqc/
Trimmomatic	http://www.usadellab.org/cms/index.php?page=trimmomatic/
FALCON	https://github.com/PacificBiosciences/FALCON/
pbalign	https://github.com/PacificBiosciences/pbalign/
arrow	https://github.com/PacificBiosciences/GenomicConsensus/
BWA	http://bio-bwa.sourceforge.net/
fragScaff	https://sourceforge.net/projects/fragscaff/
Solve	https://bionanogenomics.com/support-page/bionano-solve/
PBJelly	https://sourceforge.net/projects/pb-jelly/files/latest/download
GMcloser	https://sourceforge.net/projects/gmcloser/
Juicer	https://github.com/aidenlab/juicer/
BUSCO	https://busco.ezlab.org/
QUAST	http://quast.bioinf.spbau.ru/
RepeatMasker	http://repeatmasker.org/
RepeatModeler	http://www.repeatmasker.org/RepeatModeler/
Trinity	https://github.com/trinityrnaseq/trinityrnaseq/
PASA	https://github.com/PASApipeline/PASApipeline/
Augustus	http://bioinf.uni-greifswald.de/augustus/
SNAP	https://github.com/KorfLab/SNAP/
GeneMark-ES/ET	http://exon.gatech.edu/GeneMark/
Exonerate	https://www.ebi.ac.uk/about/vertebrate-genomics/software/exonerate
EVidenceModeler	http://evidencemodeler.github.io/
OrthoMCL	http://orthomcl.org/orthomcl/
topGO	http://bioconductor.org/packages/topGO/
MAFFT	https://mafft.cbrc.jp/alignment/software/
RaxML	http://evomics.org/learning/phylogenetics/raxml/
PAML	http://abacus.gene.ucl.ac.uk/software/paml.html/
Tophat	http://ccb.jhu.edu/software/tophat/index.shtml/
GenomeScope	http://qb.cshl.edu/genomescope/
KMC	http://sun.aei.polsl.pl/kmc/
**Reference data**	**URLs**
RepBase plant repeat database	https://www.girinst.org/server/RepBase/
TimeTree database	http://timetree.org/
UniProt plant protein database	ftp://ftp.uniprot.org/pub/databases/uniprot/current_release/knowledgebase/taxonomic_divisions/uniprot_sprot_plants.dat.gz
NR	ftp://ftp.ncbi.nlm.nih.gov/blast/db/FASTA/nr.gz
UniProt	ftp://ftp.uniprot.org/pub/databases/uniprot/current_release/knowledgebase/taxonomic_divisions/uniprot_sprot_plants.dat.gz
Pfam database	ftp://ftp.ebi.ac.uk/pub/databases/Pfam/releases/Pfam28.0/Pfam-A.hmm.gz
CAZy database	http://csbl.bmb.uga.edu/dbCAN/download.php
*Olea europaea* v1	http://olivegenome.org/downloads/
*Citrus sinensis* v2	http://citrus.hzau.edu.cn/orange/download/index.php
*Glycine max* v9.0	ftp://ftp.jgi-psf.org/pub/compgen/phytozome/v9.0/Gmax/
*Arabidopsis thaliana* TAIR10	https://www.arabidopsis.org/download/index-auto.jsp?dir=%2Fdownload_files%2FGenes%2FTAIR10_genome_release
*Oryza sativa* IRGSP-1.0	http://rapdb.dna.affrc.go.jp/download/irgsp1.html
*Populus trichocarpa* v3.0	https://genome.jgi.doe.gov/pages/dynamicOrganismDownload.jsf?organism=Ptrichocarpa
*Vitis vinifera* v2	http://genomes.cribi.unipd.it/grape/
*Dimocarpus longan*	ftp://penguin.genomics.cn/pub/10.5524/100001_101000/100276/
*Anacardium occidentale* v0.9	https://genome.jgi.doe.gov/portal/pages/dynamicOrganismDownload.jsf?organism=Aoccidentale
Theobroma cacao v2	http://cocoa-genome-hub.southgreen.fr/download

## Editor's Note

Please also note another, independent Data Note published in *GigaScience*, also presenting a genome assembly of *Xanthoceras sorbifolium* has been reviewed and is being published alongside this one [85]. on the same day.

## Additional files

**Figure S1**. Karyogram of yellowhorn superior “WF18.” (A) Chromosome at diakinesis of pollen mother cell meiophase. Bar = 5 μm. (B) Yellowhorn superior “WF18” was a diploid plant, 2n = 2X = 30. (C) Ideogram (karyotype formula of yellowhorn superior “WF18” was 2n = 2X = 30 = 18m (2SAT) +12 sm).

**Figure S2**. Yellowhorn genome evaluation and estimation by GenomeScope. The x-axis represents *k*-mer coverage. The *y*-axis represents *k*-mer frequency spectrum numbers. With *k*-mer length of 61, the genomic size was estimated to be 442.33 Mb with a heterozygosis rate of 0.81%.

**Table S1**. Statistics of transcriptome sequencing data.

**Table S2**. The features of the yellowhorn genome assembly.

**Table S3**. Genome quality control report of yellowhorn genome assembly by QUAST.

**Table S4**. GO enrichment of yellowhorn-specific genes.

**Table S5**. The 195 single-copy orthologous genes in the yellowhorn genome assembly and 10 other species.

**Table S6**. Yellowhorn tissue-specific genes.

**Table S7**. GO enrichment of yellowhorn tissue-specific genes.

giz071_GIGA-D-18-00410_Original_Submission

giz071_GIGA-D-18-00410_Revision_1

giz071_GIGA-D-18-00410_Revision_2

giz071_Response_to_Reviewer_Comments_Original_Submission

giz071_Response_to_Reviewer_Comments_Revision_1

giz071_Reviewer_1_Report_Original_Submission John Mackay, Ph.D. -- 11/10/2018 Reviewed

giz071_Reviewer_2_Report_Original_Submission Laura Kelly -- 11/24/2018 Reviewed

giz071_Reviewer_2_Report_Revision_1 Laura Kelly -- 3/29/2019 Reviewed

giz071_Reviewer_3_Report_Original_Submission Pedro Martinez Garcia -- 11/28/2018 Reviewed

giz071_Supplemental_Files

## Abbreviations

BCL: binary base call; BLAST: Basic Local Alignment Search Tool; bp: base pairs; BUSCO: Benchmarking Universal Single-Copy Orthologs; BWA: Burrows-Wheeler Aligner; CAZy: Carbohydrate-Active enZYmes; CI: confidence interval; CL: chromosome length; DPF: days post flower; dUTP: deoxyuridine triphosphate; EVM: EVidenceModeler; Gb: gigabase pairs; GC: guanine-cytosine; GO: Gene Ontology; GTF: gene transfer format; Hi-C: high-throughput chromosome conformation capture; HISAT2: hierarchical indexing for spliced alignment of transcripts 2; HSL: haploid set length; JBAT: Juicebox Assembly Tools; kb: kilobase pairs; KAAS: KEGG Automatic Annotation Server; KEGG: Kyoto Encyclopedia of Genes and Genomes; KMC: K-mer Counter; LINE: long interspersed nuclear element; LL: long arm length; LR: long read; LTR: long terminal repeat; MAFFT: Multiple Alignment using Fast Fourier Transform; Mb: megabase pairs; MYA: milion years ago; NCBI: National Center for Biotechnology Information; NR: Nonredundant; ORF: open reading frame; PacBio: Pacific Biosciences; PAML: Phylogenetic Analysis Using Maximum Likelihood; PASA: Program to Assemble Spliced Alignments; PE: paired end; RAxML: Randomized Axelerated Maximum Likelihood; QUAST: Quality Assessment Tool for Genome Assemblies; RC: rolling circle replication; SINE: short interspersed nuclear element; SL: short arm length; SNAP: Semi-HMM-based Nucleic Acid Parser; SSR: simple sequence repeat; UTR: untranslated region.

## Competing interests

The authors declare that they have no competing interests.

## Funding

This work was financially supported by the Improved Variety Program of Shandong Province of China (2016LZGC013), the Innovative Project of Forestry Science and Technology of Shandong Province of China (LYCX05-2018-26), and the Funds of Shandong “Double Tops” Program (SYL2017XTTD09).

## Authors’ contributions

K.Q.Y. conceived this genome project and coordinated research activities; L.Y., J.N.L., K.Q.Y., Y.L.S., and N.W. designed the experiments; L.Y., J.N.L., H.L., Q. Liang, and X.H. assembled and annotated the genome; H.L., Q. Li, R.Z., and X.H. analyzed transcriptome and phylogenies; Q. Liang, S.L., F.Y., and Q.D. collected and maintained plant materials; J.S. estimated genome size and analyzed karyotype. J.N.L., L.Y., K.Q.Y., Q. Liang, H.L., Y.L.S., and N.W. wrote the manuscript. All authors have read and approved the final manuscript.
