# Supplementary material for: The genome assembly and annotation of yellowhorn (Xanthoceras sorbifolium Bunge)
Source: Gigascience. 2019 Jun 26;8(6):giz071. doi: 10.1093/gigascience/giz071 (PMC6593362; doi:10.1093/gigascience/giz071)
Supplement: giz071_GIGA-D-18-00410_Original_Submission [file giz071_giga-d-18-00410_original_submission.pdf]

# GigaScience

# The genome assembly and annotation of yellowhorn (Xanthoceras sorbifolium Bunge)

--Manuscript Draft--

| | |
|---|---|
| **Manuscript Number:** | GIGA-D-18-00410 |
| **Full Title:** | The genome assembly and annotation of yellowhorn (Xanthoceras sorbifolium Bunge) |
| **Article Type:** | Research |
| **Funding Information:** | the Improved Variety Program of Shandong Province of China (2016LZGC013) — Mr. Keqiang Yang<br>the Innovative Project of Forestry Science and Technology of Shandong Province of China (LYCX05-2018-26) — Mr. Keqiang Yang<br>the Funds of Shandong 'Double Tops' Program (SYL2017XTTD09) — Mr. Keqiang Yang |


**Abstract:**

Background

Yellowhorn (Xanthoceras sorbifolium Bunge), a deciduous shrub or small tree native to north China, is of great economic values. Seeds of yellowhorn are rich in oil containing unsaturated long chain fatty acids that have been used for producing edible oil and nervonic acid capsule. However, the lack of a high-quality genome sequence hampers the understanding of its evolution and gene functions.

Findings

In this study, a whole-genome of yellowhorn was sequenced and assembled by integration of Illumina sequencing, PacBio single-molecule real-time sequencing, 10X Genomics link-reads, Bionano optical maps and Hi-C. The yellowhorn genome assembly was 439.97 Mb, which comprised of 15 pseudo-chromosomes covering 95.42% (419.84 Mb) of the genome. The repetitive fractions accounted for 56.39% of yellowhorn genome. The genome contained 21,059 protein coding genes. Of them, 18,503 (87.46%) genes were functionally annotated at least one term by searching against the other databases. Transcriptomic analysis showed that 341, 113, 100, 135 and 125 genes were specifically expressed in leaf, hermaphrodite flower, shoot, staminate flower and young fruit, respectively. Phylogenetic analysis suggested that yellowhorn diverged from the common ancestral of Dimocarpus longan approximately 58.63 million years ago.

Conclusions

The availability and subsequent annotation of yellowhorn genome, as well as the identification of tissue-specific functional genes, provides a valuable reference for plant comparative genomics, evolutionary studies and molecular design breeding.




| | |
|---|---|
| **Corresponding Author:** | Keqiang Yang, Ph.D.<br>Shandong Agricultural University<br>Tai'an, Shandong Province CHINA |
| **Corresponding Author Secondary Information:** | |
| **Corresponding Author's Institution:** | Shandong Agricultural University |
| **Corresponding Author's Secondary Institution:** | |
| **First Author:** | Qiang Liang |
| **First Author Secondary Information:** | |
| **Order of Authors:** | Qiang Liang |
| | |



*Powered by Editorial Manager® and ProduXion Manager® from Aries Systems Corporation*

| | |
|---|---|
| | Huayang Li |
| | Shouke Li |
| | Fuling Yuan |
| | Jingfeng Sun |
| | Qicheng Duan |
| | Qingyun Li |
| | Rui Zhang |
| | Yalin Sang |
| | Nian Wang |
| | Xiangwen Hou |
| | Keqiang Yang |
| | Jianning Liu |
| | Long Yang |
| **Order of Authors Secondary Information:** | |
| **Additional Information:** | |
| **Question** | **Response** |
| Are you submitting this manuscript to a special series or article collection? | Yes |
| Please select an option from the menu: as follow-up to "Are you submitting this manuscript to a special series or article collection?" | Functional Metagenomics |
| **Experimental design and statistics**<br><br>Full details of the experimental design and statistical methods used should be given in the Methods section, as detailed in our Minimum Standards Reporting Checklist. Information essential to interpreting the data presented should be made available in the figure legends.<br><br>Have you included all the information requested in your manuscript? | Yes |
| **Resources**<br><br>A description of all resources used, including antibodies, cell lines, animals and software tools, with enough information to allow them to be uniquely identified, should be included in the | Yes |

| | |
|---|---|
| Methods section. Authors are strongly encouraged to cite Research Resource Identifiers (RRIDs) for antibodies, model organisms and tools, where possible.<br><br>Have you included the information requested as detailed in our Minimum Standards Reporting Checklist? | |
| **Availability of data and materials**<br><br>All datasets and code on which the conclusions of the paper rely must be either included in your submission or deposited in publicly available repositories (where available and ethically appropriate), referencing such data using a unique identifier in the references and in the "Availability of Data and Materials" section of your manuscript.<br><br>Have you have met the above requirement as detailed in our Minimum Standards Reporting Checklist? | Yes |

Manuscript

Click here to download Manuscript The genome assembly and annotation of yellowhorn.docx

# Title：The genome assembly and annotation of yellowhorn (*Xanthoceras sorbifolium* Bunge)

Qiang Liang[1†], Huayang Li[2†], Shouke Li[3], Fuling Yuan[1], Jingfeng Sun[1], Qicheng Duan[1], Qingyun Li[2], Rui Zhang[2], Yalin Sang[1], Nian Wang[1], Xiangwen Hou[4], Keqiang Yang[1]*, Jianning Liu[4]*, Long Yang[2]*

* **Correspondence**: yangwere@126.com; jnliu@kegene.com; yanglong1020@163.com

† **Equal contributors**

1 College of Forestry, Shandong Agricultural University, Tai'an 271018, China.

2 College of Plant Protection, Shandong Agricultural University, Tai'an 271018, China.

Full list of author information is available at the end of the article

**Abstract**

**Background:** Yellowhorn (*Xanthoceras sorbifolium* Bunge), a deciduous shrub or small tree native to north China, is of great economic values. Seeds of yellowhorn are rich in oil containing unsaturated long chain fatty acids that have been used for producing edible oil and nervonic acid capsule. However, the lack of a high-quality genome sequence hampers the understanding of its evolution and gene functions.

**Findings:** In this study, a whole-genome of yellowhorn was sequenced and assembled by integration of Illumina sequencing, PacBio single-molecule real-time sequencing, 10X Genomics link-reads, Bionano optical maps and Hi-C. The yellowhorn genome assembly was 439.97 Mb, which comprised of 15 pseudo-chromosomes covering 95.42% (419.84 Mb) of the genome. The repetitive fractions accounted for 56.39% of yellowhorn genome. The genome contained 21,059 protein coding genes. Of them, 18,503 (87.46%) genes were functionally annotated at least one term by searching against the other databases. Transcriptomic analysis showed that 341, 113, 100, 135 and 125 genes were specifically expressed in leaf, hermaphrodite flower, shoot, staminate flower and young fruit, respectively.

Phylogenetic analysis suggested that yellowhorn diverged from the common ancestral of *Dimocarpus longan* approximately 58.63 million years ago.

**Conclusions:** The availability and subsequent annotation of yellowhorn genome, as well as the identification of tissue-specific functional genes, provides a valuable reference for plant comparative genomics, evolutionary studies and molecular design breeding.

**Keywords:** Yellowhorn (*Xanthoceras sorbifolium* Bunge); PacBio sequencing; BioNano Genomics; 10X Genomics Chromium; Chromosome conformation capture; Illumina Paired End sequencing

## Data Description

### Background

Yellowhorn (*Xanthoceras sorbifolium* Bunge, NCBI: txid99658), the single species of genus *Xanthocera* (Sapindaceae), is a deciduous shrub or small tree, naturally occurring to hills and slopes in northern China [1-3]. Yellowhorn is resistant to cold, drought, leanness, and salinity [4, 5] and is of important ecological, economic and pharmacological values [6]. Yellowhorn produces capsular fruits with seeds rich in oil (49.77 % – 68.30 % of kernel), which contains 85 – 93 % unsaturated fatty acids, especially nervonic acid [5, 7]. Stems and fruits of yellowhorn were used in folk medicine in Inner Mongolia for the treatment of rheumatism, gout and enuresis of children [8]. Moreover, different yellowhorn tissues contain multiple bioactive compounds, including triterpenoid saponins, coumarins and avonoids, which have been found to possess the antitumor and anti-inflammatory activities, as well as the potentiality against Alzheimer's disease [8-12].

The Sapindaceae family (also known as the Soapberry family), comprises of 142 genera and 1900 species including important tropical fruits and woody oil-bearing plant, such as *Dimocarpus longan*, *Litchi chinensis*, *Nephelium lappaceum*, *Sapindus mukorossi* and yellowhorn [13, 14]. The genome of

*D. longan* has been sequenced and assembled recently [15]. The chloroplast genome of yellowhorn has been assembled and characterized using Illumina pair-end sequencing data [16]. Genes regulating oil accumulation and fertilized ovules development have been identified in yellowhorn [17, 18]. Despite the increasing availability of genetic resources with research and economic values, fully annotated genome is currently unavailable for yellowhorn.

In this study, a high-quality draft genome of yellowhorn was sequenced and assembled by integration of Illumina sequencing, PacBio single-molecule real-time sequencing, 10X Genomics link-reads, Bionano optical maps and Hi-C. Functionally annotation for protein coding genes was performed. Tissue-specific genes were identified and analyzed through transcriptomic approaches. Our study will facilitate to comparative genomics, gene-functional studies and molecular assisted breeding in the near future.

**Sampling and sequencing**

Yellowhorn superior tree ‘WF18’, with high yield and oil content, from the Forestry Experimental Station of Shandong Agricultural University (36˚10’16’’ E, 117˚08’56’’ N) was selected for genome sequencing (Figure 1). Genomic DNA was extracted from young leaves using NucleoSpin Plant II (MachereyeNagel, Düren, Germany). The quality and quantity of DNA was assessed using 0.8% agarose gels and Qubit fluorimeter (Invitrogen, Carlsbad, CA, USA).

The yellowhorn whole-genome was sequenced and assembled by integration of Illumina sequencing, PacBio single-molecule real-time sequencing, 10X Genomics link-reads, Bionano optical maps and Hi-C (Figure 2). For Illumina sequencing, two libraries with insert sizes of 280 bp and 450 bp were constructed using NEBNext Ultra II DNA Library Prep Kit (New England Biolabs, Ipswich, MA, UK). The libraries were then sequenced on an Illimina HiSeq X Ten System using a PE-150 module and 172

Gb raw data were generated. The quality of all raw reads was assessed using FASTQC. The adaptors and low-quality bases were trimmed using Trimmomatic v. 0.38 with default parameters [19]. Approximate 165 Gb (about 383×) clean reads was obtained for pre-*de novo* genome assembly (Table 1).

For PacBio long reads sequencing, a 20-kb single-molecule real-time DNA sequencing library was constructed according to the manufacturer's protocol (Pacific Biosciences, Menlo Park, CA, USA). The libraries were used for sequencing on the PacBio Sequal platform and yielded over 70 Gb (about 159×) subreads with mean length >8 kb and N50 length >15 Kb (Table 2).

The library of 10X Genomics was prepared using the Chromium Gel Bead and Library Kit (10X Genomics, Pleasanton, CA, USA) and the Chromium instrument (10X Genomics, Pleasanton, CA, USA) following the manufacturer's protocol. The barcoded library was sequenced on an Illumina NovaSeq 6000 system. The BCL files were demultiplexed and converted to fastq files using Supernova mkfastq (v. 2.0.0). After reads trimming, around 457.4 Mb reads with a mean length of 138.5 bp were generated. The fraction of Q30 in read 2 was 83.42% (Table 1).

Two Bionano optical maps were analyzed with Saphyr's streamlined workflow (BioNano Genomics, San Diego, CA, USA). High-molecular-weight DNA was treated with Nt. BspQI and Nt. BssSI nicking endonucleases (New England Biolabs, Ipswich, MA) respectively. Fluorescent nucleotides were incorporated by nick translation (Bionano Prep Labeling - NLRS Protocol). After repairing the nicks, DNA sample was electrophoresed into massively parallel nanochannels imaging. More than 325 Gb (from Nt. BspQI) and 266 Gb (from Nt. BssSI) image data were collected with a minimum molecule length of 150 kb respectively (Table 1).

Hi-C library was generated using DpnII restriction enzyme following *in situ* ligation protocols [20].

About 5 g young leaves were crosslinked with 1% formaldehyde for 10 minutes at room temperature, which was then quenched with a final concentration of 0.125 mol/L glycine. The crosslinked tissues were used for isolating intact nuclei according to the previously reported method [21]. The DpnII-digested chromatin was end-labeled with biotin-14-dATP (Thermo Fisher Scientific, Waltham, Massachusetts, USA) and used for *in-situ* DNA ligation. The DNA sample was extracted and purified，and then sheared using Covaris S2 (Covaris, Woburn, Massachusetts, USA). After A-tailing, pull-down and adapter ligation, the DNA library was sequenced on Illumina HiSeq X Ten System using a PE-150 module. As a result, more than 129 Gb (approximately 293×) clean data were generated after trimming low-quality reads and removing adaptors (Table 1).

**Transcriptome sequencing**

Total RNA of five tissues (hermaphrodite flower, staminate flower, leaf, shoot and young fruit) from 'WF18' was isolated using GeneJET Plant RNA Purification Mini Kit (Thermo Fisher Scientific, Waltham, Massachusetts, USA), and quantified by NanoDrop ND-2000 (Thermo Fisher Scientific, Waltham, Massachusetts, USA). RNA integrity was assessed using Agilent Bioanalyzer 2100 (Agilent Technologies, Santa Clara, California, USA). Those samples with integrity number greater than 8 was used for libraries construction with the dUTP method [22]. The RNA libraries with insert size around 350 bp were sequenced on Illimina HiSeq 4000 System using PE-150 module. Low-quality reads and adaptors were removed using Trimmomatic v. 0.38 [19]. As a result, more than 44.42 Gb clean data were generated (Table S1).

**Genome assembly by PacBio long reads**

The yellowhorn genome size was estimated based on k-mer spectrum using the kmergenie v. 1.7048 [23] and Illumina insert size of 450 bp library. The best k-mer was estimated to be 111 and genome size

was estimated to be 435.76 Mb with the diploid model (Figure S1). Karyotype analysis showed that 'WF18' was a diploid, and karyotype formula was 2n = 2X = 30 = 18m (2SAT) + 12 sm (Figure S2). The genomic contigs were assembled based on PacBio subreads using Falcon v. 0.7.0 [24]. Firstly, raw subreads were aligned to each other for error correction using Daligner v. 1.0 [25]. Then overlapped error-corrected reads were processed to generate consensus reads. To obtain a better assembly, length_cutoff 2,000, 3,000 and 5,000 were chosen respectively to filter raw reads in the first round for error correction. In the second round, length_cutoff_pr 5,000, 8,000 and 10,000 were chosen respectively for assembling overlapping step respectively to obtain consensus overlapping reads. The consensus overlapping reads were filtered with --max_diff 80 --max_cov 80 --min_cov 2 and used to construct string graphs. By finding single path of each contig graphs with optimal parameter (length_cutoff 2000 and length_cutoff_pr 8000), the draft genomic contigs were created to be 505.79 Mb in length with N50 values of 642,338 kb for 2,002 contigs (Table 3).

The draft genomic contigs were polished using PacBio long reads and Illumina paired-end reads. Firstly, the PacBio long reads were mapped to the genomic contigs using Pbalign v. 0.3.1. The self-polished consensus contigs were generated using Arrow algorithm v. 2.3.2. Secondly, the Illumina paired-end libraries of 280 and 450 bp were aligned to the self-polished consensus contigs with BWA v. 0.7.17 [26] and final polished contigs were obtained using pilon v. 1.22 [27]. The result showed that final polished genomic contigs were 508.45 Mb in length with N50 values of 645,453 kb (Table 3).

**Pseudo-chromosomes construction using 10X Genomics, BioNano optical maps and Hi-C**

The polished contigs were scaffolded with the 10X Genomics linked-reads by fragScaff v. 140324.1 [28]. By mapping the linked-reads to polished contigs with BWA, the alignments of each library were merged into a bamParse file, and filtered with parameter min N spacer size 3000, contig end node size

5000 and max contig end node size 10000. By scaffolding the merged banParse files with fragScaff, the scaffolded genome was assembled to be 513.92 Mb in length with N50 values of 2.33 Mb for 707 scaffolds (Table 3).

The 10X Genomics scaffolded was *in silico* digested with the nicking enzymes Nt.BspQI and Nt.BssSI, respectively. Scaffold genome of *in silico* maps and each BioNano Genomics maps were processed by Bionano Solve v. 3.1 (BioNano Genomics) to directly generate a hybrid scaffold. The result showed that 29 super-scaffolds were obtained in length of 461.66 Mb with N50 values of 29.98 Mb (Table 3).

The number of 7,192 (34.73 Mb) gaps distributed in hybrid super-scaffolds were filled with PacBio consensus long reads by PBJelly v. 15.2.20 [29], leading to 6,015 gaps were addressed. Subsequently, the gaps were filled with Illimina's insert size of 280 bp and 450 bp libraries paried-end reads by GMcloser v. 1.6.2 [30], giving rise to 77 gaps were closed. In total, 6,092 gaps were filled which reduced the N bases to 29.06 Mb represented 6.29% of hybrid super-scaffolds.

The gap-closed hybrid scaffolds were aligned to generate free duplicate Hi-C contacts based on *in situ* Hi-C data using Juicer pipeline v. 1.6.2 [31]. The free duplicate Hi-C contacts file (merged_nodups.txt) was used to scaffold splitting, anchor, order, orient, misjoin correction and revised by the 3D-DNA pipeline v. 180419 [32] to output "megascaffold" that concatenates all the pseudo-chromosomes. The megascaffold was then imported to the Juicebox Assembly Tools (JBAT) v. 1.8.8 [33] for manual review and refinement (Figure 3). The results showed that yellowhorn genome assembly was 439.97 Mb. Fifteen pseudo-chromosomes were assembled which covered 95.42% (419.84 Mb) of genome. The maximal length of these pseudo-chromosomes was 39.12 Mb and minimum one was 17.23 Mb (Figure 4, Table 3, and Table S2).

**Genome assemble assessment**

The completeness of genome assembly was assessed by searching against 1440 embryophyta specific single copy orthologs in genome assembly assessment mode using BUSCO v. 3.0.2 [34]. The result showed that 1,218 (84.58%) complete BUSCOs and 23 (1.60%) fragmented BUSCOs were identified in the yellowhorn genome (Table 4). A total of 85.10% *de novo* assembled transcripts were mapped to yellowhorn genome using BLAT v. 3.2.19 [35]. The genome assembly was also evaluated by QUAST v. 5.0.0 [36]. The result showed that NG50 (28.89 Mb) were closed to those of N50 (29.43 Mb) indicating that the assembly was in high quality (Table S3).

**Repeat sequence analysis**

For repetitive elements detection, RepeatMasker v. 4.07 was applied against RepBase plant repeat database (v. 23.06). *De novo* repetitive elements annotation was performed using RepeatModeler v. 1.0.11. As a result, the repetitive fractions represented 56.39% of the yellowhorn genome while repetitive elements and SSRs accounted for 54.81% and 1.58%, respectively. Therefore, the content of repeat fractions in yellowhorn genome was higher than that of other species of the Malvaceae, including *Citrus sinensis* (20.5%) [37], *Theobroma cacao* (25.7%) [38], *D. longan* (52.87%) [15], *Durio zibethinus* (54.8%) [39], but lower than that of *Gossypium raimondii* (57%) [40]. Moreover, LTR/*Copia* and LTR/*Gypsy* repeats were the most abundant repetitive elements, accounting for 11.91% and 11.68% of the genome, respectively (Table 5).

**Genome annotation**

To obtain long ORFs, spliced transcripts generated using Trinity v. 2.5.1 [41] with *de novo* and genome-guided model were aligned against the yellowhorn genome with PASA v. 2.3.3 [42]. The obtained ORFs were used for training *ab initio* predictors on repeat-masked genome. After *ab initio* gene prediction, with the trained optimal parameters, 20,980 genes from Augustus v. 3.2.2 [43], 28,134

genes from SNAP (version 2006-07-28) [44] and 32,205 genes from GeneMark-ES/ET v. 4.3.5 [45] were predicted. To predict homology genes, protein sequences of *C. sinensis*, *D. longan*, *T. cacao*, *Olea europaea*, *A. occidentale*, *Vitis vinifera*, *Glycine max*, *Populus tremula*, *Oryza sativa* and *Arabidopsis thaliana* were spliced-mapped to the repeat-masked yellowhorn genome using Exonerate v. 2.2.0 [46] with protein2genome model at 90% identity. Gene models from *ab initio* and homology predictions were combined to get a single high-confidence gene model by EVidenceModeler (EVM) v. 2.4.0 [47]. Weights were set according to the confidence of PASA Trinity set, Augustus gene set, Exonerate protein homology set, SNAP gene model set and GeneMark-ES/ET gene set in order. A total of 21,157 genes were obtained though EVM. After UTRs updating by running PASA on three rounds, 21,059 protein coding genes were obtained in the final gene models. Finally, by searching against the database of NR, UniProt, Pfam, GO, KEGG and CAZy, 18,503 (87.46%) gene models were functionally annotated with at least one term.

**Comparative phylogenomics**

The protein sequences of yellowhorn, together with *C. sinensis*, *D. longan*, *T. cacao*, *O. europaea*, *A. occidentale*, *V. vinifera*, *G. max*, *P. tremula*, *O. sativa*, *A. thaliana* were retrieved and filtered by removing redundancy of alternative spliced and shorter length proteins (less than 30 amino acids). The all-vs-all blast collections (with $1e^{-5}$) were used to ortholog by OrthoMCL v. 2.0.9 [48]. Orthogroups of 27,347 were constructed, followed by 9,905 species specific groups and 17,442 paralogs (Figure 5a, Table S4). Of them, 462 species specific groups containing 1,789 genes were further identified as yellowhorn specific groups. GO enrichment by topGO package v. 2.32.0 showed that “oxidation-reduction process”, “defense response”, “oxidoreductase activity” and “plastid inner membrane” were the most significantly enriched function categories (Table S5).

The protein sequences of 198 single copy orthogroups were performed to generate multiple sequence alignment using MAFFT v. 7.158b with an accurate option (L-INS-i) [49]. After each alignment merging, GBlocks v. 0.91b [50] was used to remove poorly aligned positions, divergent regions, and selected conserved blocks. Phylogeny was constructed using RAxMLv. 8.1.24 [51] with the evolutionary model GTR+GAMMA. A total of 1,000 rapid bootstrap inferences were performed. Divergent time of species was estimated using MCMCTree within PAML 4.9h package [52] with correlated rates clock and JC69 model settings. The Markov Chain Monte Carlo analysis was run on 20,000 generations with a burn-in of 2000 iterations. The evolutionary timescale of *O. sativa* and *A. thaliana* was obtained from TimeTree database and was used as calibrate point. The phylogenetic tree visualized in FigTree v. 1.4.3 [53] suggested that yellowhorn diverged from the common ancestral of *D. longan* at approximately 58.63 million years ago (Figure 5b).

**Transcriptome analysis of tissue-specific expression**

RNA sequencing paired reads of five tissues including hermaphrodite flower, staminate flower, leaf, shoot and young fruit were aligned to genome using Tophat v. 2.1.2 [54] with mean genome mapping ratio of 75.68%. The transcripts were assembled using StringTie v. 1.3.4d [55]. The abundance of gene expression was estimated using the 'scaledTPM' method in txImport v. 1.8.0 [56]. The results showed that 341, 113, 100, 135 and 125 genes were specifically expressed in leaf, hermaphrodite flower, shoot, staminate flower and young fruit, respectively (Fig. 6a, Table S6). GO enrichment of hermaphrodite flower-specific genes showed that the function of “oxidation-reduction process”, “defense response”, “monooxygenase activity”, “oxidoreductase activity” and “membrane part” were most significantly enriched. “Growth related” and “membrane part” were significantly enriched functions in leaf. For shoot-specific genes, “response to stress”, “regulation of developmental process” and “extracellular

region” were significantly enriched. The GO terms of “Function of negative regulation of flower development and reproductive process”, “oxidoreductase activity”and “membrane” were mostly enriched in staminate flower. Additionally, GO enrichment of young fruit-specific genes showed that “metabolic process”, “binding” and “lyase activity” were the mostly enriched functions (Fig. 6, Table S7).

## Discussion

In the present study, a high-quality draft genome of yellowhorn was sequenced and assembled by firstly integrating Illumina sequencing, PacBio single-molecule real-time sequencing, 10X Genomics link-reads, Bionano optical maps and Hi-C sequencing data. Yellowhorn genome contained higher amount of repetitive elements than the other reported species of the Malvaceae family. Moreover, LTR/*Gypsy* (11.68%) and LTR/*Copia* (11.91%) repeats in yellowhorn genome appeared to have expanded as compared to *T. cacao* (9% LTR/*Gypsy* and 7% LTR/*Copia*) [38]. It’s different from the patterns observed in *D. longan*, *D. zibethinus*, *G. raimondii*, which appear to have LTR/*Gypsy* expanded and LTR/*Copia* contracted as compared to *T. cacao* [15, 39, 40]. Similar to its closely related species *D. longan*, a large number of LTR retrotransposons (23.90% of the genome) were identified in yellowhorn (Table 5). A new Xanthoceraceae family was published to alteration of family limits for Sapindaceae (Sapindales) by Buerki *et al*. [13], but APG IV (2016) did not alter circumscription of Sapindaceae [2]. The result of comparative phylogenomics suggested that yellowhorn diverged from the common ancestral of *D. longan* within Sapindaceae (Figure 6).

To explore the tissue-specific genes, we performed transcriptomic analysis of five yellowhorn tissues including hermaphrodite flower, staminate flower, leaf, shoot and young fruit. A total of 814 tissue-specific genes including 45 transcription factors were obtained. The largest number of

tissues-specific genes was found in hermaphrodite flower tissue, such as flowering-promoting factor 1-like protein 1 (FPF1, XS13G06782) and DIVARICATA (DIV, XS07G17645). FPF1 was a flower-specific gene that positively regulate flowering in *Arabidopsis* [57]. Its homolog gene enhances adventitious root formation in *O. sativa* [58]. DIV, an MYB family of transcription factor, has been found to control the asymmetry of the corolla or petals in *Antirrhinum* [59]. For leaf-specific genes, the TCP family transcription factor 12 (TCP12, XS06G15731) has been reported to regulate leaf development and play an essential role in determining leaf size and shape [60, 61]. Further investigation is required for understanding the function of these specific genes and their regulation.

## Figure and Table legend

Figure 1. Morphological characteristic of yellowhorn superior 'WF18'. (A) Raceme and shoot. (B) Hermaphrodite flower at 1 DPA (days post flower), 3DPA, 5DPA. (C) Capsular fruits. (D) Seeds and kernel.

Figure 2. Flowchart of genome assembly and annotation.

Figure 3. Contact maps of Hi-C links among chromosomes. Blue square represents draft scaffold. Green square represents pseudo-chromosome. The color bar illuminated the Hi-C contact density in the plot.

Figure 4. Yellowhorn genome features. The chromosomes size in Mb scale. The denotation of the distribution of gene density, repeat density and GC density are listed on the top right corner. The syntenic blocks were represented by curves in the center of the graph.

Figure 5. Phylogenomics analysis of yellowhorn genome. (A) Orthologue clustering analysis of the protein-coding genes in yellowhorn genome and ten other species. (B) Phylogenetic tree and divergence time of yellowhorn and ten other species. The numbers beside the branching nodes are the

predicted divergence time.

Figure 6. Tissue-specific gene analysis. (A) Venn diagram showing shared and unique genes among five tissues. Numbers represent the number of genes in unique or shared. (B-D) GO enrichment of tissue-specific genes. The node size represents the gene numbers enriched in each GO category. The color bar illuminates p-value from red (low) to blue (high) in B, C, and D.

Table 1. Statistics of Illumina, 10X Genomics, BioNano and Hi-C sequencing data.

Table 2. Statistics of PacBio Sequal sequencing data.

Table 3. Summary of yellowhorn genome assembly.

Table 4. BUSCO assessment of yellowgorn genome.

Table 5. Repeat content of yellowhorn genome.

**Additional files**

Table S1. Statistics of transcriptome sequencing data.

Table S2. The features of yellowhorn genome.

Table S3. Genome QC report of yellowhorn by QUAST.

Table S4. Orthogroups of the protein-coding genes in yellowhorn genome and other species.

Table S5. GO enrichment of yellowhorn specific genes.

Table S6. Yellowhorn tissue-specific genes.

Table S7. GO enrichment of yellowhorn tissue-specific genes.

Figure S1. Estimation of genome size. (A) K-mer plot. The x axis is k-mer size and the y axis is a number of k-mers. (B) Histogram and fit for k-mer 111. Red is the fit of the complete statistical model of the histogram (erronous k-mers + genomic k-mers). Blue represent the heterozygous k-mers. Green represents the homozygous k-mers.

Figure S2. Karyogram of yellowhorn superior 'WF18'. (A) Chromosome at diakinesis of pollen mother cell meiophase. Bar = 5μm. (B) Yellowhorn superior 'WF18' was a diploid plant, 2n = 2X = 30. (C) Ideogram (Karyotype formula of yellowhorn superior 'WF18' was 2n = 2X = 30 = 18m (2SAT) +12 sm).

**Funding**

This work was financially supported by the Improved Variety Program of Shandong Province of China (2016LZGC013), the Innovative Project of Forestry Science and Technology of Shandong Province of China (LYCX05-2018-26) and the Funds of Shandong 'Double Tops' Program (SYL2017XTTD09).

**Abbreviations**

BUSCO: Benchmarking Universal Single-Copy Orthologs; Hi-C: High-through Chromosome conformation capture; QUAST: Quality Assessment Tool for Genome Assemblies; SSRs: simple sequence repeats; LINEs: long interspersed nuclear elements; PASA: Program to Assemble Spliced Alignments; ORFs: open reading frames; UTR: Untranslated Region; GO: GeneOntology; KEGG: Kyoto Encyclopedia of Genes and Genomes; CAZy: Carbohydrate-Active enZYmes; LTR: long terminal repeat

**Availability of supporting data**

Bioproject：PRJNA496350

Biosample：SAMN10239523

**Software and Reference data**

| Software | URLs |
|---|---|
| FASTQC | http://www.bioinformatics.babraham.ac.uk/projects/fastqc/ |
| Trimmomatic | http://www.usadellab.org/cms/index.php?page=trimmomatic |

| | |
|---|---|
| KmerGenie | http://kmergenie.bx.psu.edu/ |
| FALCON | https://github.com/PacificBiosciences/FALCON |
| Pbalign | https://github.com/PacificBiosciences/pbalign |
| Arrow | https://github.com/PacificBiosciences/GenomicConsensus |
| BWA | http://bio-bwa.sourceforge.net/ |
| fragScaff | https://sourceforge.net/projects/fragscaff/ |
| Bionano Solve | https://bionanogenomics.com/support-page/bionano-solve/ |
| PBJelly | https://sourceforge.net/projects/pb-jelly/files/latest/download |
| GMcloser | https://sourceforge.net/projects/gmcloser/ |
| Juicer | https://github.com/aidenlab/juicer |
| BUSCO | https://busco.ezlab.org/ |
| QUAST | http://quast.bioinf.spbau.ru/ |
| RepeatMasker | http://repeatmasker.org/ |
| RepeatModeler | http://www.repeatmasker.org/RepeatModeler/ |
| Trinity | https://github.com/trinityrnaseq/trinityrnaseq |
| PASA | https://github.com/PASApipeline/PASApipeline |
| Augustus | http://bioinf.uni-greifswald.de/augustus/ |
| SNAP | https://github.com/KorfLab/SNAP |
| GeneMark-ES/ET | http://exon.gatech.edu/GeneMark/ |
| Exonerate | https://www.ebi.ac.uk/about/vertebrate-genomics/software/exonerate |
| EVidenceModeler | http://evidencemodeler.github.io/ |
| OrthoMCL | http://orthomcl.org/orthomcl/ |

| | |
|---|---|
| topGO | http://bioconductor.org/packages/topGO/ |
| MAFFT | https://mafft.cbrc.jp/alignment/software/ |
| RaxML | http://evomics.org/learning/phylogenetics/raxml/ |
| PAML | http://abacus.gene.ucl.ac.uk/software/paml.html |
| Tophat | http://ccb.jhu.edu/software/tophat/index.shtml |
| **Reference data** | **URLs** |
| RepBase plant repeat database | https://www.girinst.org/server/RepBase/ |
| TimeTree database | http://timetree.org/ |
| *O. europaea* | http://olivegenome.org/ |
| *C. sinensis* | http://citrus.hzau.edu.cn/orange/ |
| *G. max* | ftp://ftp.jgi-psf.org/pub/compgen/phytozome/v9.0/Gmax/ |
| *A. thaliana* | https://www.arabidopsis.org/ |
| *O. sativa* | http://rapdb.dna.affrc.go.jp/ |
| *P. trichocarpa* | https://genome.jgi.doe.gov/ |
| *V. vinifera* | http://genomes.cribi.unipd.it/grape/ |
| *D. longan* | ftp://penguin.genomics.cn/pub/10.5524/100001_101000/100276/ |
| *A. occidentale* | https://genome.jgi.doe.gov/ |
| *T. cacao* | http://cocoa-genome-hub.southgreen.fr/ |

**Authors' contributions**

KY conceived this genome project and coordinated research activities; LY, JL, KY, YL and NW designed the experiments; LY, JL, HL, Qiang L and XH assembled and annotated the genome; HL, Qingyun L, RZ and XH analyzed transcriptome and phylogenies; Qiang L, SL, FY, and QD collected

and maintained plant materials; JS estimated genome size and analyzed karyotype. JL, LY, KY, Qiang L, HL, YL and NW wrote the manuscript. All authors have read and approved the final manuscript.

**Competing interests**

The authors declare that they have no competing interests.

**Author details**

1 College of Forestry, Shandong Agricultural University, Tai'an 271018, China. 2 College of Plant Protection, Shandong Agricultural University, Tai'an 271018, China. 3 Worth Agricultural Development Co. Ltd., Weifang 262100, China. 4 KeGene Science ＆ Technology Co. Ltd., Tai'an 271018, China

**Reference**

1. Nianhe X and Gadek PA. Sapindaceae. In: Wu Z, Raven PH and Hong D, editors. Flora of China: Hippocastanaceae through Theaceae. Beijing, China: Science Press; 2007. p. 5-24.

2. Chase MW, Christenhusz M, Fay M, Byng J, Judd W, Soltis D, et al. An update of the Angiosperm Phylogeny Group classification for the orders and families of flowering plants: APG IV. Botanical Journal of the Linnean Society. 2016;181 1:1-20.

3. Wang Q, Yang L, Ranjitkar S, Wang J, Wang X, Zhang D, et al. Distribution and in situ conservation of a relic Chinese oil woody species *Xanthoceras sorbifolium* (yellowhorn). Canadian Journal of Forest Research. 2017;47 11:1450-6.

4. Wang Q, Zhu R, Cheng J, Deng Z, Guan W and Elkassaby YA. Species association in *Xanthoceras sorbifolium* Bunge communities and selection for agroforestry establishment. Agroforestry Systems. 2018:1-13.

5. Venegascaleron M, Ruizmendez MV, Martinezforce E, Garces R and Salas JJ. Characterization of

*Xanthoceras sorbifolium* Bunge seeds: Lipids, proteins and saponins content. Industrial Crops and Products. 2017;109:192-8.

6. Yao Z-Y, Qi J-H and Yin L-M. Biodiesel production from *Xanthoceras sorbifolia* in China: Opportunities and challenges. Renewable and Sustainable Energy Reviews. 2013;24:57-65. doi:10.1016/j.rser.2013.03.047.

7. Yu H, Fan S, Bi Q, Wang S, Hu X, Chen M, et al. Seed morphology, oil content and fatty acid composition variability assessment in yellow horn ( *Xanthoceras sorbifolium* Bunge) germplasm for optimum biodiesel production. Industrial Crops and Products. 2017;97:425-30. doi:10.1016/j.indcrop.2016.12.054.

8. Xiao W, Wang Y, Zhang P, Li N, Jiang S, Wang JH, et al. Bioactive barrigenol type triterpenoids from the leaves of *Xanthoceras sorbifolia* Bunge. European Journal of Medicinal Chemistry. 2013;60:263-70. doi:10.1016/j.ejmech.2012.12.022.

9. Yu L, Wang X, Wei X, Wang M, Chen L, Cao S, et al. Triterpenoid saponins from *Xanthoceras sorbifolia* Bunge and their inhibitory activity on human cancer cell lines. Bioorganic & Medicinal Chemistry Letters. 2012;22 16:5232-8. doi:10.1016/j.bmcl.2012.06.061.

10. Wang D, Su D, Yu B, Chen C, Cheng L, Li X, et al. Novel anti-tumour barringenol-like triterpenoids from the husks of *Xanthoceras sorbifolia* Bunge and their three dimensional quantitative structure activity relationships analysis. Fitoterapia. 2017;116:51-60. doi:10.1016/j.fitote.2016.11.002.

11. Wang D, Su D, Li X-Z, Liu D, Xi R-G, Gao H-Y, et al. Barrigenol triterpenes from the husks of *Xanthoceras sorbifolia* Bunge and their antitumor activities. RSC Advances. 2016;6 33:27434-46. doi:10.1039/c6ra02706g.

12. Li Y, Xu J, Xu P, Song S, Liu P, Chi T, et al. *Xanthoceras sorbifolia* extracts ameliorate dendritic spine deficiency and cognitive decline via upregulation of BDNF expression in a rat model of Alzheimer's disease. Neuroscience Letters. 2016;629:208-14. doi:10.1016/j.neulet.2016.07.011.

13. Buerki S. Phylogeny and circumscription of Sapindaceae revisited: molecular sequence data, morphology and biogeography support recognition of a new family, Xanthoceraceae. Plant Ecology and Evolution. 2010;143 2:148-59. doi:10.5091/plecevo.2010.437.

14. Buerki S, Lowry PP, Phillipson PB and Callmander MW. Molecular Phylogenetic and Morphological Evidence Supports Recognition of Gereaua, a New Endemic Genus of Sapindaceae from Madagascar. Systematic Botany. 2010;35 1:172-80.

15. Lin Y, Min J, Lai R, Wu Z, Chen Y, Yu L, et al. Genome-wide sequencing of longan *(Dimocarpus longan* Lour.) provides insights into molecular basis of its polyphenol-rich characteristics. Gigascience. 2017;6 5:1-14. doi:10.1093/gigascience/gix023.

16. Chen S and Zhang X. Characterization of the complete chloroplast genome of *Xanthoceras sorbifolium*, an endangered oil tree. Conservation Genetics Resources. 2017;9 4:1-4.

17. Liu Y, Huang Z, Ao Y, Li W and Zhang Z. Transcriptome analysis of yellow horn (*Xanthoceras sorbifolia* Bunge): a potential oil-rich seed tree for biodiesel in China. PLoS One. 2013;8 9:e74441. doi:10.1371/journal.pone.0074441.

18. Zhou Q and Zheng Y. Comparative De Novo Transcriptome Analysis of Fertilized Ovules in *Xanthoceras sorbifolium* Uncovered a Pool of Genes Expressed Specifically or Preferentially in the Selfed Ovule That Are Potentially Involved in Late-Acting Self-Incompatibility. PLoS One. 2015;10 10:e0140507. doi:10.1371/journal.pone.0140507.

19. Bolger AM, Lohse M and Usadel B. Trimmomatic: a flexible trimmer for Illumina sequence data. Bioinformatics. 2014;30 15:2114-20. doi:10.1093/bioinformatics/btu170.

20. Belaghzal H, Dekker J and Gibcus JH. Hi-C 2.0: An optimized Hi-C procedure for high-resolution genome-wide mapping of chromosome conformation. Methods. 2017;123:56-65. doi:10.1016/j.ymeth.2017.04.004.

21. Sikorskaite S, Rajamaki ML, Baniulis D, Stanys V and Valkonen JP. Protocol: Optimised methodology for isolation of nuclei from leaves of species in the Solanaceae and Rosaceae families. Plant Methods. 2013;9:31. doi:10.1186/1746-4811-9-31.

22. Parkhomchuk D, Borodina T, Amstislavskiy V, Banaru M, Hallen L, Krobitsch S, et al. Transcriptome analysis by strand-specific sequencing of complementary DNA. Nucleic Acids Research. 2009;37 18:e123. doi:10.1093/nar/gkp596.

23. Chikhi R and Medvedev P. Informed and automated k-mer size selection for genome assembly. Bioinformatics. 2014;30 1:31-7. doi:10.1093/bioinformatics/btt310.

24. Pendleton M, Sebra R, Pang AW, Ummat A, Franzen O, Rausch T, et al. Assembly and diploid architecture of an individual human genome via single-molecule technologies. Nature Methods. 2015;12 8:780-6. doi:10.1038/nmeth.3454.

25. Myers G. Efficient Local Alignment Discovery amongst Noisy Long Reads. workshop on algorithms in bioinformatics. 2014:52-67.

26. Li H and Durbin R. Fast and accurate short read alignment with Burrows-Wheeler transform. Bioinformatics. 2009;25 14:1754-60. doi:10.1093/bioinformatics/btp324.

27. Walker BJ, Abeel T, Shea T, Priest M, Abouelliel A, Sakthikumar S, et al. Pilon: an integrated tool for comprehensive microbial variant detection and genome assembly improvement. PLoS One.

2014;9 11:e112963. doi:10.1371/journal.pone.0112963.

28. Adey A, Kitzman JO, Burton JN, Daza R, Kumar A, Christiansen L, et al. In vitro, long-range sequence information for de novo genome assembly via transposase contiguity. Genome Research. 2014;24 12:2041-9. doi:10.1101/gr.178319.114.

29. English AC, Richards S, Han Y, Wang M, Vee V, Qu J, et al. Mind the gap: upgrading genomes with Pacific Biosciences RS long-read sequencing technology. PLoS One. 2012;7 11:e47768. doi:10.1371/journal.pone.0047768.

30. Kosugi S, Hirakawa H and Tabata S. GMcloser: closing gaps in assemblies accurately with a likelihood-based selection of contig or long-read alignments. Bioinformatics. 2015;31 23:3733-41. doi:10.1093/bioinformatics/btv465.

31. Durand NC, Shamim MS, Machol I, Rao SS, Huntley MH, Lander ES, et al. Juicer Provides a One-Click System for Analyzing Loop-Resolution Hi-C Experiments. Cell Systems. 2016;3 1:95-8. doi:10.1016/j.cels.2016.07.002.

32. Dudchenko O, Batra SS, Omer AD, Nyquist SK, Hoeger M, Durand NC, et al. De novo assembly of the *Aedes aegypti* genome using Hi-C yields chromosome-length scaffolds. Science. 2017;356 6333:92-5. doi:10.1126/science.aal3327.

33. Durand NC, Robinson JT, Shamim MS, Machol I, Mesirov JP, Lander ES, et al. Juicebox Provides a Visualization System for Hi-C Contact Maps with Unlimited Zoom. Cell Systems. 2016;3 1:99-101. doi:10.1016/j.cels.2015.07.012.

34. Simao FA, Waterhouse RM, Ioannidis P, Kriventseva EV and Zdobnov EM. BUSCO: assessing genome assembly and annotation completeness with single-copy orthologs. Bioinformatics. 2015;31 19:3210-2. doi:10.1093/bioinformatics/btv351.

35. Kent WJ. BLAT--the BLAST-like alignment tool. Genome Research. 2002;12 4:656-64. doi:10.1101/gr.229202.

36. Gurevich A, Saveliev V, Vyahhi N and Tesler G. QUAST: quality assessment tool for genome assemblies. Bioinformatics. 2013;29 8:1072-5. doi:10.1093/bioinformatics/btt086.

37. Xu Q, Chen LL, Ruan X, Chen D, Zhu A, Chen C, et al. The draft genome of sweet orange (*Citrus sinensis*). Nature Genetics. 2013;45 1:59-66. doi:10.1038/ng.2472.

38. Argout X, Salse J, Aury JM, Guiltinan MJ, Droc G, Gouzy J, et al. The genome of *Theobroma cacao*. Nature Genetics. 2011;43 2:101-8. doi:10.1038/ng.736.

39. Teh BT, Lim K, Yong CH, Ng CCY, Rao SR, Rajasegaran V, et al. The draft genome of tropical fruit durian (*Durio zibethinus*). Nature Genetics. 2017;49 11:1633-41. doi:10.1038/ng.3972.

40. Wang K, Wang Z, Li F, Ye W, Wang J, Song G, et al. The draft genome of a diploid cotton *Gossypium raimondii*. Nature Genetics. 2012;44 10:1098-103. doi:10.1038/ng.2371.

41. Grabherr MG, Haas BJ, Yassour M, Levin JZ, Thompson DA, Amit I, et al. Full-length transcriptome assembly from RNA-Seq data without a reference genome. Nature Biotechnology. 2011;29 7:644-52. doi:10.1038/nbt.1883.

42. Haas BJ, Delcher AL, Mount SM, Wortman JR, Smith RK, Jr., Hannick LI, et al. Improving the *Arabidopsis* genome annotation using maximal transcript alignment assemblies. Nucleic Acids Research. 2003;31 19:5654-66.

43. Stanke M and Waack S. Gene prediction with a hidden Markov model and a new intron submodel. Bioinformatics. 2003;19 Suppl 2:ii215-25.

44. Korf I. Gene finding in novel genomes. BMC Bioinformatics. 2004;5:59. doi:10.1186/1471-2105-5-59.

45. Lomsadze A, Ter-Hovhannisyan V, Chernoff YO and Borodovsky M. Gene identification in novel eukaryotic genomes by self-training algorithm. Nucleic Acids Research. 2005;33 20:6494-506. doi:10.1093/nar/gki937.

46. Slater GS and Birney E. Automated generation of heuristics for biological sequence comparison. BMC Bioinformatics. 2005;6:31. doi:10.1186/1471-2105-6-31.

47. Haas BJ, Salzberg SL, Zhu W, Pertea M, Allen JE, Orvis J, et al. Automated eukaryotic gene structure annotation using EVidenceModeler and the Program to Assemble Spliced Alignments. Genome Biology. 2008;9 1:R7. doi:10.1186/gb-2008-9-1-r7.

48. Li L, Stoeckert CJ, Jr. and Roos DS. OrthoMCL: identification of ortholog groups for eukaryotic genomes. Genome Research. 2003;13 9:2178-89. doi:10.1101/gr.1224503.

49. Katoh K and Standley DM. MAFFT multiple sequence alignment software version 7: improvements in performance and usability. Molecular Biology and Evolution. 2013;30 4:772-80. doi:10.1093/molbev/mst010.

50. Talavera G and Castresana J. Improvement of phylogenies after removing divergent and ambiguously aligned blocks from protein sequence alignments. Systematic Biology. 2007;56 4:564-77. doi:10.1080/10635150701472164.

51. Stamatakis A. RAxML version 8: a tool for phylogenetic analysis and post-analysis of large phylogenies. Bioinformatics. 2014;30 9:1312-3. doi:10.1093/bioinformatics/btu033.

52. Yang Z. PAML: a program package for phylogenetic analysis by maximum likelihood. Comput Appl Biosci. 1997;13 5:555-6.

53. Drummond AJ and Rambaut A. BEAST: Bayesian evolutionary analysis by sampling trees. BMC Evolutionary Biology. 2007;7:214. doi:10.1186/1471-2148-7-214.

54. Kim D, Pertea G, Trapnell C, Pimentel H, Kelley R and Salzberg SL. TopHat2: accurate alignment of transcriptomes in the presence of insertions, deletions and gene fusions. Genome Biology. 2013;14 4:R36. doi:10.1186/gb-2013-14-4-r36.

55. Pertea M, Pertea GM, Antonescu CM, Chang TC, Mendell JT and Salzberg SL. StringTie enables improved reconstruction of a transcriptome from RNA-seq reads. Nature Biotechnology. 2015;33 3:290-5. doi:10.1038/nbt.3122.

56. Soneson C, Love MI and Robinson MD. Differential analyses for RNA-seq: transcript-level estimates improve gene-level inferences. F1000Reseach. 2015;4:1521. doi:10.12688/f1000research.7563.2.

57. Duan L, Dietrich D, Ng CH, Chan PM, Bhalerao R, Bennett MJ, et al. Endodermal ABA signaling promotes lateral root quiescence during salt stress in *Arabidopsis* seedlings. Plant Cell. 2013;25 1:324-41. doi:10.1105/tpc.112.107227.

58. Xu ML, Jiang JF, Ge L, Xu YY, Chen H, Zhao Y, et al. FPF1 transgene leads to altered flowering time and root development in rice. Plant Cell Reports. 2005;24 2:79-85. doi:10.1007/s00299-004-0906-8.

59. Galego L and Almeida J. Role of DIVARICATA in the control of dorsoventral asymmetry in *Antirrhinum* flowers. Genes & Development. 2002;16 7:880-91. doi:10.1101/gad.221002.

60. Tao Q, Guo D, Wei B, Zhang F, Pang C, Jiang H, et al. The TIE1 transcriptional repressor links TCP transcription factors with TOPLESS/TOPLESS-RELATED corepressors and modulates leaf development in *Arabidopsis*. Plant Cell. 2013;25 2:421-37. doi:10.1105/tpc.113.109223.

61. Nath U, Crawford BC, Carpenter R and Coen E. Genetic control of surface curvature. Science. 2003;299 5611:1404-7. doi:10.1126/science.1079354.

Table 1. Statistics of Illumina, 10X Genomics, BioNano and Hi-C sequencing data

| Platform | Library type | Insert size (bp) | Read length (bp) | No. of reads (bp) | Total base (bp) | Reads retained after trimming |
|---|---|---|---|---|---|---|
| Illumina | 280 bp Size | 280 | 150 | 451,336,880 | 67,700,532,000 | 439,835,802 |
| | 450 bp Size | 450 | 150 | 696,876,778 | 104,531,516,700 | 658,715,183 |
| 10x Genomics | 350 bp Size | 350 | 150 | 228,702,484 | 68,610,745,200 | 228,702,484 |
| Hi-C | 600 bp Size | 600 | 150 | 932,763,228 | 140,847,247,428 | 891,717,408 |
| BioNano | Nt. BspQI* | | 150,000 | | 325,238,200,000 | |
| | Nb. BssSI* | | 150,000 | | 266,976,000,000 | |

*Enzyme

 Click here to download Table Table 2.docx

Table 2. Statistics of PacBio Sequal sequencing data

| Index | PacBio |
|---|---|
| Total Number of reads | 7,062,244 |
| Mean length of raw reads (bp) | 226,712 |
| N50 of raw reads (bp) | 374,500 |
| Mean length of subreads (bp) | 156,717 |
| N50 of subreads (bp) | 237,539 |
| Coverage (X)* | 159.57 |

*Coverage (X) = (read count * read length) / estimated genome size.

 Click here to download Table Table 3.docx

Table 3. Summary of yellowhorn genome assembly.

| Statistics | PacBio | | 10X Genomics | BioNano | Hi-C | |
|---|---|---|---|---|---|---|
| | Contig | Polished Contig | | | Scaffold | Chromosome |
| Total number | 2,002 | 2,002 | 707 | 29 | 267 | 15 |
| Total length (bp) | 505,787,109 | 508,445,799 | 513,924,146 | 461,662,473 | 439,965,977 | 419,835,445 |
| N50 length (bp) | 642,338 | 645,453 | 2,334,658 | 29,979,918 | 29,432,808 | 29,432,808 |
| N90 length (bp) | 113,799 | 114,103 | 492,748 | 15,941,042 | 17,893,618 | 17,893,618 |
| Max length (bp) | 4,375,484 | 4,395,303 | 21,312,255 | 75,772,594 | 39,123,600 | 39,123,600 |
| GC content (%) | 35.25 | 35.13 | 34.67 | 32.39 | 32.76 | 34.18 |

Table 4. BUSCO assessment of yellowgorn genome.

| Description | | yellowhorn | |
|---|---|---|---|
| | | Number | Percentage (%) |
| Complete BUSCOs (C) | Complete and single-copy BUSCOs (S) | 1,175 | 81.60 |
| | Complete and duplicated BUSCOs (D) | 43 | 2.98 |
| Fragmented BUSCOs (F) | | 23 | 1.60 |
| Missing BUSCOs (M) | | 199 | 13.82 |
| Total BUSCO groups | | 1,440 | 100 |

Table 5. Repeat content of yellowhorn genome Click here to download Table Table 5.docx

Table 5. Repeat content of yellowhorn genome.

| Term | | Length (bp) | Percentage of genome (%) |
|---|---|---|---|
| DNAs* | DNA | 374,909 | 0.09 |
| | DNA/CMC-EnSpm | 1,699,637 | 0.39 |
| | DNA/MuLE-MuDR | 3,896,024 | 0.89 |
| | DNA/PIF-Harbinger | 1,104,979 | 0.25 |
| | DNA/TcMar-Pogo | 94,067 | 0.02 |
| | DNA/hAT-Ac | 4,103,980 | 0.93 |
| | DNA/hAT-Tag1 | 890,950 | 0.20 |
| | DNA/hAT-Tip100 | 1,213,576 | 0.28 |
| SINEs* | SINE | 343 | 0.00 |
| | SINE/tRNA | 10,674 | 0.00 |
| LINE* | LINE/L1 | 16,861,661 | 3.83 |
| LTRs* | LTR | 2,861 | 0.00 |
| | LTR/Caulimovirus | 1,360,538 | 0.31 |
| | LTR/Copia | 52,384,264 | 11.91 |
| | LTR/Gypsy | 51,370,228 | 11.68 |
| | LTR/Pao | 88 | 0.00 |
| Low_complexity | | 1,516,978 | 0.34 |
| RC* | | 4,215 | 0.00 |
| RC/Helitron | | 5,949 | 0.00 |
| rRNA | | 64,618 | 0.01 |
| Simple_repeat | | 6,971,711 | 1.58 |
| Unknown | | 104,792,508 | 23.76 |
| Total | | 248,724,758 | 56.39 |
| Genome size | | 439,965,977 | 100.00 |

*DNA: DNA transposons; LINE: long interspersed nuclear elements; SINE: short interspersed nuclear elements; LTR: long terminal repeat； RC: rolling circle replication.

Figure 1. Morphological characteristic of yellowhorn superior 'WF18'

Click here to download Figure Figure 1.tif

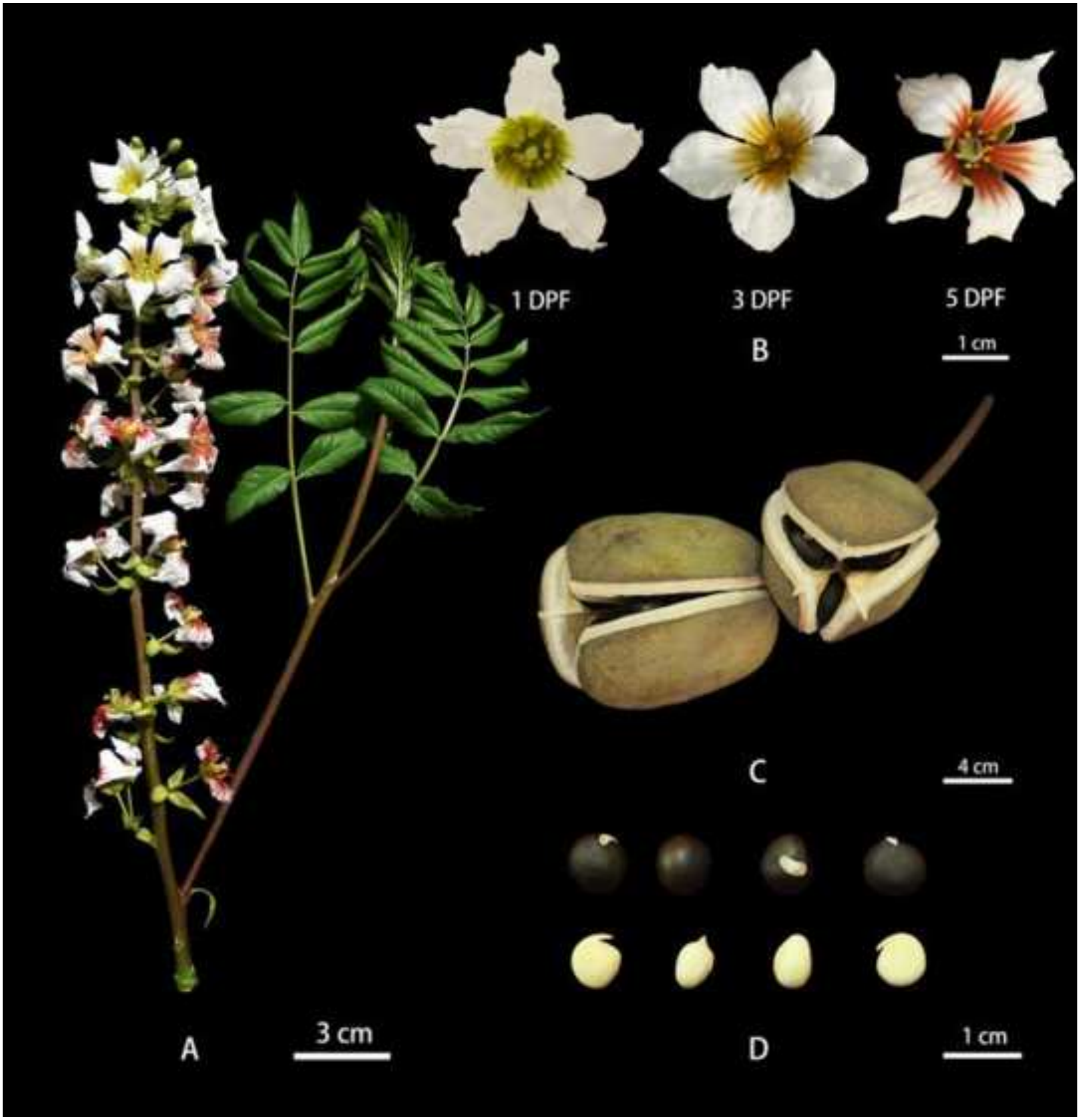

Figure 2. Flowchart of genome assembly and annotation

Click here to download Figure Figure 2.tif

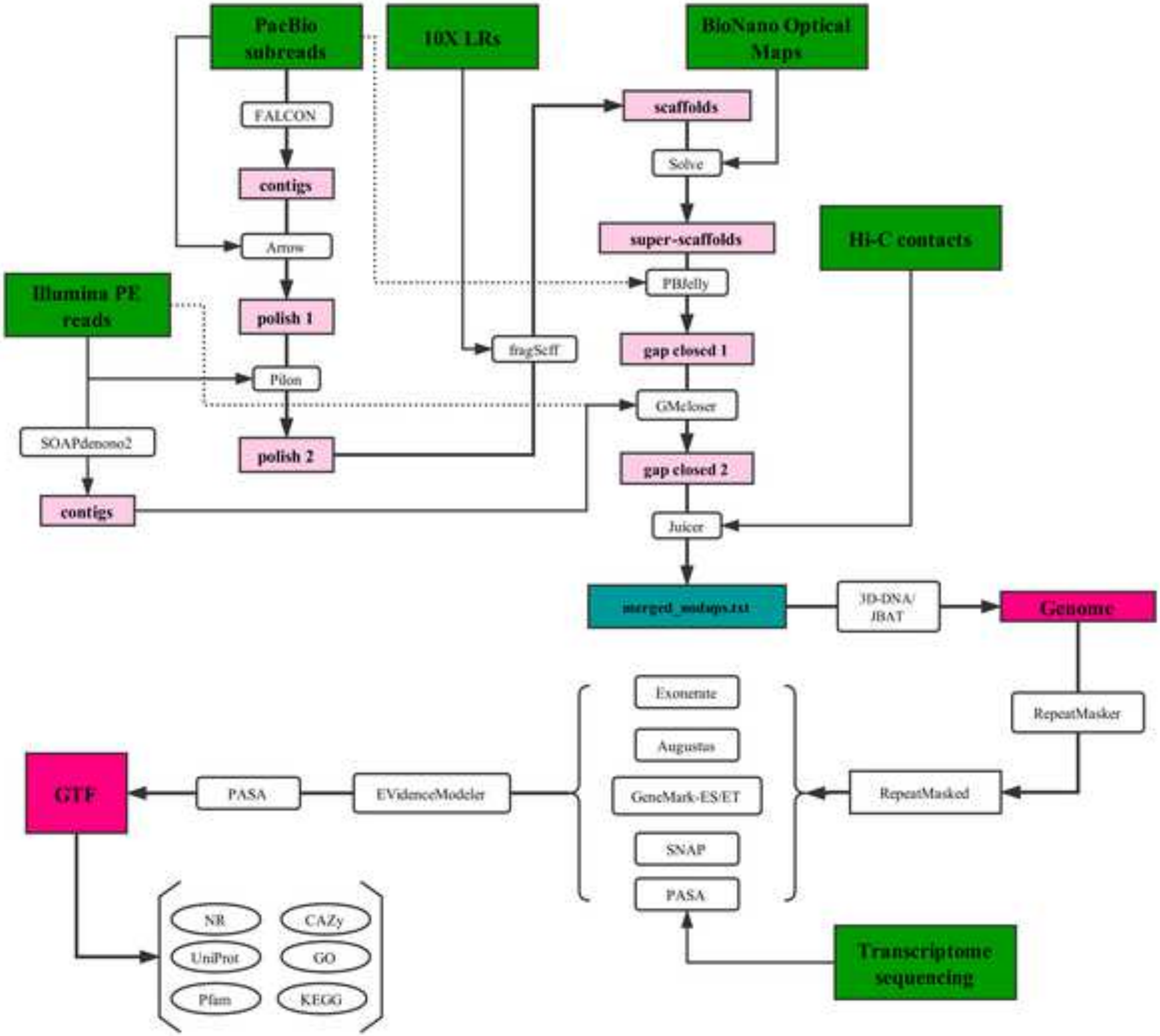

Figure 3. Contact maps of Hi-C links among chromosomes

Click here to download Figure Figure 3.png

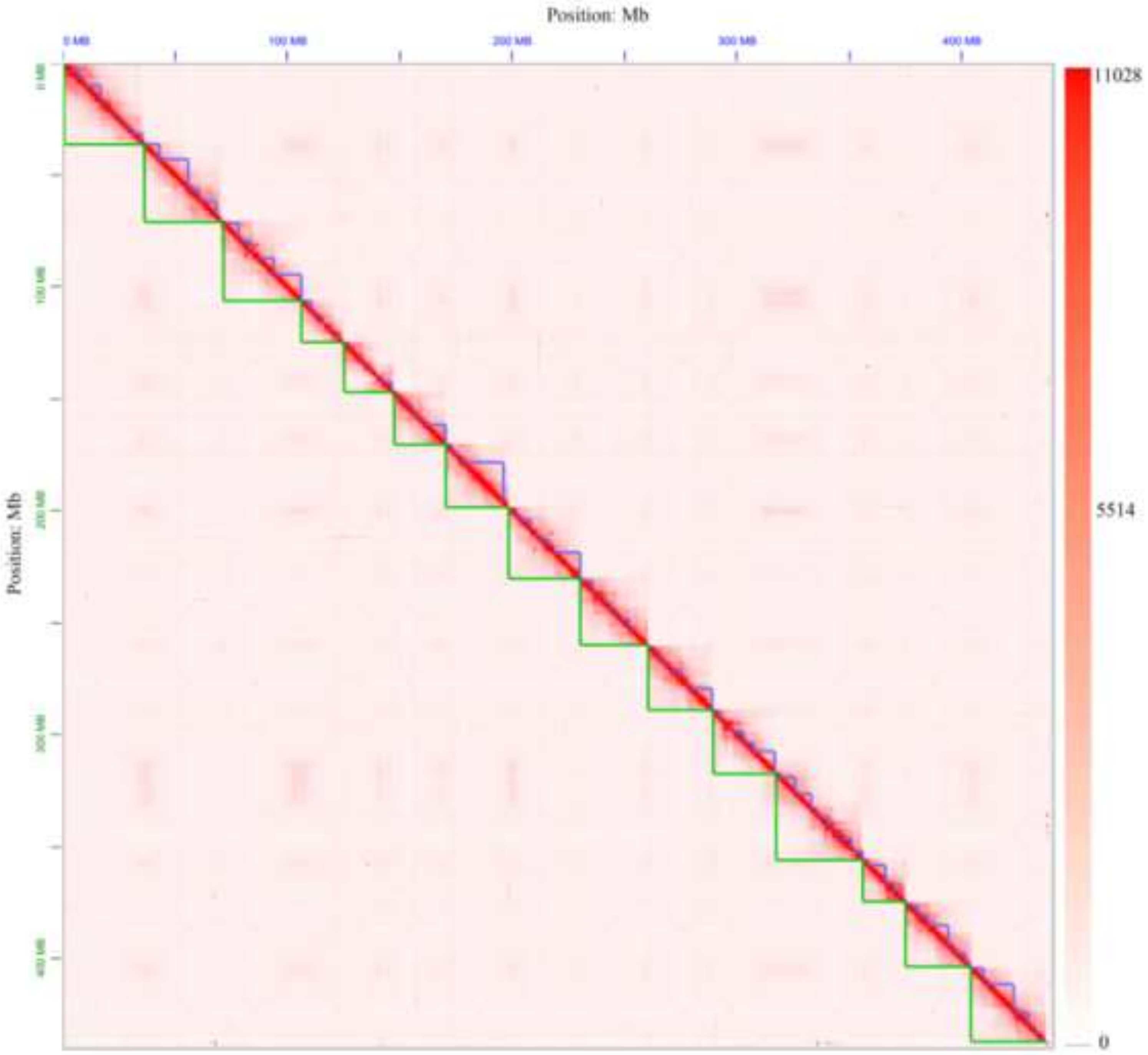

Figure 4. Yellowhorn genome features

Click here to download Figure Figure 4.tif

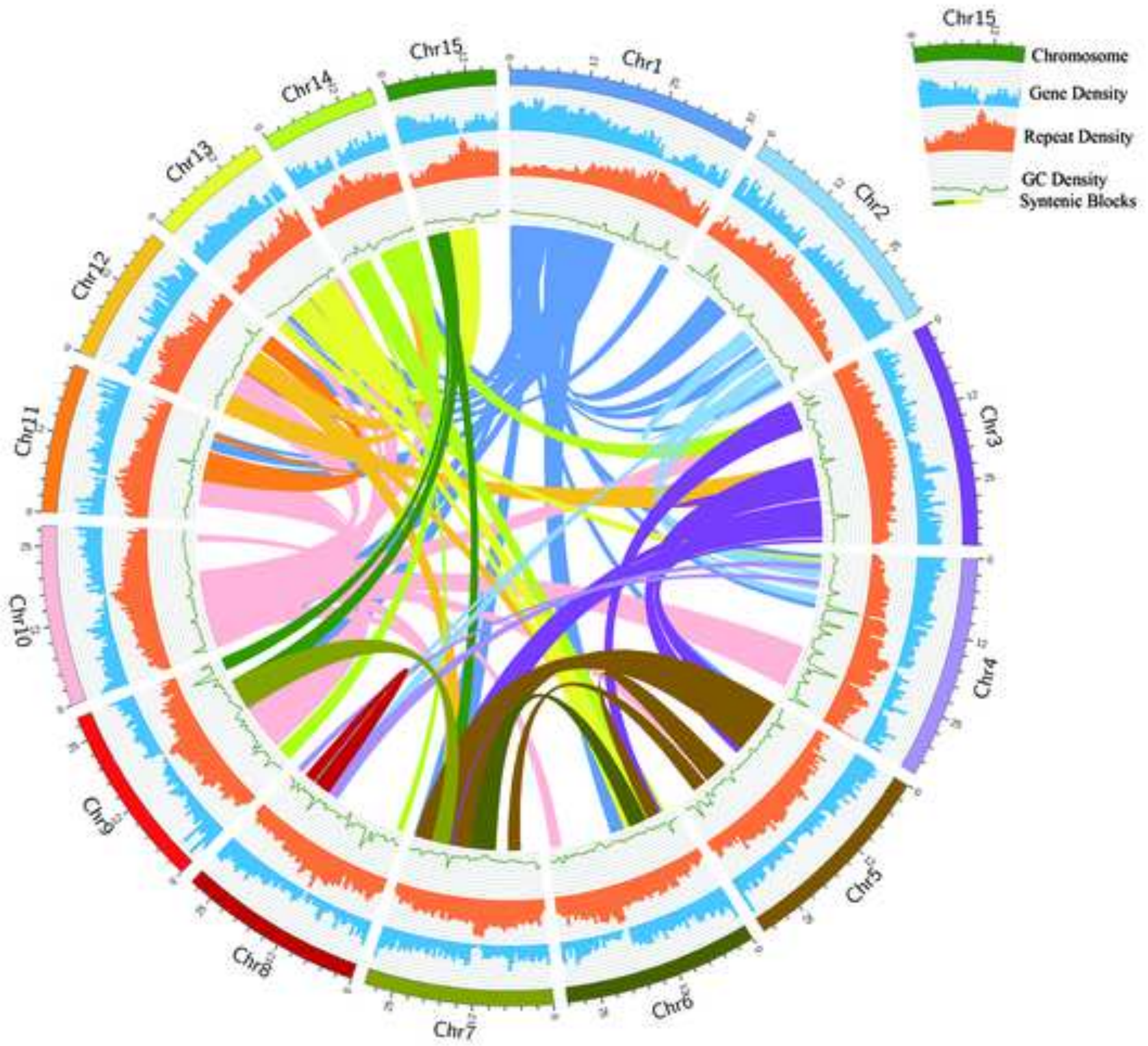

Figure 5. Phylogenomics analysis of yellowhorn genome

Click here to download Figure Figure 5.tif

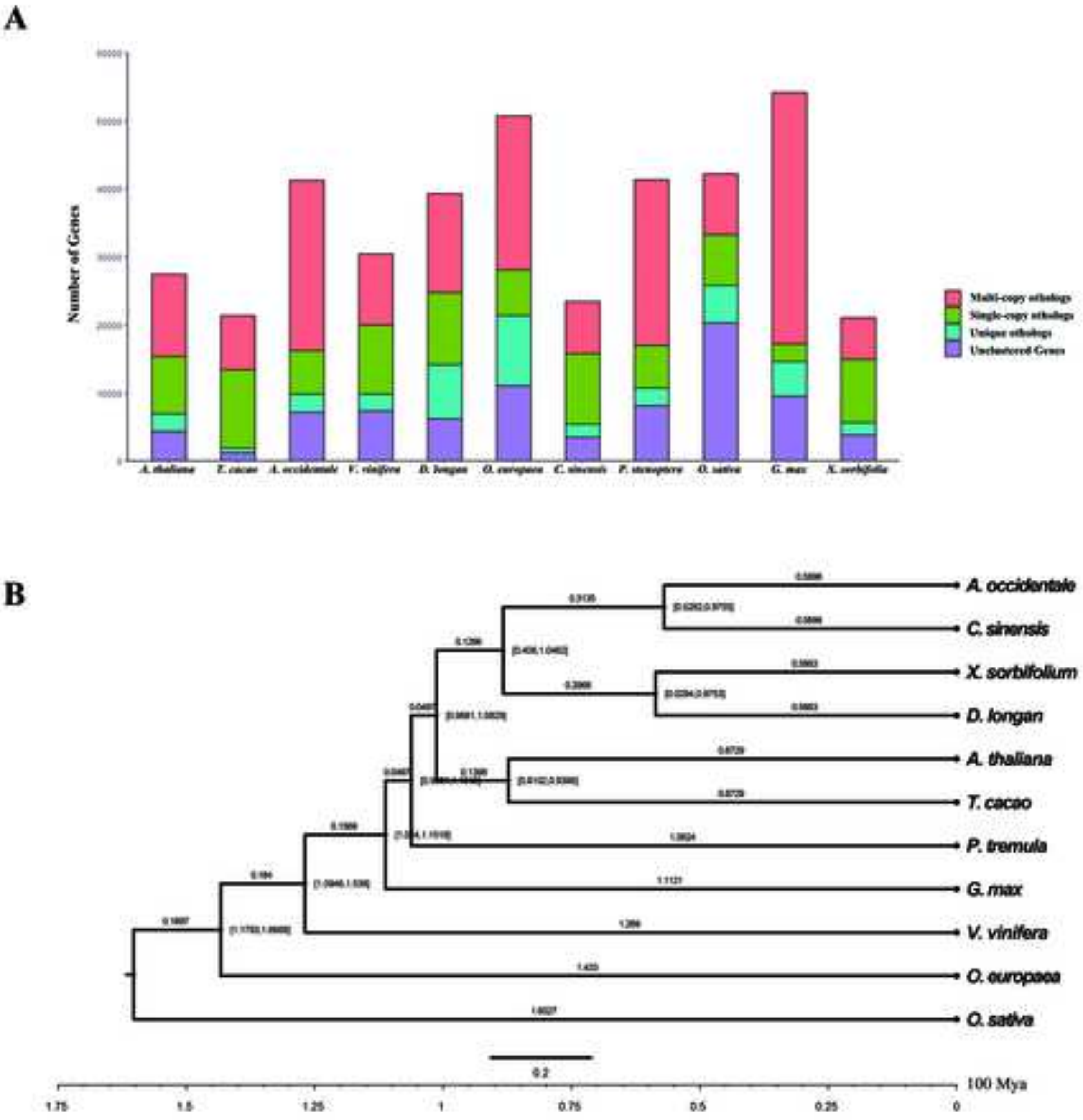

Table S1. Statistics of transcriptome sequencing data

Click here to access/download
**Supplementary Material**
Table S1.xlsx

Table S2. The features of yellowhorn genome

Click here to access/download
**Supplementary Material**
Table S2.xlsx

Table S3. Genome QC report of yellowhorn by QUAST

Click here to access/download
**Supplementary Material**
Table S3.xlsx

Table S4. Orthogroups of the protein-coding genes in yellowhorn genome and other species

Click here to access/download
**Supplementary Material**
Table S4.xlsx

Table S5. GO enrichment of yellowhorn specific genes

Click here to access/download
**Supplementary Material**
Table S5.xlsx

Table S6. Yellowhorn tissue-specific genes

Click here to access/download
**Supplementary Material**
Table S6.xlsx

Table S7. GO enrichment of yellowhorn tissue-specific genes

Click here to access/download
**Supplementary Material**
Table S7.xlsx

Figure S1. Estimation of genome size based on 111-mer distribution

Click here to access/download
**Supplementary Material**
Figure S1.tiff

Figure S2. Karyogram of yellowhorn superior ‘WF18’

Click here to access/download
**Supplementary Material**
Figure S2.tif

Personal Cover Click here to download Personal Cover Coverletter.docx

Dear Editor,

We would like to submit our manuscript entitled "**The genome assembly and annotation of yellowhorn (*Xanthoceras sorbifolium* Bunge)**" to GigaScience.

Yellowhorn (*Xanthoceras sorbifolium* Bunge), a member of the monotypic genus *Xanthocera* of family Sapindaceae, is a deciduous shrub or small tree native to north China. Seeds of yellowhorn are rich in oil containing unsaturated long chain fatty acids that have been used for producing edible oil and nervonic acid capsule. Because of the ecological, economic potential of yellowhorn, it has been received increased scientific and managerial attention with extensive studies covering its genetic improvement and intensive planting. In recent year, our research was supported by the grant from the Improved Variety Program of Shandong Province of China (2016LZGC013), the Innovative Project of Forestry Science and Technology of Shandong Province of China (LYCX05-2018-26) and the Funds of Shandong 'Double Tops' Program (SYL2017XTTD09), and focused on genomics and breeding of yellowhorn. In this paper, we first report that the whole-genome of yellowhorn was sequenced and assembled by integration of Illumina sequencing, PacBio single-molecule real-time sequencing, 10X Genomics link-reads, Bionano optical maps and Hi-C. The yellowhorn genome assembly was 439.97 Mb, which comprised of 15 pseudo-chromosomes covering 95.42% (419.84 Mb) of genome. The genome contained 21,059 protein coding genes. Of them, 18,503 (87.46%) genes were well annotated. Transcriptomic analysis showed that 341, 113, 100, 135 and 125 genes were specifically expressed in leaf, hermaphrodite flower, shoot, staminate flower and young fruit, respectively. Phylogenetic analysis suggested that yellowhorn diverged from the common ancestral of *Dimocarpus longan* approximately 58.63 million years ago. These results will facilitate to comparative genomics, gene-functional studies and molecular assisted breeding. So we wish this paper to be considered for publication in GigaScience. The authors declare that they have no competing interests. All authors read and approved the final draft of the manuscript, and agree to the submission of this paper.

We thank you for considering this work and look forward to your response. Please direct all correspondence about this manuscript to Correspondence author: **Keqiang Yang,** Email: ykq@sdau.edu.cn.

Sincerely,

**Keqiang Yang**
