# Supplementary material for: The genome assembly and annotation of yellowhorn (Xanthoceras sorbifolium Bunge)
Source: Gigascience. 2019 Jun 26;8(6):giz071. doi: 10.1093/gigascience/giz071 (PMC6593362; doi:10.1093/gigascience/giz071)
Supplement: giz071_GIGA-D-18-00410_Revision_2 [file giz071_giga-d-18-00410_revision_2.pdf]

GigaScience

The genome assembly and annotation of yellowhorn (Xanthoceras sorbifolium Bunge)

--Manuscript Draft--

**Manuscript Number:**	GIGA-D-18-00410R2
**Full Title:**	The genome assembly and annotation of yellowhorn (Xanthoceras sorbifolium Bunge)
**Article Type:**	Data Note
**Funding Information:**	the Improved Variety Program of Shandong Province of China (2016LZGC013) — Mr. Ke Qiang Yang<br>the Innovative Project of Forestry Science and Technology of Shandong Province of China (LYCX05-2018-26) — Mr. Ke Qiang Yang<br>the Funds of Shandong 'Double Tops' Program (SYL2017XTTD09) — Mr. Ke Qiang Yang
**Abstract:**	Background: Yellowhorn (Xanthoceras sorbifolium Bunge), a deciduous shrub or small tree native to north China, is of great economic value. Seeds of yellowhorn are rich in oil containing unsaturated long chain fatty acids that have been used for producing edible oil and nervonic acid capsule. However, the lack of a high-quality genome sequence hampers the understanding of its evolution and gene functions.<br>Findings: In this study, a whole-genome of yellowhorn was sequenced and assembled by integration of Illumina sequencing, PacBio single-molecule real-time sequencing, 10X Genomics link-reads, Bionano optical maps and Hi-C. The yellowhorn genome assembly was 439.97 Mb, which comprised of 15 pseudo-chromosomes covering 95.42% (419.84 Mb) of the assembled genome. The repetitive fractions accounted for 56.39% of yellowhorn genome. The genome contained 21,059 protein coding genes. Of them, 18,503 (87.46%) genes were functionally annotated at least one term by searching against the other databases. Transcriptomic analysis showed that 341, 135, 125, 113 and 100 genes were specifically expressed in hermaphrodite flower, staminate flower, young fruit, leaf and shoot, respectively. Phylogenetic analysis suggested that yellowhorn and Dimocarpus longan diverged from their most recent common ancestor approximately 46 million years ago.<br>Conclusions: The availability and subsequent annotation of yellowhorn genome, as well as the identification of tissue-specific functional genes, provides a valuable reference for plant comparative genomics, evolutionary studies and molecular design breeding.
**Corresponding Author:**	Ke Qiang Yang, Ph.D.<br>Shandong Agricultural University<br>Tai'an, Shandong Province CHINA
**Corresponding Author Secondary Information:**	
**Corresponding Author's Institution:**	Shandong Agricultural University
**Corresponding Author's Secondary Institution:**	
**First Author:**	Qiang Liang
**First Author Secondary Information:**	
**Order of Authors:**	Qiang Liang
	Huayang Li
	Shouke Li
	Fuling Yuan
	Jingfeng Sun
	Qicheng Duan
	Qingyun Li

Powered by Editorial Manager® and ProduXion Manager® from Aries Systems Corporation

	Rui Zhang
	Ya Lin Sang
	Nian Wang
	Xiangwen Hou
	Ke Qiang Yang
	Jian Ning Liu
	Long Yang
**Order of Authors Secondary Information:**	
**Response to Reviewers:**	Dear Dr. Zauner,<br>Thanks for your comments and suggestions on our revised version of the manuscript entitled “The genome assembly and annotation of yellowhorn (Xanthoceras sorbifolium Bunge)" (GIGA-D-18-00410R1). We have carefully revised the manuscript according to reviewer’s comments. Meanwhile, we have checked and confirmed our GigaDB dataset according to the suggestions of data curators. Also, we have cited the DOI link: http://dx.doi.org/10.5524/100589 in our revised manuscript.<br>We are very happy that this article can be published in GigaScience. Thank you again! If you have any questions, please do not hesitate to contact me.<br>Sincerely yours,<br>Ke Qiang Yang<br>Response to the comments<br>Reviewer #2: The Authors appear to have gone to a great deal of effort to address all of the points I raised previously and I am satisfied with the changes they have made to the manuscript.<br>Authors’ response: We would like to sincerely thank reviewer for the valuable and helpful comments and suggestions about our manuscript.<br>I only have a few very minor additional suggested changes, relating to typographical errors:<br>Page 1, line 25: Change "recently common ancestral" to "recent common ancestor".<br>Authors’ response: Revised as required.<br>Page 3, line 77: Change "suquencing" to "sequencing".<br>Authors’ response: Revised as required.<br>Page 5, line 120: Change "agilned" to "aligned".<br>Authors’ response: Revised as required.<br>Page 8, line 209: Change "origent" to "orientate".<br>Authors’ response: Revised as required.<br>Page 9, line 256: Change "contained" to "containing".<br>Authors’ response: Revised as required.<br>Page 12, line 356: Change "leat" to "least".<br>Authors’ response: Revised as required.<br>Page 14, line 412: Change "represnts" to "represents".<br>Authors’ response: Revised as required.<br>Table 4: Correct spelling of "yellowgorn" to "yellowhorn". [this was also flagged up in my initial review]<br>Authors’ response: As suggested, we rectified the spelling mistakes, and also modified the format of Table 4 in revised manuscript.
**Additional Information:**	
**Question**	**Response**
Are you submitting this manuscript to a special series or article collection?	Yes
Please select an option from the menu:<br>as follow-up to "Are you submitting this manuscript to a special series or article collection?"	Functional Metagenomics
**Experimental design and statistics**	Yes

Full details of the experimental design and statistical methods used should be given in the Methods section, as detailed in our Minimum Standards Reporting Checklist. Information essential to interpreting the data presented should be made available in the figure legends.<br><br>Have you included all the information requested in your manuscript?	
**Resources**<br><br>A description of all resources used, including antibodies, cell lines, animals and software tools, with enough information to allow them to be uniquely identified, should be included in the Methods section. Authors are strongly encouraged to cite Research Resource Identifiers (RRIDs) for antibodies, model organisms and tools, where possible.<br><br>Have you included the information requested as detailed in our Minimum Standards Reporting Checklist?	Yes
**Availability of data and materials**<br><br>All datasets and code on which the conclusions of the paper rely must be either included in your submission or deposited in publicly available repositories (where available and ethically appropriate), referencing such data using a unique identifier in the references and in the "Availability of Data and Materials" section of your manuscript.<br><br>Have you have met the above requirement as detailed in our Minimum Standards Reporting Checklist?	Yes

Manuscript
Click here to view linked References
Click here to access/download;Manuscript;YellowhornLiang2019-4-23

Title：The genome assembly and annotation of yellowhorn (*Xanthoceras sorbifolium* Bunge)

Qiang Liang[1†], Huayang Li[2†], Shouke Li[3], Fuling Yuan[1], Jingfeng Sun[1], Qicheng Duan[1], Qingyun Li[2], Rui Zhang[2], Ya Lin Sang[1], Nian Wang[1], Xiangwen Hou[4], Ke Qiang Yang[1]*, Jian Ning Liu[4]*, Long Yang[2]*

* **Correspondence**: yangwere@126.com; jnliu@kegene.com; yanglong1020@163.com

† **Equal contributors**

[1] College of Forestry, Shandong Agricultural University, Tai'an 271018, China.

[2] College of Plant Protection, Shandong Agricultural University, Tai'an 271018, China.

[3] Worth Agricultural Development Co. Ltd., Weifang 262100, China.

[4] KeGene Science & Technology Co. Ltd., Tai'an 271018, China

Abstract

Background: Yellowhorn (*Xanthoceras sorbifolium* Bunge), a deciduous shrub or small tree native to north China, is of great economic value. Seeds of yellowhorn are rich in oil containing unsaturated long chain fatty acids that have been used for producing edible oil and nervonic acid capsule. However, the lack of a high-quality genome sequence hampers the understanding of its evolution and gene functions.

Findings: In this study, a whole-genome of yellowhorn was sequenced and assembled by integration of Illumina sequencing, PacBio single-molecule real-time sequencing, 10X Genomics link-reads, Bionano optical maps and Hi-C. The yellowhorn genome assembly was 439.97 Mb, which comprised of 15 pseudo-chromosomes covering 95.42% (419.84 Mb) of the assembled genome. The repetitive fractions accounted for 56.39% of yellowhorn genome. The genome contained 21,059 protein coding genes. Of them, 18,503 (87.46%) genes were functionally annotated at least one term by searching against the other databases. Transcriptomic analysis showed that 341, 135, 125, 113 and 100 genes were specifically expressed in hermaphrodite flower, staminate flower, young fruit, leaf and shoot, respectively. Phylogenetic analysis suggested that yellowhorn and *Dimocarpus longan* diverged from their most recent common ancestor approximately 46 million years ago.

Conclusions: The availability and subsequent annotation of yellowhorn genome, as well as the identification of tissue-specific functional genes, provides a valuable reference for plant comparative genomics, evolutionary studies and molecular design breeding.

Keywords: Yellowhorn (*Xanthoceras sorbifolium* Bunge); PacBio sequencing; BioNano Genomics; 10X Genomics Chromium; High-through chromosome conformation capture; Illumina Paired End sequencing

Data Description

Introduction

Yellowhorn (*Xanthoceras sorbifolium* Bunge, NCBI: txid99658), the single species of genus *Xanthocera* (Sapindaceae), is a deciduous shrub or small tree, naturally occurring to hills and slopes in northern China [1-3]. Yellowhorn is resistant to cold, drought, and salinity [4, 5] and is of important ecological, economic and pharmacological value [6]. Yellowhorn is an andromonoecious plant which has both hermaphrodite and staminate flowers, and produces capsular fruits from hermaphrodite with seeds rich in oil (49.77 ﹣ 68.30% of kernel), which contains 85 ﹣ 93% unsaturated fatty acids, being especially remarkable the content in nervonic acid [5, 7]. Stems and fruits of yellowhorn were used in folk medicine in Inner Mongolia for the treatment of rheumatism, gout and enuresis of children [8]. Moreover, different yellowhorn tissues contain multiple bioactive compounds, including triterpenoid saponins, barringenol-like triterpenoids, which have been found to possess antitumor and anti-inflammatory activities, as well as potentiality against Alzheimer's disease [8-12].

The Sapindaceae family (also known as the Soapberry family), comprises of 142 genera and 1,900 species including important tropical fruits and woody oil-bearing plant, such as *Dimocarpus longan*, *Litchi chinensis*, *Nephelium lappaceum*, *Sapindus mukorossi* and yellowhorn [13, 14]. The genome of *D. longan* has been sequenced and assembled recently [15]. The chloroplast genome of yellowhorn has been assembled and characterized using Illumina pair-end sequencing data [16]. Genes regulating oil accumulation and fertilized ovules development have been identified in yellowhorn [17, 18]. Despite the increasing availability of genetic resources with research and economic value, fully annotated genome is currently unavailable for yellowhorn.

In this study, a high-quality draft genome of yellowhorn was sequenced and assembled by integration of Illumina sequencing, PacBio single-molecule real-time sequencing, 10X Genomics link-reads, Bionano optical maps and Hi-C. Functional annotation for protein coding genes was performed. Tissue-specific genes were identified and analyzed through transcriptomic approaches. Our study will facilitate comparative genomics, gene-functional studies and molecular assisted breeding in the near future.

Methods

Plant material

The yellowhorn superior tree (voucher No. 'WF18') with high seed yield and high oil content in kernel was conserved at the Forestry Experimental Station of Shandong Agricultural University, Tai'an, Shandong, China (36°10'16'' E, 117°08'56'' N), and was employed for genome sequencing (Figure 1). Genomic DNA was extracted from freshly flushed leaf of WF18 tree using NucleoSpin Plant II (MachereyeNagel, Düren, Germany) and Bionano Prep Plant Tissue DNA Isolation Protocol (Bionano Genomics, San Diego, CA, USA). The quality and quantity of DNA was assessed using 0.8% agarose gels and Qubit fluorimeter (Invitrogen, Carlsbad, CA, USA). Total RNA was isolated using GeneJET Plant RNA Purification Mini Kit (Thermo Fisher Scientific, Waltham, Massachusetts, USA) from five tissues of WF18 tree including hermaphrodite flower, staminate flower, young fruit, leaf and shoot, and quantified by NanoDrop ND-2000 (Thermo Fisher Scientific, Waltham, Massachusetts, USA). RNA integrity was assessed using Agilent Bioanalyzer 2100 (Agilent Technologies, Santa Clara, California, USA). The sample with integrity number greater than 8 was used for libraries construction. For Hi-C library construction, about 5 g freshly flushed leaves were crosslinked with 1% formaldehyde for 10 minutes at room temperature, which was then quenched with a final concentration of 0.125 mol/L glycine. The crosslinked leaf tissues were used for isolating intact nuclei according to the previously reported method [19].

Genomic DNA sequencing

For Illumina sequencing, two libraries with insert sizes of 280 bp and 450 bp were constructed using NEBNext Ultra II DNA Library Prep Kit (New England Biolabs, Ipswich, MA, USA). The libraries were then sequenced on an Illumina HiSeq X Ten System using a PE-150 module and 172 Gb raw data was generated. The quality of all raw reads was assessed using FASTQC v. 0.11.7 with default parameter settings. The adaptors and low-quality bases were trimmed using Trimmomatic v. 0.38 (Trimmomatic, RRID:SCR_011848) with default parameter settings [20]. Approximate 164.79 Gb (~375 × of assembled genome size 439.97 Mb) clean reads were obtained for pre-*de novo* genome assembly (Table 1).

For PacBio long reads sequencing, a 20-kb single-molecule real-time DNA sequencing library was constructed according to the manufacturer's protocol (Pacific Biosciences, Menlo Park, CA, USA).

The libraries were used for sequencing on the PacBio Sequal platform and yielded over 70.62 Gb (~ 160 × of assembled genome size) subreads.

The library of 10X Genomics was prepared using the Chromium Gel Bead and Library Kit (10X Genomics, Pleasanton, CA, USA) and the Chromium instrument (10X Genomics, Pleasanton, CA, USA) following the manufacturer's protocol. The barcoded library was sequenced on an Illumina NovaSeq 6000 system. The BCL files were demultiplexed and converted to fastq files using Supernova mkfastq (v. 2.0.0) with default parameter settings. After trimming off the first 23 bases from the beginning of read one of each pair (the 16-base 10X barcode plus 7 additional bases) by Supernova (v. 2.0.0) with default parameter settings, around 457.40 Mb reads with a mean length of 138.5 bp were generated. The fraction of Q30 in read 2 was 83.42% (Table 1).

Two Bionano optical maps were analyzed with Saphyr's streamlined workflow (BioNano Genomics, San Diego, CA, USA). High-molecular-weight DNA was treated with Nt. BspQI and Nt. BssSI nicking endonucleases (New England Biolabs, Ipswich, MA, USA) respectively. Fluorescent nucleotides were incorporated by nick translation (Bionano Prep Labeling - NLRS Protocol). After repairing the nicks, DNA sample was electrophoresed into massively parallel nanochannels imaging. More than 325 Gb (from Nt. BspQI) and 266 Gb (from Nt. BssSI) image data was collected with a minimum molecule length of 150 kb respectively (Table 1).

Hi-C library was generated using DpnII restriction enzyme following *in situ* ligation protocols [21]. The DpnII-digested chromatin was end-labeled with biotin-14-dATP (Thermo Fisher Scientific, Waltham, Massachusetts, USA) and used for *in situ* DNA ligation. The DNA sample was extracted and purified, and then sheared using Covaris S2 (Covaris, Woburn, Massachusetts, USA). After A-tailing, pull-down and adapter ligation, the DNA library was sequenced on Illumina HiSeq X Ten System using a PE-150 module. More than 133.76 Gb (~304 × of assembled genome size) clean data was generated after trimming low-quality reads and removing adaptors by Trimmomatic v. 0.38 with default parameter settings (Table 1).

Transcriptome sequencing

The RNA library was constructed using the TruSeq RNA Sample Preparation Kit v2 (Illumina, San Diego, CA, USA) and the dUTP method [22]. The five RNA libraries with insert size around 350 bp were sequenced on Illumina HiSeq 4000 System using PE-150 module. The quality of all raw reads was assessed using FASTQC v. 0.11.7 with default parameter settings. The adaptors and low-quality

bases were trimmed using Trimmomatic v. 0.38 with default parameter settings [20]. As a result, more than 44.42 Gb clean data were generated (Table S1). The quality-checked sequencing reads were aligned to assembled genome using HISAT2 v.2.1.0 in strand-specific mode [23, 24] and the result showed that the genome mapping rate was 75.68%. The quality-checked reads were also aligned to assembled genome by Tophat v. 2.1.2 in strand-specific mode with a minimum intron length of 20 bp and a maximum intron length of 20 kb [25]. The transcripts were assembled using StringTie v. 1.3.4d with default parameters [23, 26]. The abundance of gene expression was estimated using the 'scaledTPM' method in txImport v. 1.8.0 package with default parameter settings [27]. GO functional enrichment analysis was performed based on the comparison with all protein-coding genes assigned to the GO terms using Fisher's exact test implemented in topGO package v. 2.3.4 with default parameters. In addition, the quality-checked sequencing reads were also *de novo* assembled with genome-guided or *de novo* model using Trinity v. 2.5.1 (Trinity, RRID:SCR_013048) [28] in strand-specific mode with min_kmer_cov 2 and min_glue 5. All assembled transcritps were further incorporated to train *ab initio* predictors for gene prediction (see below, “Genome annotation”).

Karyotype analysis and Genome size estimation

Flower buds (2 - 2.5 mm) of WF18 tree were collected at 8: 00 to 11: 00 am of early April days in 2017, fixed directly in Carnoy’s solution (ethanol: acetic acid, 3:1) at 4℃ for 24 h. Flower buds were hydrolyzed in 1 mol/L HCl at 60℃ for 5 min, and then washed in distilled water for 3 minute. Anthers were obtained as previously described [29]. At least five dispersive meiosis metaphase plates for each karyotype were observed using an Eclipse Ni-U photomicroscope (Nikon, Tokyo, Japan) equipped a DS-Ri2 high-sensitivity camera (Nikon, Tokyo, Japan) with a Y-TV55 TV adapter (Nikon, Tokyo, Japan) on the trinocular tube. The images were captured and the chromosome length (CL), long arm length (LL) and short arm length (SL) of each chromosome were measured by imaging software NIS-Elements D v5.11.00 (Nikon, Tokyo, Japan). Then karyotypes were organized with Photoshop v CS2 (Adobe, San Jose, CA, USA) and an ideogram was generated based on the haploid set length (HSL), the relative length of the short arm ($S = SL/HSL \times 100\%$), the relative length of the long arm ($L = LL/HSL \times 100\%$) and the total chromosome ($TL = S + L$) suing Excel 2010 (Package of Microsoft Office 2010). The chromosomes were classified according to the specifications [30], based on the chromosome arm ratio (r) between the long and short arms ($r = L/S$): m = median (r = 1 - 1.7), sm = submedian (r = 1.7 - 3), st = subterminal (r = 3 - 7) and t = terminal (r > 7).

The yellowhorn genome size was estimated by flow cytometry [31]. Fresh leaves were chopped with a razor blade in a Petri dish containing 1 mL of Otto I buffer (0.1 mol/L citric acid monohydrate, 0.5% (v/v) Tween-20, pH 2-3) and then filtered through a 50 µm nylon mesh and centrifuged at 100 g for 8 min. The pellet was resuspended in 200 µL buffer of a 1:2 mixture of Otto I and Otto II (0.4 mol/L $Na_2HPO_4 \cdot 12H_2O$) and stained with 50 µg/mL propidium iodide (PI) including 50 µg/mL RNase. Four replicates were analyzed. For each replicate, over 5000 nuclei were measured using an Elite flow cytometer (Becton Dickinson, San Jose, CA, USA). The coefficient of variation of the histogram peak was below 5%. The species of *Solanum pimpinellofolium* LA1589 with draft genome size of 739 Mb was used as external reference standards [32]. The yellowhorn genome size was estimated based on k-mer frequency spectrum. The sequence reads from Illumina insert size of 280 and 450 bp libraries were prepared to construct k-mer library using KMC v. 3.1.0 [33] with k-mer length ranging from 17 to 200 and parameter settings: "-m50 -cs12000". GenomeScope v. 1.0 [34] was used to estimate genome size and evaluate genome heterozygosity based on k-mer frequency spectrum calculating from KMC.

Genome assembly by PacBio long reads

The genomic contigs were assembled based on PacBio subreads using Falcon v. 0.7.0 (Falcon, RRID:SCR_016089) [35]. Firstly, raw subreads were aligned to each other for error correction using Daligner v. 1.0 (Daligner, RRID:SCR_016066) [36] with following parameter settings: "sge_option_da = -pe smp 4 -q bigmem; sge_option_la = -pe smp 20 -q bigmem; pa_DBsplit_option = -a -x500 -s100; pa_HPCdaligner_option = -v -B128 -t16 -e0.8 -M24 -l3200 -k18 -h480 -w8 -s100; pa_concurrent_jobs = 8". Then overlapped error-corrected reads were processed to generate consensus reads by a binary executable LA4Falcon to script "fc_consensus.py" with following parameter settings: "falcon_sense_option = --output_multi --min_cov_aln 4 --min_idt 0.70 --min_cov 4 --max_n_read 200 --n_core 8; cns_concurrent_jobs = 8". Furthermore, length_cutoff 2,000, 3,000 and 5,000 were chosen respectively to filter raw reads in the first round for error correction. In the second round, length_cutoff_pr 5,000, 8,000 and 10,000 were chosen for assembling overlapping step respectively to obtain consensus overlapping reads with following parameter settings: "sge_option_pda = -pe smp 6 -q bigmem; sge_option_pla = -pe smp 16 -q bigmem; ovlp_concurrent_jobs = 8; ovlp_DBsplit_option = -s100; ovlp_HPCdaligner_option = -v -B128 -M24 -k24 -h1024 -e.9 -l2500 -s100". The consensus overlapping reads were filtered with following parameters: "overlap_filtering_setting = --max_diff 80 -

-max_cov 80 --min_cov 2 --n_core 12” and used to construct string graphs by script “fc_ovlp_to_graph.py” using the default parameters.

The draft genomic contigs were polished using PacBio long reads and Illumina paired-end reads. Firstly, the PacBio long reads were mapped to the genomic contigs using Pbalign v. 0.3.1 with default parameter settings. The self-polished consensus contigs were generated using Arrow algorithm of variantCaller tool within GenomicConsensus package v. 2.3.2 with default parameters. Secondly, the Illumina paired-end libraries of 280 and 450 bp were aligned to the self-polished consensus contigs with BWA-MEM algorithm with default parameter settings in the BWA package v. 0.7.17 (BWA, RRID:SCR_010910) [37] and final polished contigs were obtained using Pilon v. 1.22 (Pilon, RRID:SCR_014731) with default parameters [38].

Pseudo-chromosomes construction using 10X Genomics, BioNano optical maps and Hi-C

The polished contigs were first scaffolded with the 10X Genomics linked-reads by fragScaff v. 140324.1 [39]. By mapping the linked-reads to polished contigs with BWA MEM algorithm with the default parameter settings, the alignment of each library was sorted and merged into a bamParse file using samtools v.1.3.1 (SAMTOOLS, RRID:SCR_002105) with default parameters; and filtered with parameter “min N spacer size 3000, contig end node size 5000 and max contig end node size 10000”.

The 10X Genomics scaffolded was *in silico* digested with the nicking enzymes Nt.BspQI and Nt.BssSI, respectively, using perl script “fa2cmap_multi_color.pl” with default parameters in the Bionano Solve v. 3.1 (BioNano Genomics). Scaffold genome of *in silico* maps and each BioNano Genomics maps were processed using the hybrid scaffold algorithm with default parameter settings in the Bionano Solve v. 3.1 to directly generate a hybrid scaffold.

The gaps distributed in hybrid super-scaffolds were filled with PacBio consensus long reads by PBJelly v. 15.2.20 (PBJelly, RRID:SCR_012091) [40] with following parameter settings: “--minMatch 8 --minPctIdentity 70 --bestn 1 --nCandidates 20 --maxScore -500 --nproc 20 –noSplitSubreads”. Subsequently, the gaps were further filled with Illumina insert size of 280 bp and 450 bp libraries paried-end reads by GMcloser v. 1.6.2 [41] with parameter settings: “-l 150 -i 280 -c -n 20” for insert size of 280 bp library and “-l 150 -i 450 -c -n 20” for insert size of 450 bp library.

The gap-closed hybrid scaffolds were aligned to generate duplicate free Hi-C contacts based on *in situ* Hi-C data using Juicer pipeline v. 1.6.2 [42]. The gap-closed hybrid scaffolds were firstly *in silico* digested with the restriction enzyme DpnII using python script “generate_site_positions.py” with

default parameters in the Juicer pipeline. The cleaned Hi-C reads were then mapped to the hybrid scaffolds and processed to generate Hi-C contacts by Juicer pipeline with parameter settings: "-s DpnII -t 20". The duplicate free Hi-C contacts file (merged_nodups.txt) was used to *de novo* assembly by the 3D-DNA pipeline v. 180419 [43] with the default parameters. For the pre-processing stage, a range of iterative steps and algorithms were performed to eliminate misjoins in the input hybrid scaffolds. The scaffolding algorithm was firstly applied to order and orientate the scaffolds. With two iterations of the misjoin correction algorithm, the revised scaffolds were used as input for scaffolding algorithm to output "megascaffold" that concatenates all the pseudo-chromosomes. The megascaffold was imported to the Juicebox Assembly Tools (JBAT) v. 1.8.8 [44] for manual review and refinement.

Genome annotation

For repetitive elements detection, the RepBase plant repeat database (v. 23.06) and a *de novo* repeat library were used to annotate repeat sequences in yellowhorn genome assembly. *De novo* repetitive elements annotation was performed using RepeatModeler v. 1.0.11 (RepeatModeler, RRID:SCR_015027) with default parameter settings. All Modelerunknown repeat family's sequences were searched against UniProt plant protein database (accessed 31 Jan. 2018) using BLASTX with E value setting of 1 e-10 in the BLAST v. 2.7.1+. The blastx result was then used to exclude gene fragments from *de novo* predict repeats using ProtExcluder v. 1.2 with default parameters. Finally, the *de novo* reliable predict repeats in genome assembly and repetitive elements in RepBase were annotated by running RepeatMasker v. 4.07 (RepeatMasker, RRID:SCR_012954) with default parameter settings.

Gene prediction was performed by combining the evidence obtained from *ab initio* predictors based on Hidden Markov Model, spliced transcripts evidence from the transcript assembly by Trinity and protein homology evidence from the proteins of related plants aligned against yellowhorn genome assembly. For *ab initio* gene prediction, three predictors namely Augustus v. 3.2.2 (Augustus: Gene Prediction, RRID:SCR_008417) [45], SNAP (accessed 28 Jul. 2006) gene finder [46] and GeneMark-ES/ET v. 4.3.5 [47] were performed on repeat-masked yellowhorn genome. Firstly, spliced transcripts generated from Trinity following *de novo* and genome-guided model were aligned against the yellowhorn genome with PASA v. 2.3.3 [48] following default parameter settings to get reliable ORFs used for training *ab initio* predictors. Augustus *ab initio* model was generated by running Augustus program with five rounds of training and 8-fold cross validation based on the best ORFs obtained from PASA.

Final gene models were predicted using *ab initio* trained model with the intron hints from RNA sequencing junctions and Trinity assembled transcripts. SNAP *ab initio* model were obtained using the same gene sets as Augustus with one round. The gene models were final predicted with trained model following default parameters. GeneMark-ES/ET gene models were predicted with intron hints under unsupervised training following default parameter settings. To predict genes based on similarity, protein sequences of *Citrus sinensis*, *D. longan*, *Theobroma cacao*, *Olea europaea*, *Anacardium occidental*, *Vitis vinifera*, *Glycine max*, *Populus tremula*, *Oryza sativa* and *Arabidopsis thaliana* were spliced-mapped to the repeat-masked yellowhorn genome assembly using Exonerate v. 2.2.0 [49] with protein2genome model at 90% identity. Gene models from *ab initio* and homology predictions were combined to get a single high-confidence gene model by EVidenceModeler (EVM) v. 2.4.0 following developer' suggestions [50]. Weights were set according to the confidence of PASA Trinity set, weight 10; Augustus gene set, weight 6; Exonerate protein homology set, weight 2; SNAP gene model set, weight 2; and GeneMark-ES/ET gene set, weight 1.

The function of predicted protein coding genes were annotated by searching against the database of NR (accessed 31 Jan 2018), UniProt (accessed 31 Jan 2018) using BLASTX with E value setting of 1 e-5, coverage ≥ 50%, identity ≥ 30% in the BLAST v. 2.7.1+. Pfam domain annotation was performed by aligned with the Pfam database (Pfam 28) (accessed 20 May 2015) using HMMER v. 3.1b2 (Hmmer, RRID:SCR_005305) with default parameters [51]. GO terms of each predicted protein coding genes were assigned using Blast2GO v. 4.1.9 with default parameter [52]. KEGG annotation was assigned by searching against KEGG GENES database in KAAS web server with bi-directional best hit [53]. CAZy annotation was implemented by aligned with CAZy database (accessed 20 Jul 2017) using dbSCAN v. 6.0 [54] following default parameter settings.

Comparative phylogenomics

The protein sequences of yellowhorn, together with *C. sinensis*, *D. longan*, *T. cacao*, *O. europaea*, *A. occidentale*, *V. vinifera*, *G. max*, *P. tremula*, *O. sativa*, *A. thaliana* containing only one transcript per gene were retrieved and filtered by removing redundancy of alternative spliced and low-quality proteins using the program of orthomclFilterFasta in the OrthoMCL v. 2.0.9 (OrthoMCL DB: Ortholog Groups of Protein Sequences, RRID:SCR_007839) [55] with "min_length 30 and max_percent_stop 20". The produced proteins were also manually checked and filtered away the mitochondrial and plastid genes by searching against all conserved mitochondrial and plastid genes

available from GenBank (accessed 10 Jul 2018) using BLASTP in the BLAST v. 2.7.1+ with default parameters. The all-vs-all alignment based on the filtered proteins was performed using BLASTP in the BLAST v. 2.7.1+ with following parameters: "-evalue e-5 –seg yes –outfmt 6". The blast collections were used to find pairs of proteins that are potentially orthologs, in-paralogs or co-orthologs by the program of orthomclPairs in the OrthoMCL using a cutoff of 1 e-5 and 50% match. All of the pairs were further clustered into groups using the program mcl in the OrthoMCL with parameters: "--abc -I 1.5".

The protein sequences of 195 single copy orthologous genes that shared single copy genes among the plant species were performed to generate multiple sequence alignment using MAFFT v. 7.158b (MAFFT, RRID:SCR_011811) with an accurate option (L-INS-i) [56]. After each alignment merging, GBlocks v. 0.91b [57] with default parameters was used to remove poorly aligned positions, divergent regions, and selected conserved blocks. Phylogeny was constructed using RAxMLv. 8.1.24 [58] with the evolutionary model GTR+GAMMA. A total of 1,000 rapid bootstrap inferences were performed. Divergence time of species was estimated using MCMCTree in PAML 4.9h (PAML, RRID:SCR_014932) package [59] with correlated rates clock and JC69 model settings following five MCMCTree runs. The Markov Chain Monte Carlo analysis was run on 20,000 generations with a burn-in of 2000 iterations. Divergence time estimates were extrapolated using secondary calibration points from the TimeTree database [60] for *A. thaliana* - *T. cacao* split (median 85 million years ago (MYA); 95% Confidence Interval (CI): 81 - 94 MYA), *P. tremula* - *A. thaliana* split (median 108 MYA; 95% CI: 97 - 109 MYA) and *O. sativa* - *O. europaea* split (median 149 MYA; 95% CI: 148 - 173 MYA). The phylogenetic tree was visualized in FigTree v. 1.4.3 [61].

Results and discussion

Genomic karyotype analysis and size estimation

Morphometric analysis of the chromosome pairs was revealed that the chromosome length was ranged from 1.93 - 5.07 µm, with an arm ratio ranging from 1.02 - 2.26. Nine chromosome pairs (chromosomes 2, 5, 8, 9, 10, 11, 12, 13, 14) were m and six (chromosome 1, 3, 4, 6, 7, 15) were sm. An obvious satellite was found to be located at the second pair of the one chromosome pairs. Genomic karyotype analysis showed that yellowhorn 'WF18' was a diploid plant with karyotype formula 2n = 2X = 30 = 18m (2SAT) + 12 sm (Figure S1).

The genomic size was also estimated based on k-mer frequency spectrum with different k-mer length ranging from 17 to 200. A k-mer statistics algorithm of KMC was introduced to count and manipulate k-mer sizes. With k-mer length of 61, the genomic size was estimated to be 442.33 Mb with a relatively high heterozygosis rate of 0.81% (Figure S2). The haploid genome size of yellowhorn was also measured by flow cytometry and showed the 1C genomic sequence was 433.57 Mb.

Genome sequencing and assembly

The flowchart of genome assembly and annotation was shown in Figure 2, yellowhorn genome was assembled by integration of Illumina short reads, PacBio long reads, 10X Genomics link-reads, Bionano optical maps and Hi-C short reads.

Pacbio long reads assembly: Using PacBio long reads sequencing, approximate 70.62 Gb high quality subreads was generated from a 20-kb DNA sequencing library with mean length > 8 kb and N50 length > 15 Kb (Table 2). By finding single path of each contig graphs with optimal parameter "length_cutoff 2000 and length_cutoff_pr 8000" using the graph to contig script "fc_graph_to_contig.py", the draft genomic contigs were created to be 505.79 Mb in length with N50 value of 642,338 bp for 2,002 contigs (Table 3). Assembled contigs were polished with PacBio long reads and high quality Illumina paired-end reads, resulting in 2,002 assembled contigs with 508.45 Mb in length and N50 value of 645,453 bp (Table 3).

Pseudo-chromosomes construction: The polished contigs were scaffolded with the 10X Genomics linked-reads and assembled to be 513.92 Mb in length with N50 value of 2,334,658 bp for 707 scaffolds (Table 3). By hybriding the two BioNano Genomic maps with the *in silico* maps of genome assembly, 29 super-scaffolds were generated in length of 461.66 Mb with N50 value of 29.98 Mb (Table 3). The number of 7,192 (34.73 Mb) gaps distributed in hybrid super-scaffolds were firstly filled with PacBio consensus long reads, leading to 6,015 gaps being resolved. Subsequently, the gaps were further filled with Illumina insert size of 280 bp and 450 bp libraries paired-end reads, giving rise to 77 gaps were closed. Over, 6,092 gaps were filled which reduced the N bases to 29.06 Mb representing 6.29% of hybrid super-scaffolds. To get the chromosome length of scaffolds, the *in situ* Hi-C data was used to generate yellowhorn pseudo-chromosomes with 439.97 Mb in final genome assembly size. Fifteen pseudo-chromosomes were assembled which covered 95.42% (419.84 Mb) of genome assembly (Figure 3). The maximal length of the pseudo-chromosomes was 39.12 Mb and minimum one was 17.23 Mb (Figure 4, Table 3, and Table S2).

Genome assembly assessment

The completeness of genome assembly was assessed by searching against 1,440 embryophyta specific single copy orthologs in genome assembly assessment mode using BUSCO v. 3.0.2 (BUSCO, RRID:SCR_015008) [62] with default parameters. In total, 1,218 (84.58%) complete BUSCOs and 23 (1.60%) fragmented BUSCOs were identified in the yellowhorn genome (Table 4). A total of 85.10% *de novo* assembled RNA-sequencing transcripts of five tissue types were mapped to yellowhorn genome using BLAT v. 3.2.19 (BLAT, RRID:SCR_011919) [63] with identity ≥ 98% and coverage ≥ 50% of each transcript. The genome assembly was also evaluated by QUAST v. 5.0.0 [64] with default parameters. The result showed that NG50 value of 28.89 Mb which represents the length of contigs covering at least half of genome assembly was close to N50 value of 29.43 Mb. It was indicated that the genome assembly was in high quality (Table S3).

Genome characterization

The repetitive fractions represented 56.39% of the yellowhorn genome assembly with repetitive elements and SSRs accounted for 54.81% and 1.58%, respectively. Therefore, comparing the content of repetitive elements with other reported closely related species, the content of repeat fractions in current yellowhorn genome assembly was relatively higher than that of *A. thaliana* (13.2%) [65], *Thellungiella salsuginea* (52%) [66], *Brassica oleracea* (48.8%) [67], *Arabidopsis lyrata* (35%) [68], *Brassica napus* (55.59%) [69], *Citrus sinensis* (20.5%) [70], *Theobroma cacao* (25.7%) [71], *D. longan* (52.87%) [15], and *Durio zibethinus* (54.8%) [72], but lower than that of *Gossypium raimondii* (57%) [73]. Moreover, LTR/Copia and LTR/Gypsy repeats were the most abundant repetitive elements, accounting for 11.91% and 11.68% of the assembled genome, respectively (Table 5).

To annotate yellowhorn genome for protein-coding genes, a comprehensive strategy that integrated *ab initio* predictors, protein homology searches and *de novo* assembled transcripts. After *ab initio* gene prediction with the trained optimal parameters, 20,980 genes from Augustus prediction, 28,134 genes from SNAP and 32,205 genes from GeneMark-ES/ET were predicted. For protein-based homology searches, 61,138 protein sequences were collected and spliced aligned to yellowhorn genome assembly to get homology gene sets. A total of 21,157 predicted genes were obtained by integration of all gene sets using EVM. After UTRs updating by running PASA on three rounds, 21,059 predicted protein coding genes with 44,283 transcripts were obtained in the final gene models. Among these gene sets, 20,952 gene models with 44,078 transcripts were allocated in the fifteen pseudo-chromosomes. All

transcripts have an average length of about 7,040 bp, a mean coding sequence length of 201.62 bp and an average of 15.61 exons per gene models. To explore the function of predicted gene models, all predicted genes were annotated by searching against the database of NR, UniProt, Pfam, GO, KEGG and CAZy. Finally, 18,503 gene models accounted for 87.46% of all gene sets were functionally annotated with at least one term.

Comparative phylogenomics

Gene families were clustered based on yellowhorn and other plant species using OrthoMCL. In total, 27,347 groups were constructed, of which 5,484 groups contained sequences from all species, 1,496 groups from at least two species and 10,367 groups from only one species (Figure 5a). Meanwhile, 462 groups containing 1,789 genes were further identified as yellowhorn specific. GO enrichment by topGO showed that “oxidation-reduction process” ($P = 1.7 \times 10^{-10}$), “defense response” ($P = 1.8 \times 10^{-6}$), “oxidoreductase activity” ($P = 5.8 \times 10^{-12}$) and “membrane” ($P = 6.5 \times 10^{-6}$) were the extremely significantly enriched function categories (Table S4).

The 195 single copy orthologous genes in yellowhorn genome assembly and other ten plant species were used to investigate the evolution of yellowhorn (Table S5). RaxML was used to construct phylogenetic trees with the evolutionary model GTR+GAMMA. The divergence time was estimated using MCMCTree in five independent MCMCTree runs and extrapolated using secondary calibration points from the TimeTree database. The phylogenetic tree was visualized in FigTree and suggested that yellowhorn and *D. longan* diverged from their most recent common ancestor approximately median 46 MYA with 95% CI: 36.64 - 54.58 MYA (Figure 5b).

Transcriptome analysis of tissue-specific expression

To explore the tissue-specific genes, we performed transcriptomic analysis of five yellowhorn tissues including hermaphrodite flower, staminate flower, young fruit, leaf, and shoot. A total of 814 tissue-specific genes including 45 transcription factors were obtained. Of which 341, 135, 113, 125 and 100 genes were specifically expressed in hermaphrodite flower, staminate flower, young fruit, leaf and shoot, respectively (Figure 6a, Table S6). GO enrichment of hermaphrodite flower-specific genes showed that the function of “oxidation-reduction process” ($P = 8.83 \times 10^{-3}$), “defense response” ($P = 3.87 \times 10^{-2}$), “monooxygenase activity” ($P = 1.6 \times 10^{-4}$), “oxidoreductase activity” ($P = 6.78 \times 10^{-3}$) and “membrane part” ($P = 3.47 \times 10^{-2}$) were significantly enriched. “Growth related” ($P = 3.6 \times 10^{-3}$) and “membrane part” ($P = 4.4 \times 10^{-2}$) were significantly enriched functions in leaf. For shoot-specific

genes, “response to stress” (P = 1.31 × 10^{-2}), “regulation of developmental process” (P = 1.16 × 10^{-2}) and “extracellular region” (P = 1.9 × 10^{-2}) were significantly enriched. The GO terms of “negative regulation of flower development and reproductive process” (P = 4.9 × 10^{-4}), “oxidoreductase activity” (P = 4.99 × 10^{-2}) and “membrane” (P = 3.0 × 10^{-3}) were mostly enriched in staminate flower. Additionally, GO enrichment of young fruit-specific genes showed that “metabolic process” (P = 4.3 × 10^{-2}), “binding” (P = 2.7 × 10^{-3}) and “lyase activity” (P = 1.29 × 10^{-2}) were the most enriched functions (Figure 6b, Table S7).

Functions of the specific genes revealed correlate well with the biological roles of the tissues by previous studies. For instance, hermaphrodite flower contains both stamens and pistils, and gives rise to fruits after fertilization [74]. Consistently, a number of hermaphrodite flower-specific genes have been shown to be involved in gametophytic development, fertilization and seed development. Of them, *AGL66* (XS01G01870) is expressed preferentially in pollen and participate in the regulation of male gametophytes in the model plant Arabidopsis. Double mutations of *AGL66* and *AGL104* leads to decrease of pollen viability [75]. *MYB39* (XS01G02268) is involved in microsporogenesis in apple (*Malus domestica*), such as suppressing *MYB39* expression in pollen reduced pollen tube growth [76]. *MYB64* (XS02G10689), together with *MYB119* regulate cellularization and differentiation during female gametogenesis, becuase ametophytes of *myb64 myb119* double mutant fail to initiate the FG5 transition, giving rise to uncellularized gametophytes with supernumerary nuclei [77]. Moreover, egg cell-secreted protein 1, which known as EC1 (XS05G15286), is responsible for sperm activation during fertilization [78]. *Exo70A1* (XS05G14582), which encodes a putative exocyst subunit, regulates both pollen-pistil interaction and localized deposition of seed coat pectin [79, 80]. *AGL62* (XS03G11771) encode a MADS domain transcription factor, controls cellularization during endosperm development [81]. Another MADS gene *PHERES1* (XS14G07914) has also been proven to be involved in seed development [82]. In addition, *DIVARICATA* (XS07G17645) a MYB family transcription facto controlling the dorsoventral asymmetry of flowers in *Antirrhinum*, was specifically expressed in hermaphrodite flower, implying that the regulatory mechanisms underlying corolla formation in the two flower types of yellowhorn might be different [83]. These results indicated that identification and analyses of tissue-specific genes provided clues for understanding the molecular functions of separate tissues of yellowhorn.

Figure and Table Legends

Figure 1. Morphological characteristic of yellowhorn superior 'WF18'. (A) Raceme and shoot. (B) Hermaphrodite flower at 1 DPA (days post flower), 3DPA, 5DPA. (C) Capsular fruits. (D) Seeds and kernel.

Figure 2. Flowchart of genome assembly and annotation.

Figure 3. Contact maps of Hi-C links among chromosomes. Blue square represents draft scaffold. Green square represents chromosome-length superscaffold. The color bar illuminated the Hi-C contact density in the plot.

Figure 4. Yellowhorn genome features. The chromosomes size in Mb scale. The denotation of the distribution of gene density, repeat density and GC density are listed on the top right corner. The syntenic blocks were represented by curves in the center of the graph. Figure was created by circos software package v. 0.69.

Figure 5. Phylogenomics analysis of yellowhorn genome. (A) OrthoMCL clusters of yellowhorn and ten other species. (B) Phylogenetic tree and estimated divergence time of yellowhorn and ten other species. The numbers above the branches are the predicted divergence time. The numbers below the branches are bootstrap support value. The light blue bars at the internodes represent 95% confidence interval. The bottom scale-bar shows divergence time with 1 time unit representing of 100 MYA.

Figure 6. Tissue-specific gene analysis. (A) Venn diagram showing shared and unique genes among five tissues. Numbers represent the number of genes in unique or shared. (B-D) GO enrichment of tissue-specific genes. The node size represents the gene numbers enriched in each GO category. The color bar illuminates p-value from red (low) to blue (high) in the plot.

Table 1. Statistics of Illumina, 10X Genomics, and Hi-C sequencing data

Table 2. Statistics of PacBio Sequal sequencing data. *Coverage (X) = (read count * read length) / estimated genome size.

Table 3. Summary of yellowhorn genome assembly.

Table 4. BUSCO assessment of yellowhorn genome assembly.

Table 5. Repeat content of yellowhorn genome assembly. *DNA: DNA transposons; LINE: long interspersed nuclear elements; SINE: short interspersed nuclear elements; LTR: long terminal repeat; RC: rolling circle replication; SSRs: simple sequence repeats.

Additional files

Figure S1. Karyogram of yellowhorn superior ‘WF18’. (A) Chromosome at diakinesis of pollen mother cell meiophase. Bar = 5μm. (B) Yellowhorn superior ‘WF18’ was a diploid plant, 2n = 2X = 30. (C) Ideogram (Karyotype formula of yellowhorn superior ‘WF18’ was 2n = 2X = 30 = 18m (2SAT) +12 sm).

Figure S2. Yellowhorn genome evaluation and estimation by GenomeScope. The X-axis represents k-mer coverage. The Y-axis represents k-mer frequency spectrum numbers. With k-mer length of 61, the genomic size was estimated to be 442.33 Mb with the heterozygosis rate of 0.81%.

Table S1. Statistics of transcriptome sequencing data.

Table S2. The features of yellowhorn genome assembly.

Table S3. Genome QC report of yellowhorn genome assembly by QUAST.

Table S4. GO enrichment of yellowhorn specific genes.

Table S5. The 195 single copy orthologous genes in yellowhorn genome assembly and other ten species.

Table S6 Yellowhorn tissues-specific genes.

Table S7. GO enrichment of yellowhorn tissues-specific genes.

Funding

This work was financially supported by the Improved Variety Program of Shandong Province of China (2016LZGC013), the Innovative Project of Forestry Science and Technology of Shandong Province of China (LYCX05-2018-26) and the Funds of Shandong ‘Double Tops’ Program (SYL2017XTTD09).

Abbreviations

BUSCO: Benchmarking Universal Single-Copy Orthologs;

Hi-C: High-through Chromosome conformation capture

QUAST: Quality Assessment Tool for Genome Assemblies.

SSRs: simple sequence repeats

LINEs: long interspersed nuclear elements

PASA: Program to Assemble Spliced Alignments

ORFs: open reading frames.

UTR: Untranslated Region

GO: GeneOntology

KEGG: Kyoto Encyclopedia of Genes and Genomes

CAZy: Carbohydrate-Active enZYmes.

LTR: long terminal repeat

Availability of supporting data

Raw data are available via NCBI (Bioproject accession PRJNA496350, Biosample: SAMN10239523)

Other supporting data, including the genome assembly, annotations, VCF files and alignments, are available via the GigaScience database, GigaDB [84].

Software and Reference data

Software	URLs
FASTQC	http://www.bioinformatics.babraham.ac.uk/projects/fastqc/
Trimmomatic	http://www.usadellab.org/cms/index.php?page=trimmomatic/
FALCON	https://github.com/PacificBiosciences/FALCON/
pbalign	https://github.com/PacificBiosciences/pbalign/
arrow	https://github.com/PacificBiosciences/GenomicConsensus/
BWA	http://bio-bwa.sourceforge.net/
fragScaff	https://sourceforge.net/projects/fragscaff/
Solve	https://bionanogenomics.com/support-page/bionano-solve/
PBJelly	https://sourceforge.net/projects/pb-jelly/files/latest/download
GMcloser	https://sourceforge.net/projects/gmcloser/
Juicer	https://github.com/aidenlab/juicer/
BUSCO	https://busco.ezlab.org/
QUAST	http://quast.bioinf.spbau.ru/
RepeatMasker	http://repeatmasker.org/
RepeatModeler	http://www.repeatmasker.org/RepeatModeler/
Trinity	https://github.com/trinityrnaseq/trinityrnaseq/
PASA	https://github.com/PASApipeline/PASApipeline/
Augustus	http://bioinf.uni-greifswald.de/augustus/
SNAP	https://github.com/KorfLab/SNAP/
GeneMark-ES/ET	http://exon.gatech.edu/GeneMark/
Exonerate	https://www.ebi.ac.uk/about/vertebrate-genomics/software/exonerate
EVidenceModeler	http://evidencemodeler.github.io/
OrthoMCL	http://orthomcl.org/orthomcl/
topGO	http://bioconductor.org/packages/topGO/
MAFFT	https://mafft.cbrc.jp/alignment/software/
RaxML	http://evomics.org/learning/phylogenetics/raxml/
PAML	http://abacus.gene.ucl.ac.uk/software/paml.html/
Tophat	http://ccb.jhu.edu/software/tophat/index.shtml/
GenomeScope	http://qb.cshl.edu/genomescope/

KMC	http://sun.aei.polsl.pl/kmc/
**Reference data**	**URLs**
RepBase plant repeat database	https://www.girinst.org/server/RepBase/
TimeTree database	http://timetree.org/
UniProt plant protein database	ftp://ftp.uniprot.org/pub/databases/uniprot/current_release/knowledgebase/taxonomic_divisions/uniprot_sprot_plants.dat.gz
NR	ftp://ftp.ncbi.nlm.nih.gov/blast/db/FASTA/nr.gz
UniProt	ftp://ftp.uniprot.org/pub/databases/uniprot/current_release/knowledgebase/taxonomic_divisions/uniprot_sprot_plants.dat.gz
Pfam database	ftp://ftp.ebi.ac.uk/pub/databases/Pfam/releases/Pfam28.0/Pfam-A.hmm.gz
CAZy database	http://csbl.bmb.uga.edu/dbCAN/download.php
Olea europaea v1	http://olivegenome.org/downloads/
Citrus sinensis v2	http://citrus.hzau.edu.cn/orange/download/index.php
Glycine max v9.0	ftp://ftp.jgi-psf.org/pub/compgen/phytozome/v9.0/Gmax/
Arabidopsis thaliana TAIR10	https://www.arabidopsis.org/download/index-auto.jsp?dir=%2Fdownload_files%2FGenes%2FTAIR10_genome_release
Oryza sativa IRGSP-1.0	http://rapdb.dna.affrc.go.jp/download/irgsp1.html
Populus trichocarpa v3.0	https://genome.jgi.doe.gov/pages/dynamicOrganismDownload.jsf?organism=Ptrichocarpa
Vitis vinifera v2	http://genomes.cribi.unipd.it/grape/
Dimocarpus longan	ftp://penguin.genomics.cn/pub/10.5524/100001_101000/100276/
Anacardium occidentale v0.9	https://genome.jgi.doe.gov/portal/pages/dynamicOrganismDownload.jsf?organism=Aoccidentale
Theobroma cacao v2	http://cocoa-genome-hub.southgreen.fr/download

Authors' contributions

KQY conceived this genome project and coordinated research activities; LY, JNL, KQY, YLS and NW designed the experiments; LY, JNL, HL, Qiang L and XH assembled and annotated the genome; HL, Qingyun L, RZ and XH analyzed transcriptome and phylogenies; Qiang L, SL, FY, and QD collected and maintained plant materials; JS estimated genome size and analyzed karyotype. JNL, LY, KQY, Qiang L, HL, YLS and NW wrote the manuscript. All authors have read and approved the final manuscript.

Competing interests

The authors declare that they have no competing interests.

Editor's Note

Please also note another, independent Data Note published in *GigaScience*, also presenting a genome assembly of *Xanthoceras sorbifolium* [85]. We independently received two submissions on the Yellowhorn genome, from two different teams, within a short period of time. We reviewed both submissions in parallel and decided to publish them "back-to-back" in the journal, on the same day.
"

Reference

1. Nianhe X and Gadek PA. Sapindaceae. In: Wu Z, Raven PH and Hong D, editors. Flora of China: Hippocastanaceae through Theaceae. Beijing, China: Science Press; 2007. p. 5-24.

2. Chase MW, Christenhusz M, Fay M, Byng J, Judd W, Soltis D, et al. An update of the Angiosperm Phylogeny Group classification for the orders and families of flowering plants: APG IV. Botanical Journal of the Linnean Society. 2016;181 1:1-20.

3. Wang Q, Yang L, Ranjitkar S, Wang J, Wang X, Zhang D, et al. Distribution and in situ conservation of a relic Chinese oil woody species *Xanthoceras sorbifolium* (yellowhorn). Canadian Journal of Forest Research. 2017;47 11:1450-6.

4. Wang Q, Zhu R, Cheng J, Deng Z, Guan W and Elkassaby YA. Species association in *Xanthoceras sorbifolium* Bunge communities and selection for agroforestry establishment. Agroforestry Systems. 2018:1-13.

5. Venegascaleron M, Ruizmendez MV, Martinezforce E, Garces R and Salas JJ. Characterization of *Xanthoceras sorbifolium* Bunge seeds: Lipids, proteins and saponins content. Industrial Crops and Products. 2017;109:192-8.

6. Yao Z-Y, Qi J-H and Yin L-M. Biodiesel production from *Xanthoceras sorbifolia* in China: Opportunities and challenges. Renewable and Sustainable Energy Reviews. 2013;24:57-65. doi:10.1016/j.rser.2013.03.047.

7. Yu H, Fan S, Bi Q, Wang S, Hu X, Chen M, et al. Seed morphology, oil content and fatty acid composition variability assessment in yellow horn (*Xanthoceras sorbifolium* Bunge) germplasm for optimum biodiesel production. Industrial Crops and Products. 2017;97:425-30. doi:10.1016/j.indcrop.2016.12.054.

8. Xiao W, Wang Y, Zhang P, Li N, Jiang S, Wang JH, et al. Bioactive barrigenol type triterpenoids from the leaves of *Xanthoceras sorbifolia* Bunge. European Journal of Medicinal Chemistry. 2013;60:263-70. doi:10.1016/j.ejmech.2012.12.022.

9. Yu L, Wang X, Wei X, Wang M, Chen L, Cao S, et al. Triterpenoid saponins from *Xanthoceras sorbifolia* Bunge and their inhibitory activity on human cancer cell lines. Bioorganic & Medicinal Chemistry Letters. 2012;22 16:5232-8. doi:10.1016/j.bmcl.2012.06.061.

10. Wang D, Su D, Yu B, Chen C, Cheng L, Li X, et al. Novel anti-tumour barringenol-like triterpenoids from the husks of *Xanthoceras sorbifolia* Bunge and their three dimensional quantitative structure activity relationships analysis. Fitoterapia. 2017;116:51-60. doi:10.1016/j.fitote.2016.11.002.

11. Wang D, Su D, Li X-Z, Liu D, Xi R-G, Gao H-Y, et al. Barrigenol triterpenes from the husks of *Xanthoceras sorbifolia* Bunge and their antitumor activities. RSC Advances. 2016;6 33:27434-46. doi:10.1039/c6ra02706g.

12. Li Y, Xu J, Xu P, Song S, Liu P, Chi T, et al. *Xanthoceras sorbifolia* extracts ameliorate dendritic spine deficiency and cognitive decline via upregulation of BDNF expression in a rat model of Alzheimer's disease. Neuroscience Letters. 2016;629:208-14. doi:10.1016/j.neulet.2016.07.011.

13. Buerki S. Phylogeny and circumscription of Sapindaceae revisited: molecular sequence data, morphology and biogeography support recognition of a new family, Xanthoceraceae. Plant Ecology and Evolution. 2010;143 2:148-59. doi:10.5091/plecevo.2010.437.

14. Buerki S, Lowry PP, Phillipson PB and Callmander MW. Molecular Phylogenetic and Morphological Evidence Supports Recognition of Gereaua, a New Endemic Genus of Sapindaceae from Madagascar. Systematic Botany. 2010;35 1:172-80.

15. Lin Y, Min J, Lai R, Wu Z, Chen Y, Yu L, et al. Genome-wide sequencing of longan (*Dimocarpus longan* Lour.) provides insights into molecular basis of its polyphenol-rich characteristics. Gigascience. 2017;6 5:1-14. doi:10.1093/gigascience/gix023.

16. Chen S and Zhang X. Characterization of the complete chloroplast genome of *Xanthoceras sorbifolium*, an endangered oil tree. Conservation Genetics Resources. 2017;9 4:1-4.

17. Liu Y, Huang Z, Ao Y, Li W and Zhang Z. Transcriptome analysis of yellow horn (*Xanthoceras sorbifolia* Bunge): a potential oil-rich seed tree for biodiesel in China. PLoS One. 2013;8 9:e74441. doi:10.1371/journal.pone.0074441.

18. Zhou Q and Zheng Y. Comparative De Novo Transcriptome Analysis of Fertilized Ovules in *Xanthoceras sorbifolium* Uncovered a Pool of Genes Expressed Specifically or Preferentially in the Selfed Ovule That Are Potentially Involved in Late-Acting Self-Incompatibility. PLoS One. 2015;10 10:e0140507. doi:10.1371/journal.pone.0140507.

19. Sikorskaite S, Rajamaki ML, Baniulis D, Stanys V and Valkonen JP. Protocol: Optimised methodology for isolation of nuclei from leaves of species in the Solanaceae and Rosaceae families. Plant Methods. 2013;9:31. doi:10.1186/1746-4811-9-31.

20. Bolger AM, Lohse M and Usadel B. Trimmomatic: a flexible trimmer for Illumina sequence data. Bioinformatics. 2014;30 15:2114-20. doi:10.1093/bioinformatics/btu170.

21. Belaghzal H, Dekker J and Gibcus JH. Hi-C 2.0: An optimized Hi-C procedure for high-resolution genome-wide mapping of chromosome conformation. Methods. 2017;123:56-65. doi:10.1016/j.ymeth.2017.04.004.

22. Parkhomchuk D, Borodina T, Amstislavskiy V, Banaru M, Hallen L, Krobitsch S, et al. Transcriptome analysis by strand-specific sequencing of complementary DNA. Nucleic Acids Research. 2009;37 18:e123. doi:10.1093/nar/gkp596.

23. Pertea M, Kim D, Pertea GM, Leek JT and Salzberg SL. Transcript-level expression analysis of RNA-seq experiments with HISAT, StringTie and Ballgown. Nature Protocols. 2016;11 9:1650-67. doi:10.1038/nprot.2016.095.

24. Kim D, Langmead B and Salzberg SL. HISAT: a fast spliced aligner with low memory requirements. Nature Methods. 2015;12 4:357-60.

25. Kim D, Pertea G, Trapnell C, Pimentel H, Kelley R and Salzberg SL. TopHat2: accurate alignment of transcriptomes in the presence of insertions, deletions and gene fusions. Genome Biology. 2013;14 4:R36. doi:10.1186/gb-2013-14-4-r36.

26. Pertea M, Pertea GM, Antonescu CM, Chang TC, Mendell JT and Salzberg SL. StringTie enables improved reconstruction of a transcriptome from RNA-seq reads. Nature Biotechnology. 2015;33 3:290-5. doi:10.1038/nbt.3122.

27. Soneson C, Love MI and Robinson MD. Differential analyses for RNA-seq: transcript-level estimates improve gene-level inferences. F1000Research. 2015;4:1521. doi:10.12688/f1000research.7563.2.

28. Grabherr MG, Haas BJ, Yassour M, Levin JZ, Thompson DA, Amit I, et al. Full-length transcriptome assembly from RNA-Seq data without a reference genome. Nature Biotechnology. 2011;29 7:644-52. doi:10.1038/nbt.1883.

29. Baptistagiacomelli FR, Pagliarini MS and De Almeida JL. Meiotic Behavior in Several Brazilian Oat Cultivars (*Avena Sativa* L.). Cytologia. 2000;65 4:371-8.

30. Levan A, Fredga K and A. Sandberg A. Nomenclature for Centromeric Position on Chromosomes. 2009.

31. Zcaron JD, Greilhuber J and Suda J. Estimation of nuclear DNA content in plants using flow cytometry. Nature Protocols. 2007;2 9:2233-44.

32. Sato S, Tabata S, Hirakawa H, Asamizu E, Shirasawa K, Isobe S, et al. The tomato genome sequence provides insights into fleshy fruit evolution. Nature. 2012;485 7400:635-41.

33. Kokot M, Dlugosz M and Deorowicz S. KMC 3: counting and manipulating k-mer statistics. Bioinformatics. 2017;33 17:2759-61.

34. Vurture GW, Sedlazeck FJ, Nattestad M, Underwood CJ, Fang H, Gurtowski J, et al. GenomeScope: fast reference-free genome profiling from short reads. Bioinformatics. 2017;33 14:2202-4.

35. Pendleton M, Sebra R, Pang AW, Ummat A, Franzen O, Rausch T, et al. Assembly and diploid architecture of an individual human genome via single-molecule technologies. Nature Methods. 2015;12 8:780-6. doi:10.1038/nmeth.3454.

36. Myers G. Efficient Local Alignment Discovery amongst Noisy Long Reads. workshop on algorithms in bioinformatics. 2014:52-67.

37. Li H and Durbin R. Fast and accurate short read alignment with Burrows-Wheeler transform. Bioinformatics. 2009;25 14:1754-60. doi:10.1093/bioinformatics/btp324.

38. Walker BJ, Abeel T, Shea T, Priest M, Abouelliel A, Sakthikumar S, et al. Pilon: an integrated tool for comprehensive microbial variant detection and genome assembly improvement. PLoS One. 2014;9 11:e112963. doi:10.1371/journal.pone.0112963.

39. Adey A, Kitzman JO, Burton JN, Daza R, Kumar A, Christiansen L, et al. In vitro, long-range sequence information for de novo genome assembly via transposase contiguity. Genome Research. 2014;24 12:2041-9. doi:10.1101/gr.178319.114.

40. English AC, Richards S, Han Y, Wang M, Vee V, Qu J, et al. Mind the gap: upgrading genomes with Pacific Biosciences RS long-read sequencing technology. PLoS One. 2012;7 11:e47768. doi:10.1371/journal.pone.0047768.

41. Kosugi S, Hirakawa H and Tabata S. GMcloser: closing gaps in assemblies accurately with a likelihood-based selection of contig or long-read alignments. Bioinformatics. 2015;31 23:3733-41. doi:10.1093/bioinformatics/btv465.

42. Durand NC, Shamim MS, Machol I, Rao SS, Huntley MH, Lander ES, et al. Juicer Provides a One-Click System for Analyzing Loop-Resolution Hi-C Experiments. Cell Systems. 2016;3 1:95-8. doi:10.1016/j.cels.2016.07.002.

43. Dudchenko O, Batra SS, Omer AD, Nyquist SK, Hoeger M, Durand NC, et al. De novo assembly of the Aedes aegypti genome using Hi-C yields chromosome-length scaffolds. Science. 2017;356 6333:92-5. doi:10.1126/science.aal3327.

44. Durand NC, Robinson JT, Shamim MS, Machol I, Mesirov JP, Lander ES, et al. Juicebox Provides a Visualization System for Hi-C Contact Maps with Unlimited Zoom. Cell Systems. 2016;3 1:99-101. doi:10.1016/j.cels.2015.07.012.

45. Stanke M and Waack S. Gene prediction with a hidden Markov model and a new intron submodel. Bioinformatics. 2003;19 Suppl 2:ii215-25.

46. Korf I. Gene finding in novel genomes. BMC Bioinformatics. 2004;5:59. doi:10.1186/1471-2105-5-59.

47. Lomsadze A, Ter-Hovhannisyan V, Chernoff YO and Borodovsky M. Gene identification in novel eukaryotic genomes by self-training algorithm. Nucleic Acids Research. 2005;33 20:6494-506. doi:10.1093/nar/gki937.

48. Haas BJ, Delcher AL, Mount SM, Wortman JR, Smith RK, Jr., Hannick LI, et al. Improving the Arabidopsis genome annotation using maximal transcript alignment assemblies. Nucleic Acids Research. 2003;31 19:5654-66.

49. Slater GS and Birney E. Automated generation of heuristics for biological sequence comparison. BMC Bioinformatics. 2005;6:31. doi:10.1186/1471-2105-6-31.

50. Haas BJ, Salzberg SL, Zhu W, Pertea M, Allen JE, Orvis J, et al. Automated eukaryotic gene structure annotation using EVidenceModeler and the Program to Assemble Spliced Alignments. Genome Biology. 2008;9 1:R7. doi:10.1186/gb-2008-9-1-r7.

51. Eddy SR. Accelerated Profile HMM Searches. PLOS Computational Biology. 2011;7 10.

52. Conesa A and Gotz S. Blast2GO: A Comprehensive Suite for Functional Analysis in Plant Genomics. International Journal of Plant Genomics. 2008;2008:619832.

53. Moriya Y, Itoh M, Okuda S, Yoshizawa AC and Kanehisa M. KAAS: an automatic genome annotation and pathway reconstruction server. Nucleic Acids Research. 2007;35:182-5.

54. Yin Y, Mao X, Yang J, Chen X, Mao F and Xu Y. dbCAN: a web resource for automated carbohydrate-active enzyme annotation. Nucleic Acids Research. 2012;40:445-51.

55. Li L, Stoeckert CJ, Jr. and Roos DS. OrthoMCL: identification of ortholog groups for eukaryotic genomes. Genome Research. 2003;13 9:2178-89. doi:10.1101/gr.1224503.

56. Katoh K and Standley DM. MAFFT multiple sequence alignment software version 7: improvements in performance and usability. Molecular Biology Evolution. 2013;30 4:772-80. doi:10.1093/molbev/mst010.

57. Talavera G and Castresana J. Improvement of phylogenies after removing divergent and ambiguously aligned blocks from protein sequence alignments. Systematic Biology. 2007;56 4:564-77. doi:10.1080/10635150701472164.

58. Stamatakis A. RAxML version 8: a tool for phylogenetic analysis and post-analysis of large phylogenies. Bioinformatics. 2014;30 9:1312-3. doi:10.1093/bioinformatics/btu033.

59. Yang Z. PAML: a program package for phylogenetic analysis by maximum likelihood. Computer Application in the Biosciences. 1997;13 5:555-6.

60. Hedges SB, Dudley JT and Kumar S. TimeTree: a public knowledge-base of divergence times among organisms. Bioinformatics. 2006;22 23:2971-2.

61. Drummond AJ and Rambaut A. BEAST: Bayesian evolutionary analysis by sampling trees. BMC Evolutionary Biology. 2007;7:214. doi:10.1186/1471-2148-7-214.

62. Simao FA, Waterhouse RM, Ioannidis P, Kriventseva EV and Zdobnov EM. BUSCO: assessing genome assembly and annotation completeness with single-copy orthologs. Bioinformatics. 2015;31 19:3210-2. doi:10.1093/bioinformatics/btv351.

63. Kent WJ. BLAT--the BLAST-like alignment tool. Genome Research. 2002;12 4:656-64. doi:10.1101/gr.229202.

64. Gurevich A, Saveliev V, Vyahhi N and Tesler G. QUAST: quality assessment tool for genome assemblies. Bioinformatics. 2013;29 8:1072-5. doi:10.1093/bioinformatics/btt086.

65. Initiative AG. Analysis of the genome sequence of the flowering plant *Arabidopsis thaliana*. Nature. 2000;408 6814:796-815.

66. Wu H, Zhang Z, Wang J, Oh D, Dassanayake M, Liu B, et al. Insights into salt tolerance from the genome of *Thellungiella salsuginea*. Proceedings of the National Academy of Sciences of the United States of America. 2012;109 30:12219-24.

67. Liu S, Liu Y, Yang X, Tong C, Edwards D, Parkin IAP, et al. The *Brassica oleracea* genome reveals the asymmetrical evolution of polyploid genomes. Nature Communications. 2014;5 3930:3930-.

68. Hu TT, Pattyn P, Bakker EG, Cao J, Cheng JF, Clark RM, et al. The *Arabidopsis lyrata* genome sequence and the basis of rapid genome size change. Nature Genetics. 2011;43 5:476-81.

69. Sun F, Fan G, Hu Q, Zhou Y, Guan M, Tong C, et al. The high‐ quality genome of *Brassica napus* cultivar ‘ZS11’ reveals the introgression history in semi‐ winter morphotype. Plant Journal. 2017;92 3:452-68.

70. Xu Q, Chen LL, Ruan X, Chen D, Zhu A, Chen C, et al. The draft genome of sweet orange (*Citrus sinensis*). Nature Genetics. 2013;45 1:59-66. doi:10.1038/ng.2472.

71. Argout X, Salse J, Aury JM, Guiltinan MJ, Droc G, Gouzy J, et al. The genome of *Theobroma cacao*. Nature Genetetics. 2011;43 2:101-8. doi:10.1038/ng.736.

72. Teh BT, Lim K, Yong CH, Ng CCY, Rao SR, Rajasegaran V, et al. The draft genome of tropical fruit durian (*Durio zibethinus*). Nature Genetics. 2017;49 11:1633-41. doi:10.1038/ng.3972.

73. Wang K, Wang Z, Li F, Ye W, Wang J, Song G, et al. The draft genome of a diploid cotton *Gossypium raimondii*. Natature Genetics. 2012;44 10:1098-103. doi:10.1038/ng.2371.

74. Zhou Y, Gao S, Zhang X, Gao H, Hu Q, Song Y, et al. Morphology and biochemical characteristics of pistils in the staminate flowers of yellow horn during selective abortion. Australian Journal of Botany. 2012;60 2:143-53.

75. Liang Y, Tan Z, Zhu L, Niu Q, Zhou J, Li M, et al. MYB97, MYB101 and MYB120 Function as Male Factors That Control Pollen Tube-Synergid Interaction in *Arabidopsis thaliana* Fertilization. PLOS Genetics. 2013;9 11.

76. Meng D, He M, Bai Y, Xu H, Dandekar AM, Fei Z, et al. Decreased sorbitol synthesis leads to abnormal stamen development and reduced pollen tube growth via an MYB transcription factor, MdMYB39L, in apple (*Malus domestica*). New Phytologist. 2018;217 2:641-56.

77. Rabiger DS and Drews GN. MYB64 and MYB119 Are Required for Cellularization and Differentiation during Female Gametogenesis in *Arabidopsis thaliana*. PLOS Genetics. 2013;9 9.

78. Sprunck S, Rademacher S, Vogler F, Gheyselinck J, Grossniklaus U and Dresselhaus T. Egg Cell–Secreted EC1 Triggers Sperm Cell Activation During Double Fertilization. Science. 2012;338 6110:1093-7.

79. Samuel MA, Chong YT, Haasen KE, Aldeabrydges MG, Stone SL and Goring DR. Cellular Pathways Regulating Responses to Compatible and Self-Incompatible Pollen in *Brassica* and *Arabidopsis* Stigmas Intersect at Exo70A1, a Putative Component of the Exocyst Complex. The Plant Cell. 2009;21 9:2655-71.

80. Kulich I, Cole RA, Drdova E, Cvrckova F, Soukup A, Fowler JE, et al. *Arabidopsis* exocyst subunits SEC8 and EXO70A1 and exocyst interactor ROH1 are involved in the localized deposition of seed coat pectin. New Phytologist. 2010;188 2:615-25.

81. Kang IH, Steffen JG, Portereiko MF, Lloyd A and Drews GN. The AGL62 MADS Domain Protein Regulates Cellularization during Endosperm Development in *Arabidopsis*. The Plant Cell. 2008;20 3:635-47.

82. Savadi S. Molecular regulation of seed development and strategies for engineering seed size in crop plants. Plant Growth Regulation. 2018;84 3:401-22.

83. Galego L and Almeida J. Role of DIVARICATA in the control of dorsoventral asymmetry in *Antirrhinum* flowers. Genes & Development. 2002;16 7:880-91.

84. Liang Q, Li H, Li S, Yuan F, Sun J, Duan Q et al. Supporting data for "The genome assembly and annotation of yellowhorn (Xanthoceras sorbifolium Bunge)" GigaScience Database 2019. http://dx.doi.org/10.5524/100589

85 Bi Q, Zhao Y, Du W, Lu Y, Gui L, Zheng Z et al. Pseudomolecule-level assembly of the Chinese oil tree yellowhorn (Xanthoceras sorbifolium) genome. GigaScience 2019 [PLEASE INSERT DOI BEFORE PUBLICATION]

Click here to access/download;Table;Table 1.docx

Table 1. Statistics of Illumina, 10X Genomics, and Hi-C sequencing data

Platform	Library type	Read length (bp)	No. of raw reads (Mb)	Reads retained after trimming (Mb)	Total valid base (Gbp)
Illumina	280 bp Size	150	451.34	439.84	65.98
	450 bp Size	150	696.88	658.72	98.81
10x Genomics	350 bp Size	150	457.40	457.40	63.35
Hi-C	600 bp Size	150	932.76	891.72	133.76

 Click here to access/download;Table;Table 2.docx

Table 2. Statistics of PacBio Sequal sequencing data

Index	PacBio
Total Number of reads	7,062,244
Mean length of raw reads (bp)	226,712
N50 of raw reads (bp)	374,500
Mean length of subreads (bp)	156,717
N50 of subreads (bp)	237,539
Coverage (X)*	160.51

*Coverage (X) = (read count * read length) / estimated genome size.

Table 3. Summary of yellowhorn genome assembly.

Statistics	Contig	Contig (polished)	10X Genomics	BioNano	Hi-C	
					Scaffold	Chromosome
Total number	2,002	2,002	707	29	267	15
Total length (bp)	505,787,109	508,445,799	513,924,146	461,662,473	439,965,977	419,835,445
N50 length (bp)	642,338	645,453	2,334,658	29,979,918	29,432,808	29,432,808
N90 length (bp)	113,799	114,103	492,748	15,941,042	17,893,618	17,893,618
Max length (bp)	4,375,484	4,395,303	21,312,255	75,772,594	39,123,600	39,123,600
GC content (%)	35.25	35.13	34.67	32.39	32.76	34.18

Table 4. BUSCO assessment of yellowhorn genome.

Description		yellowhorn	
		Number	Percentage (%)
Complete BUSCOs (C)	Complete and single-copy BUSCOs (S)	1,175	81.60
	Complete and duplicated BUSCOs (D)	43	2.98
Fragmented BUSCOs (F)		23	1.60
Missing BUSCOs (M)		199	13.82
Total BUSCO groups		1,440	100

Table 5. Repeat content of yellowhorn genome assembly Click here to access/download;Table;Table 5.docx

Table 5. Repeat content of yellowhorn genome assembly.

Term		Length (bp)	Percentage of genome (%)
DNAs*	DNA	374,909	0.09
	DNA/CMC-EnSpm	1,699,637	0.39
	DNA/MuLE-MuDR	3,896,024	0.89
	DNA/PIF-Harbinger	1,104,979	0.25
	DNA/TcMar-Pogo	94,067	0.02
	DNA/hAT-Ac	4,103,980	0.93
	DNA/hAT-Tag1	890,950	0.20
	DNA/hAT-Tip100	1,213,576	0.28
SINEs*	SINE	343	0.00
	SINE/tRNA	10,674	0.00
LINE*	LINE/L1	16,861,661	3.83
LTRs*	LTR	2,861	0.00
	LTR/Caulimovirus	1,360,538	0.31
	LTR/Copia	52,384,264	11.91
	LTR/Gypsy	51,370,228	11.68
	LTR/Pao	88	0.00
Low_complexity		1,516,978	0.34
RC*		4,215	0.00
RC/Helitron		5,949	0.00
rRNA		64,618	0.01
SSRs		6,971,711	1.58
Unknown		104,792,508	23.76
Total		248,724,758	56.39
Genome size		439,965,977	100.00

*DNA: DNA transposons; LINE: long interspersed nuclear elements; SINE: short interspersed nuclear elements; LTR: long terminal repeat; RC: rolling circle replication. SSRs: Simple sequence repeats.

Figure 1. Morphological characteristic of yellowhorn superior 'WF18'

Click here to access/download;Figure;Figure 1.tif

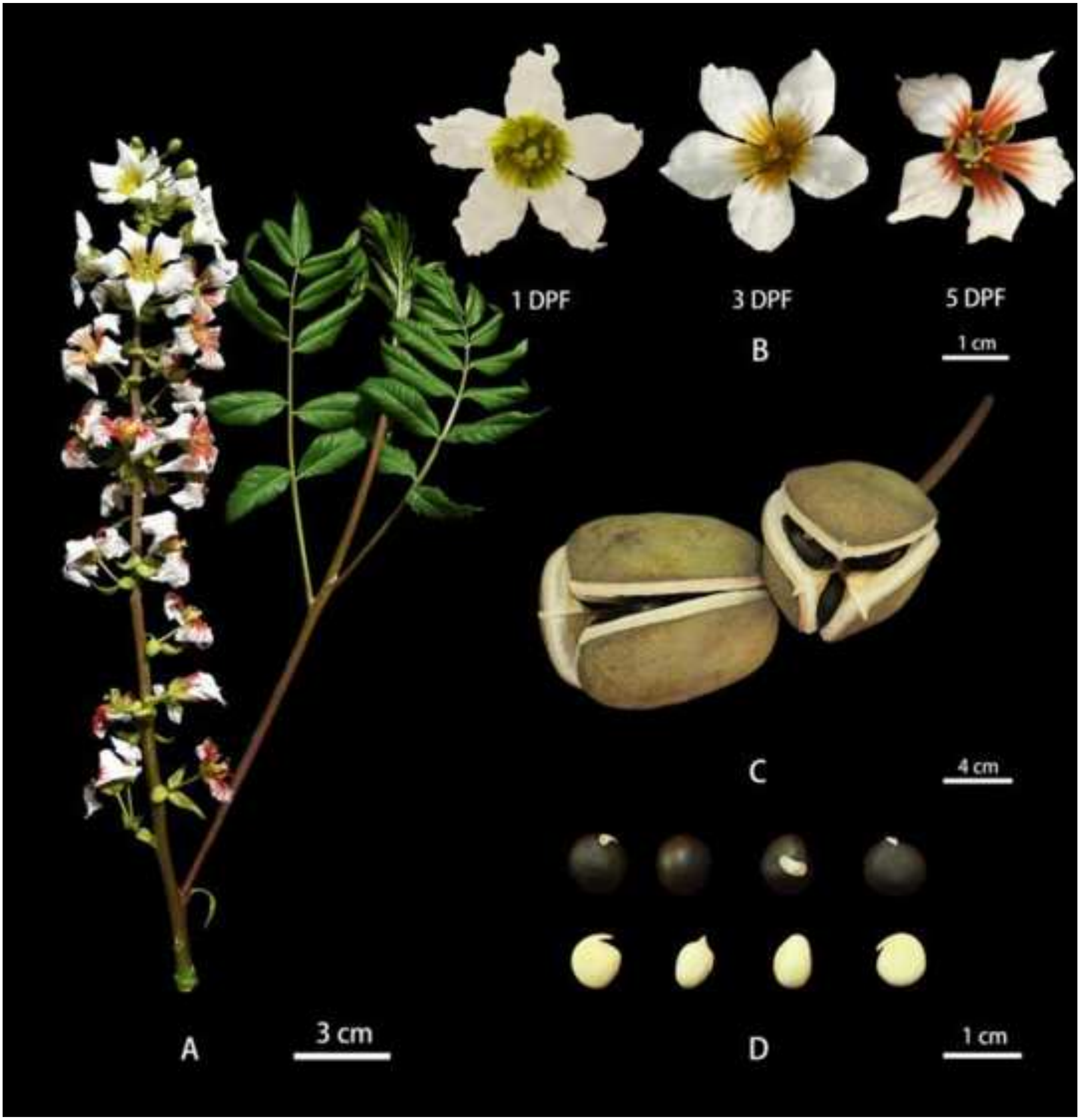

Figure 2. Flowchart of genome assembly and annotation

Click here to access/download;Figure;Figure 2.tif

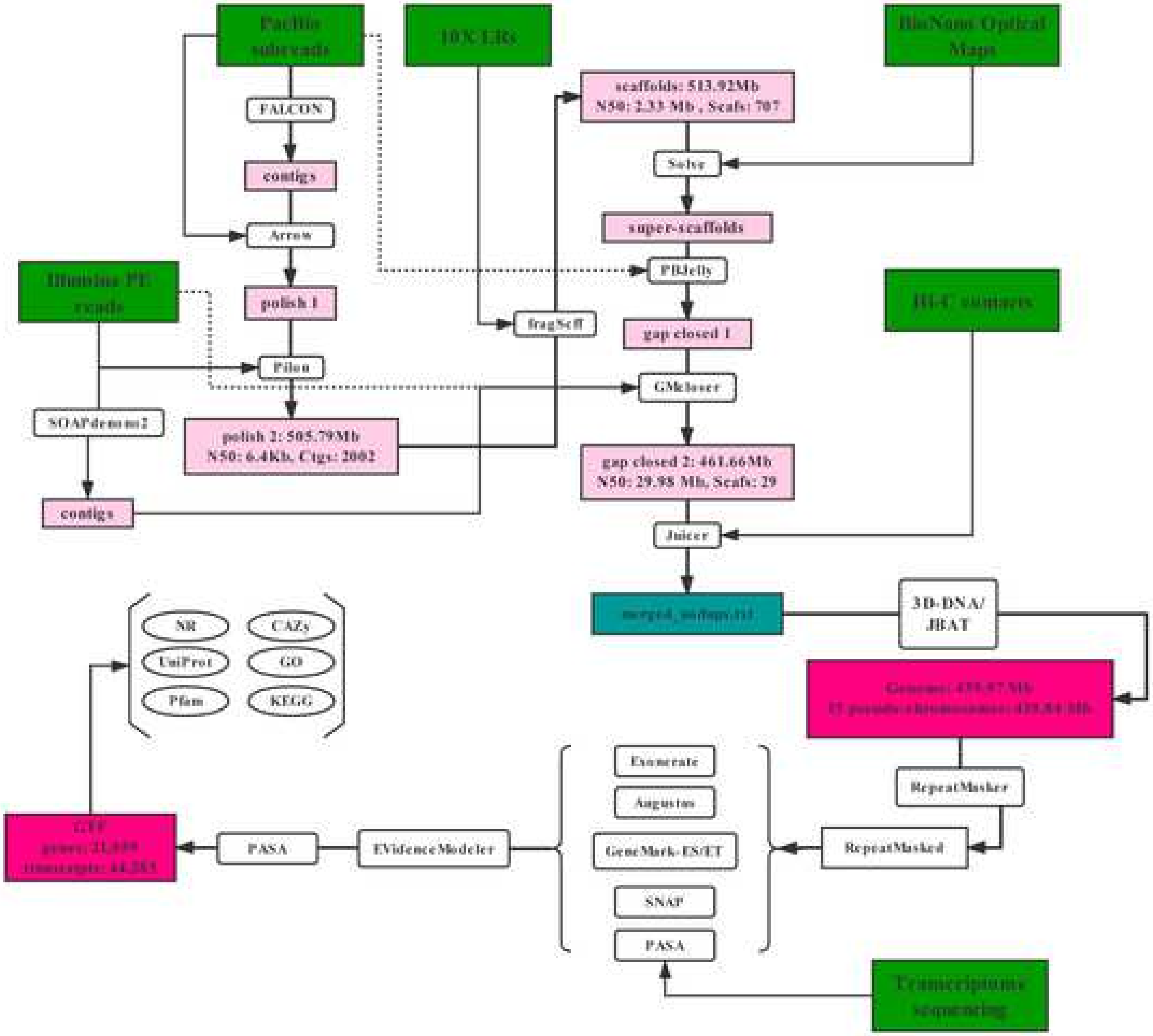

Figure 3. Contact maps of Hi-C links among chromosomes.

Click here to access/download;Figure;Figure 3.tif

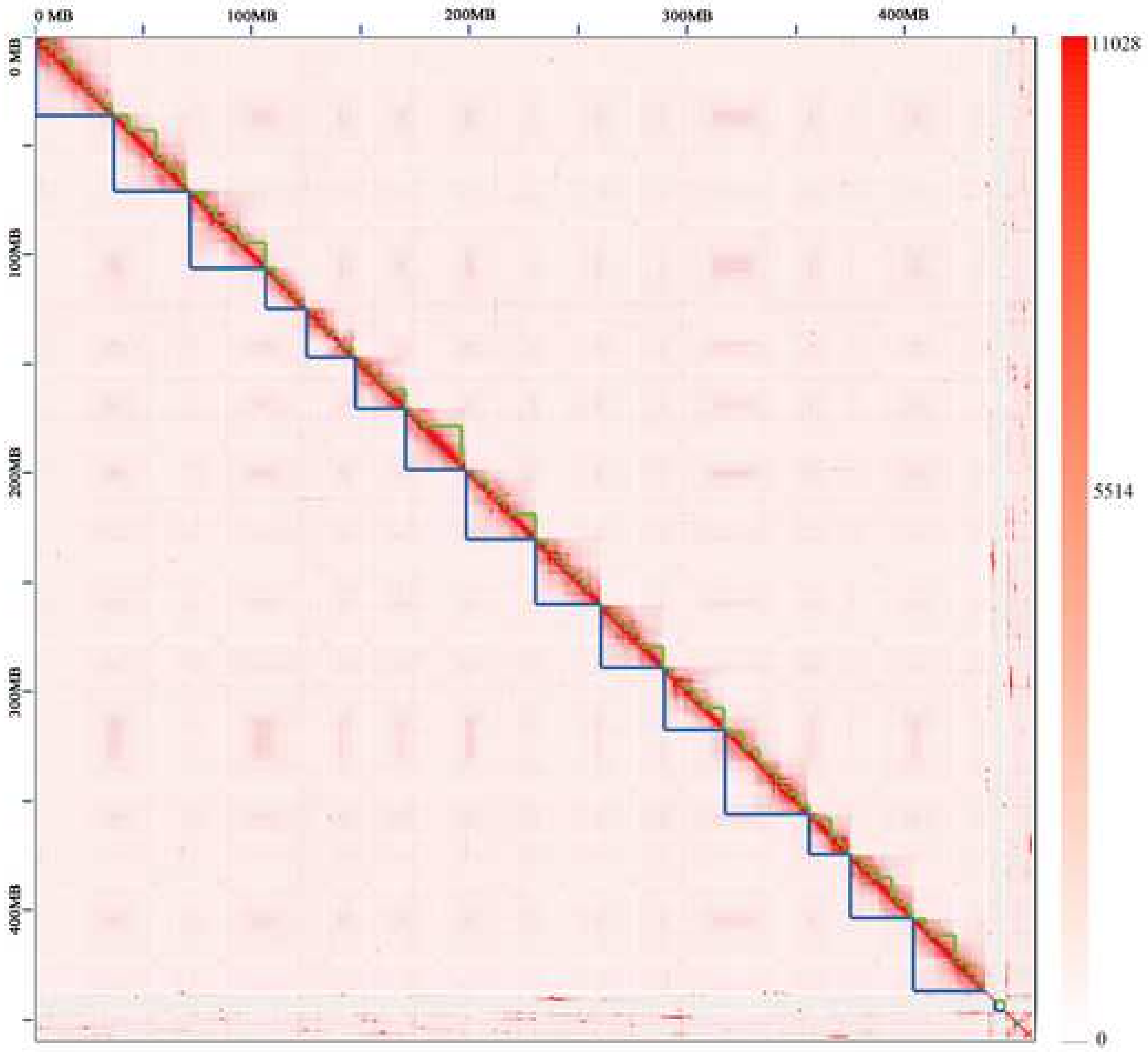

Figure 4. Yellowhorn genome features.

Click here to access/download;Figure;Figure 4.tif

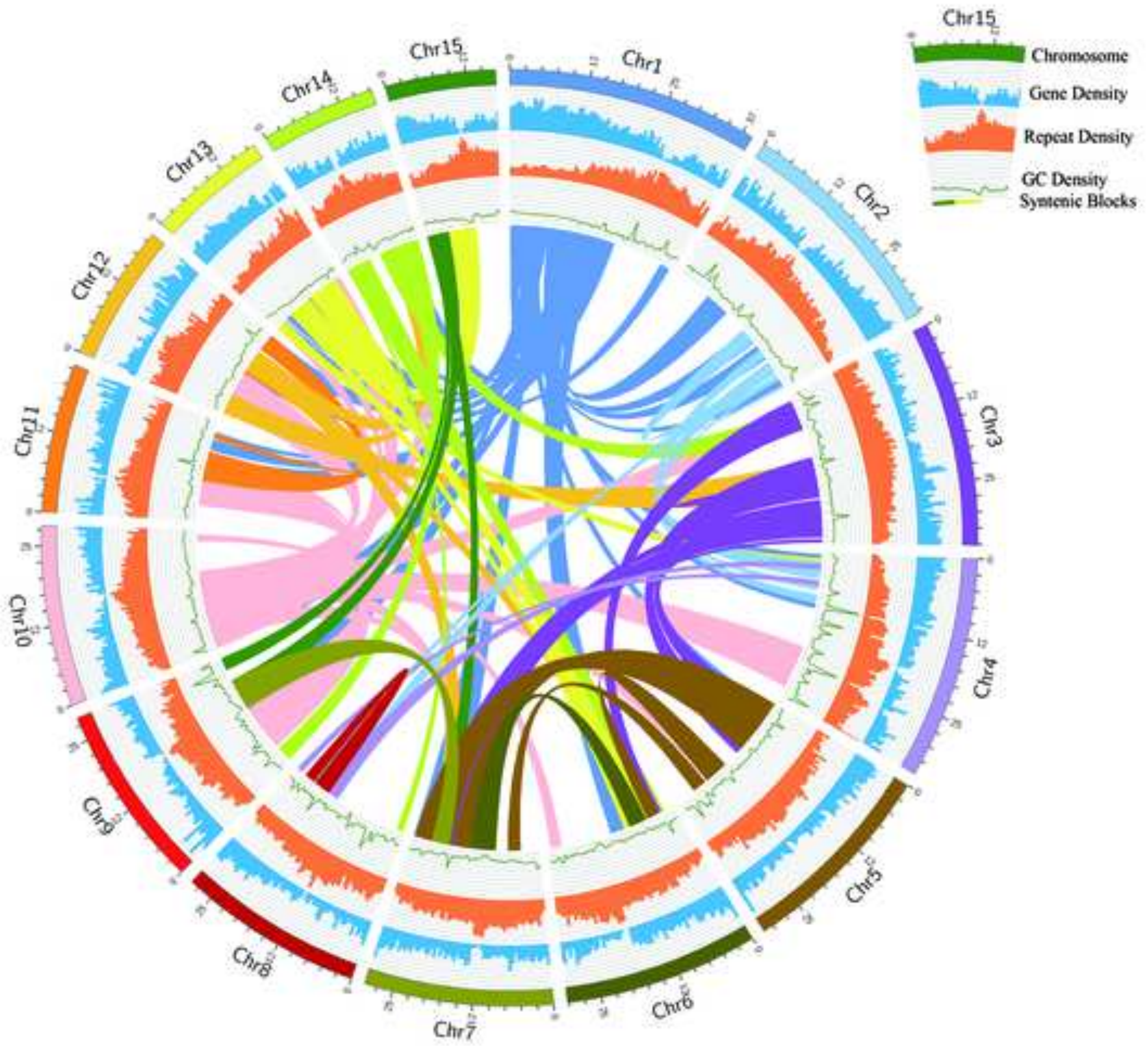

Figure 5. Phylogenomics analysis of yellowhorn genome.

Click here to access/download;Figure;Figure 5.tif

A

B

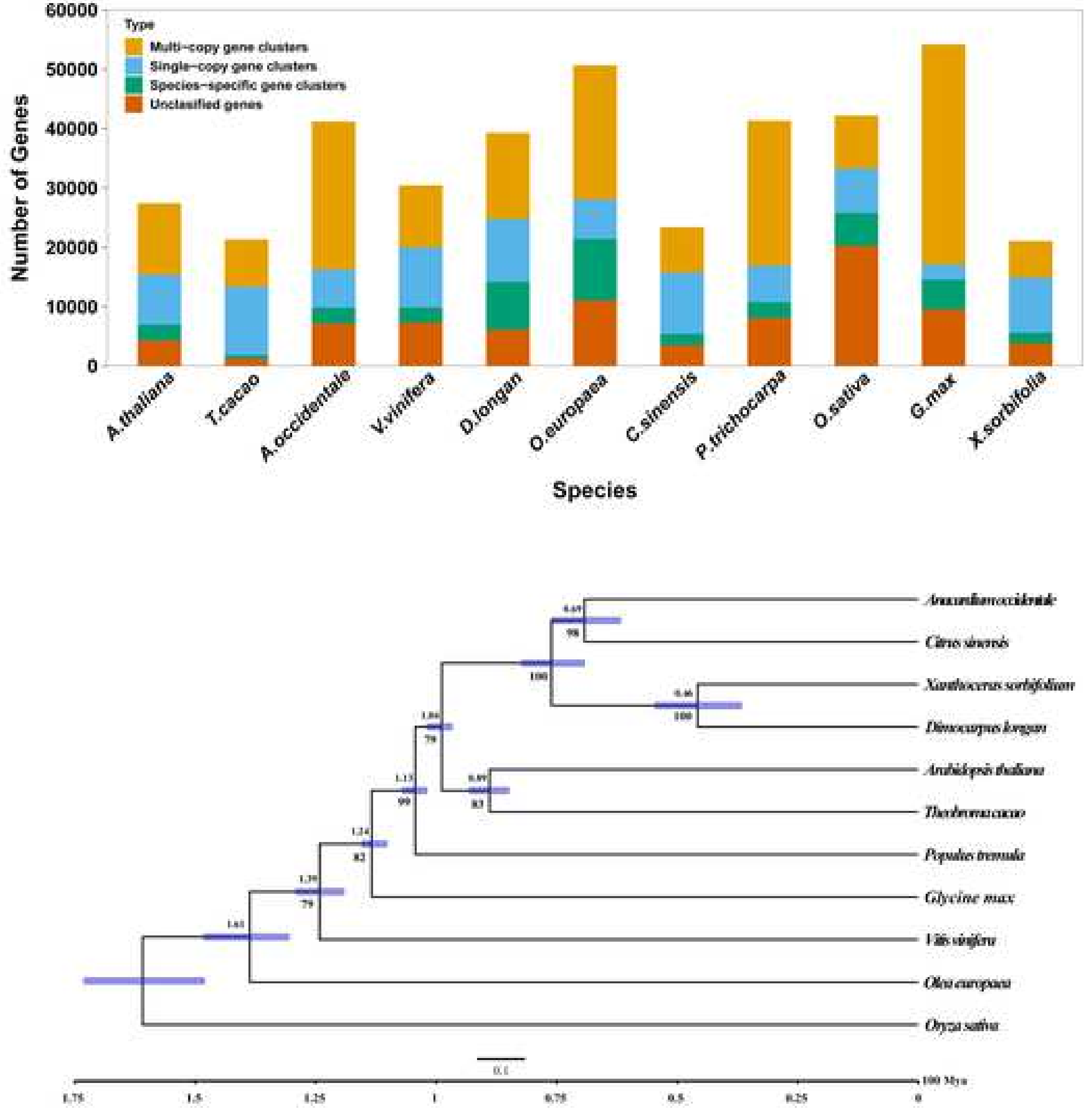

Figure 6. Tissue-specific gene analysis.

Click here to access/download;Figure;Figure 6.tif

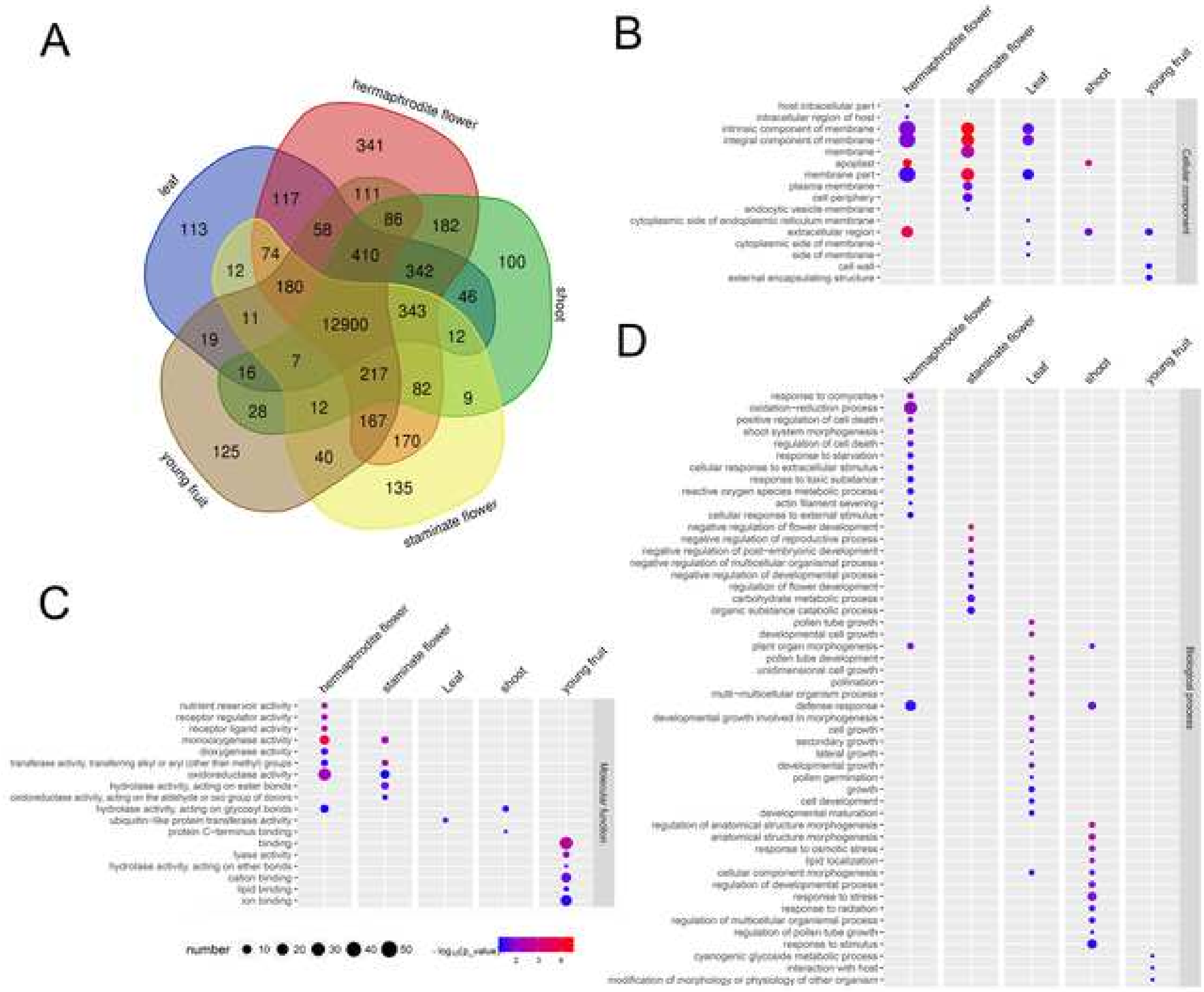

Table S1. Statistics of transcriptome sequencing data.

Click here to access/download
Supplementary Material
Table S1.xlsx

Table S2. The features of yellowhorn genome assembly.

Click here to access/download
Supplementary Material
Table S2.xlsx

Table S3. Genome QC report of yellowhorn genome assembly by QUAST.

Click here to access/download
Supplementary Material
Table S3.xlsx

Table S4. GO enrichment of yellowhorn specific genes.

Click here to access/download
Supplementary Material
Table S4.xlsx

Table S5. The 195 single copy orthologous genes in yellowhorn genome assembly and other ten species.

Click here to access/download
Supplementary Material
Table S5.xlsx

Table S6 Yellowhorn tissues-specific genes.

Click here to access/download

Supplementary Material

Table S6.xlsx

Table S7. GO enrichment of yellowhorn tissues-specific genes.

Click here to access/download
Supplementary Material
Table S7.xlsx

Figure S1. Karyogram of yellowhorn superior 'WF18'.

Click here to access/download
Supplementary Material
Figure S1.tif

Figure S2. Yellowhorn genome evaluation and estimation by GenomeScope.

Click here to access/download
Supplementary Material
Figure S2.tif
