# Supplementary figures and images for: The genome assembly and annotation of yellowhorn (Xanthoceras sorbifolium Bunge)
Source: Gigascience. 2019 Jun 26;8(6):giz071. doi: 10.1093/gigascience/giz071 (PMC6593362; doi:10.1093/gigascience/giz071)

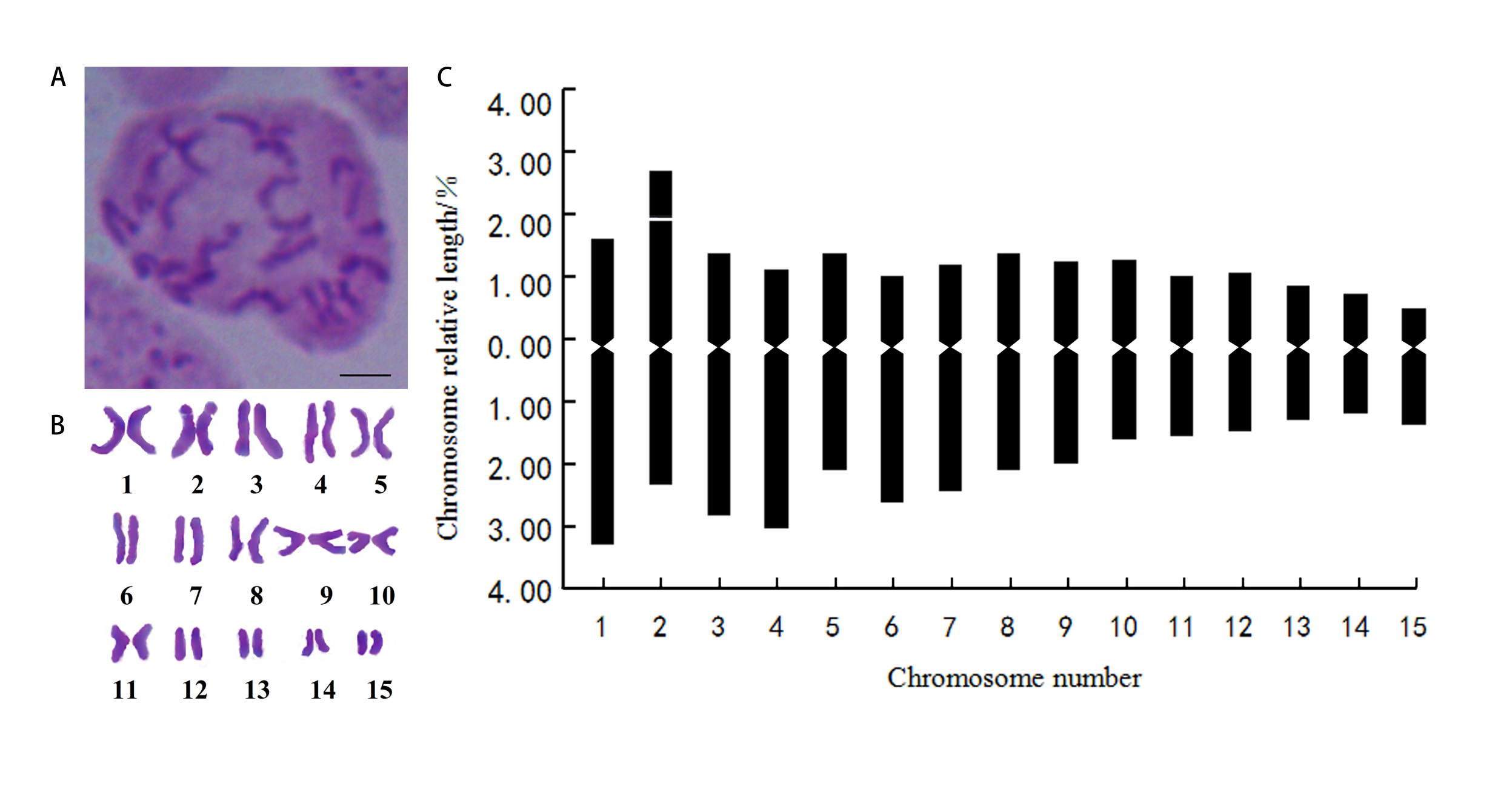

Supplement: giz071_Supplemental_Files [file giz071_supplemental_files.zip › Figure S1.tif]

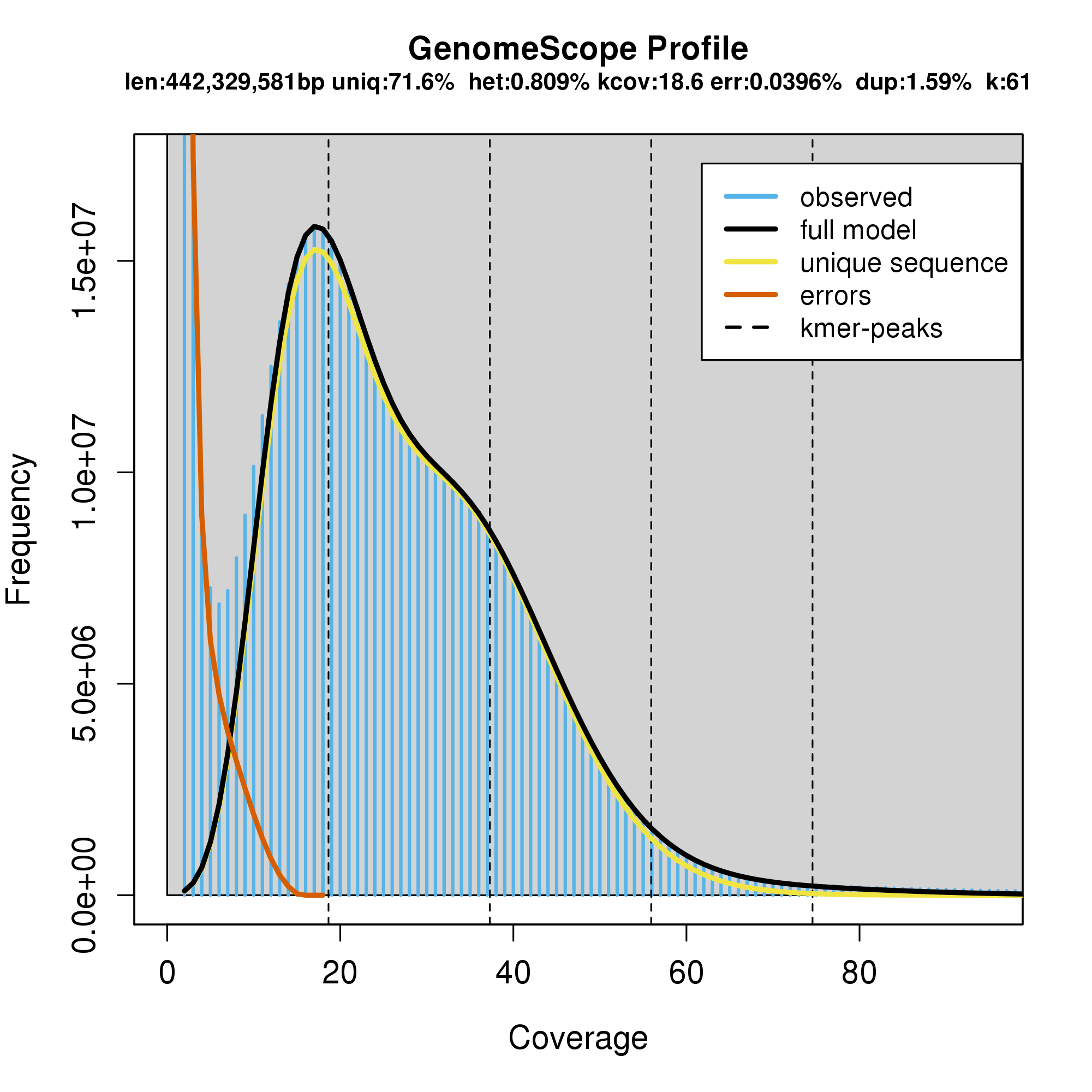

Supplement: giz071_Supplemental_Files [file giz071_supplemental_files.zip › Figure S2.tif]
