# Supplementary material for: The genome assembly and annotation of yellowhorn (Xanthoceras sorbifolium Bunge)
Source: Gigascience. 2019 Jun 26;8(6):giz071. doi: 10.1093/gigascience/giz071 (PMC6593362; doi:10.1093/gigascience/giz071)
Supplement: giz071_Response_to_Reviewer_Comments_Revision_1 [file giz071_response_to_reviewer_comments_revision_1.pdf]

Author's Response To Reviewer Comments

Close

Dear Dr. Zauner,
Thanks for your comments and suggestions on our revised version of the manuscript entitled “The genome assembly and annotation of yellowhorn (Xanthoceras sorbifolium Bunge)" (GIGA-D-18-00410R1). We have carefully revised the manuscript according to reviewer’s comments. Meanwhile, we have checked and confirmed our GigaDB dataset according to the suggestions of data curators. Also, we have cited the DOI link: http://dx.doi.org/10.5524/100589 in our revised manuscript.
We are very happy that this article can be published in GigaScience. Thank you again!
If you have any questions, please do not hesitate to contact me.
Sincerely yours,
Ke Qiang Yang
Response to the comments
Reviewer #2: The Authors appear to have gone to a great deal of effort to address all of the points I raised previously and I am satisfied with the changes they have made to the manuscript.
Authors’ response: We would like to sincerely thank reviewer for the valuable and helpful comments and suggestions about our manuscript.
I only have a few very minor additional suggested changes, relating to typographical errors:
Page 1, line 25: Change "recently common ancestral" to "recent common ancestor".
Authors’ response: Revised as required.
Page 3, line 77: Change "suquencing" to "sequencing".
Authors’ response: Revised as required.
Page 5, line 120: Change "agilned" to "aligned".
Authors’ response: Revised as required.
Page 8, line 209: Change "origent" to "orientate".
Authors’ response: Revised as required.
Page 9, line 256: Change "contained" to "containing".
Authors’ response: Revised as required.
Page 12, line 356: Change "leat" to "least".
Authors’ response: Revised as required.
Page 14, line 412: Change "represnts" to "represents".
Authors’ response: Revised as required.
Table 4: Correct spelling of "yellowgorn" to "yellowhorn". [this was also flagged up in my initial review]
Authors’ response: As suggested, we rectified the spelling mistakes, and also modified the format of Table 4 in revised manuscript.

Close
