# Supplementary material for: The genome assembly and annotation of yellowhorn (Xanthoceras sorbifolium Bunge)
Source: Gigascience. 2019 Jun 26;8(6):giz071. doi: 10.1093/gigascience/giz071 (PMC6593362; doi:10.1093/gigascience/giz071)
Supplement: giz071_Reviewer_1_Report_Original_Submission — John Mackay, Ph.D. -- 11/10/2018 Reviewed [file giz071_reviewer_1_report_original_submission.pdf]

Reviewer Report

Title: The genome assembly and annotation of yellowhorn (Xanthoceras sorbifolium Bunge)

Version: Original Submission **Date:** 11/10/2018

Reviewer name: John Mackay, Ph.D.

Reviewer Comments to Author:

The report by Qiang Liang and colleagues describes the genome assembly and first analysis of the shrub Yellowhorn. Much of the work is concerned with producing an assembly of the genome. For this purpose, data were generated by using several methodologies and several successive steps were followed to produce and validate an assembly comprised of 15 pseudo-molecules.
The main strength of the report is the extensive nature and different types of the data that were developed and the systematic approach that was followed to produce the high-level assembly as set out in the aims. Each of the methods are relatively well established but few studies that I have encountered have used such a highly integrated process combining such an array of methods. The use of optimal mapping and Hi-C in addition to very deep coverage in both long and short sequence reads enable the chromosome level assembly without having a prior physical map of the genome. The main conclusions as to the quality of the assessment of the assembly are well supported by the data shown.
The main weakness of the paper concerns the transcript profiling data. It was not clear if or how they RNA-Seq data were used in validating the gene models in any way. Furthermore, the RNA-Seq were analysed to identify sets of tissue specific genes, which were analysed by using a GO term enrichment approach but it is unclear if or how this is useful or adds any biological information relevant to the species or its genome.
The phylogenetic analysis conclusions appeared to be well supported by the data but the resolution of Fig. 5 was too loo to read the axis labels and bootstrap values so it was not possible to determine the statistical support of tree.
Figure 2 is useful as it clearly shows the steps that were followed during the assembly process. It would be more informative if it also contained some of the key statistics or data from the major steps to illustrate how the work proceeded.
The manuscript is well written in standard English and contained very few typos.

Level of Interest

Please indicate how interesting you found the manuscript: Choose an item.

Quality of Written English

Please indicate the quality of language in the manuscript: Choose an item.

Declaration of Competing Interests

Please complete a declaration of competing interests, considering the following questions:

- Have you in the past five years received reimbursements, fees, funding, or salary from an organisation that may in any way gain or lose financially from the publication of this manuscript, either now or in the future?
- Do you hold any stocks or shares in an organisation that may in any way gain or lose financially from the publication of this manuscript, either now or in the future?
- Do you hold or are you currently applying for any patents relating to the content of the manuscript?
- Have you received reimbursements, fees, funding, or salary from an organization that holds or has applied for patents relating to the content of the manuscript?
- Do you have any other financial competing interests?
- Do you have any non-financial competing interests in relation to this paper?

If you can answer no to all of the above, write 'I declare that I have no competing interests' below. If your reply is yes to any, please give details below.

I declare that I have no competing interests

I agree to the open peer review policy of the journal. I understand that my name will be included on my report to the authors and, if the manuscript is accepted for publication, my named report including any attachments I upload will be posted on the website along with the authors' responses. I agree for my report to be made available under an Open Access Creative Commons CC-BY license (http://creativecommons.org/licenses/by/4.0/). I understand that any comments which I do not wish to be included in my named report can be included as confidential comments to the editors, which will not be published.

I agree to the open peer review policy of the journal

To further support our reviewers, we have joined with Publons, where you can gain additional credit to further highlight your hard work (see: https://publons.com/journal/530/gigascience). On publication of this paper, your review will be automatically added to Publons, you can then choose whether or not to claim your Publons credit. I understand this statement.

Yes Choose an item.
