# Supplementary material for: The genome assembly and annotation of yellowhorn (Xanthoceras sorbifolium Bunge)
Source: Gigascience. 2019 Jun 26;8(6):giz071. doi: 10.1093/gigascience/giz071 (PMC6593362; doi:10.1093/gigascience/giz071)
Supplement: giz071_Reviewer_2_Report_Original_Submission — Laura Kelly -- 11/24/2018 Reviewed [file giz071_reviewer_2_report_original_submission.pdf]

Reviewer Report

Title: The genome assembly and annotation of yellowhorn (Xanthoceras sorbifolium Bunge)

Version: Original Submission **Date:** 11/24/2018

Reviewer name: Laura Kelly

Reviewer Comments to Author:

This paper reports the whole genome sequence of Xanthoceras sorbifolium (yellowhorn), an economically important species that is used for oil production and as a source of medicinal compounds. Several different sequencing technologies are used to assemble the genome and generate pseudochromosomes; the final assembly is highly contiguous and annotated for both repeats and protein coding genes.
However, there are several aspects of the manuscript that require improvements or clarification, which I outline below. One particular issue that needs attention throughout the manuscript is the lack of detail about the specific parameter settings for the majority of software used to perform the analyses.
Abstract
Page 1, line 11: Change "great economic values" to "great economic value".
Page 2, lines 23-24: Suggest rephrasing "yellowhorn diverged from the common ancestral of Dimocarpus longan" to "yellowhorn and Dimocarpus longan diverged from their most recent common ancestor".
Background
Page 2, line 34: I don't understand what the Authors mean when they say that yellowhorn is resistant to "leanness".
Page 2, line 35: Change "pharmacological values" to "pharmacological value".
â€¨
Page 2, line 40: Change "possess the antitumor" to "possess antitumor".
Page 2, line 41: Suggest changing "the potentiality against Alzheimer's disease" to "potentiality activity against Alzheimer's disease".
Page 3, line 52: Change "Functionally annotation" to "Functional annotation".
Page 3, line 54: Change "facilitate to comparative genomics" to "facilitate comparative genomics".
Sampling and sequencing
Was a voucher specimen of the sequenced individual made? If so, please provide details of the specimen (e.g. collector's number) and state the herbarium or other collection in which it is lodged.
Page 3, line 66: Correct spelling to "Illimina" to "Illumina"; this same error also occurs elsewhere in the text, so all instances need correcting.
Page 4, line 67: Please state which version of FASTQC was used.
Page 4, line 69: The estimated genome coverage from the cleaned Illumina reads is stated, but the genome size of yellowhorn has not yet been mentioned.
Page 4, line 79: "After reads trimming"; please make clear whether the reads from the 10X Genomics library were trimmed in the same way as reported above for the Illumina short insert libraries. If not, please provide specific details of how these reads were trimmed.

Transcriptome sequencing
Page 5, line 99: Please clarify whether entire flowers were used for the "hermaphrodite flower" and "staminate flower" tissues. â€¨
Page 5, line 106: Please specify the details of parameter settings used for Trimmomatic; if the default settings were used this should be stated.
Genome assembly by PacBio long reads
Page 5, line 109: Please specify the details of parameter settings used for kmergenie.
The plot of kmer sizes in Fig. S1a, from which the "best" kmer size was selected, does not appear to have reached a clear maximum. According to the kmergenie website "the plot should be roughly concave and have a clear global maximum. If not, the predicted best k is likely to be inaccurate". As the plot in Fig. S1 does not match this description (nor does it look like the examples given on the kmergenie website) I am doubtful whether the estimate of the best kmer size is accurate, and therefore the resulting genome size (GS) estimate may also be inaccurate. Could the Authors report the maximum kmer size they tested and explain why their plot does not have a clear global maximum? Moreover, could they address the issue of the accuracy of the GS estimate derived from this method? It would be a great advantage if GS could also be estimated via an independent approach, ideally by using flow cytometry.
Also, although the peaks for kmers overlapping homozygous and heterozygous positions in the genome are identified in the legend for Fig S1, there is no mention of heterozygosity anywhere in the text. It would be useful if an estimate of the % heterozygosity from kmer analysis was reported in the text, as the level of heterozygosity can have an important impact on genome assembly.
Page 6, line 111: It is very good that a karyotype analysis has been performed and the chromosome number of yellowhorn confirmed. However, there are no details of the experimental methods used to perform this analysis. The Authors need to provide these.
Page 6, line 114: Please specify what parameter settings/thresholds were used when performing error correction, other than the length cut-offs that are mentioned in the following lines.
Page 6, lines 124-127: Please give details of any specific parameter settings used for Pbalign, Arrow, BWA and pilon.
Pseudo-chromosomes construction using 10X Genomics, BioNano optical maps and Hi-C â€¨
Page 6, line 131: Please specify what parameter settings when mapping the linked-reads with BWA. Given that evidence of heterozygosity was detected by the kmer analysis (see comment above), I wonder if any information on haplotypes was obtained from the linked-reads?
Page 7, line 136: Please state which software was used to perform in the in silico digestion.
Page 7, lines 138-147: Please provide details of specific parameter settings used for each piece of software mentioned in this section.
Page 7, line 141: Should "leading to 6,015 gaps were addressed" read "leading to 6,015 gaps being resolved"?
Page 7, line 144: Change "represented" to "representing".
Page 7, line 145: Should "free duplicate" read "duplicate free"? (also on the following line)
Page 7, line 147: "was used to scaffold splitting, anchor, order, orient, misjoin correction"; this needs rephrasing.
Page 7, lines 150-151: "The results showed that yellowhorn genome assembly was 439.97 Mb". I'm not entirely clear whether some sequence, that wasn't incorporated into the final Hi-C scaffolds, was

discarded. The assembly size was reduced from 508.45 Mb for the PacBio polished contigs, to 439.97 Mb after scaffolding, gap filling, reorientation, etc. Could the Authors clarify whether any sequences that formed part of the PacBio polished contigs were dropped during this process? If so, could the Authors comment on what these sequences were, e.g. organellar sequences, alternate haplotypes from heterozygous regions of the genome etc.â€¨
Genome assemble assessment
I suggest changing the title of this section to "Genome assembly assessment".
Please provide details of specific parameter settings used for each piece of software mentioned in this section.
Page 8, line 160: Should "were closed to those of" read "was close to that of"?
Repeat sequence analysis â€¨
Please provide details of specific parameter settings used for each piece of software mentioned in this section.
Was any pre-masking of the de novo repeat library done for captured gene fragments present within repeats, or high-copy number genes, such as rDNA genes? If not, some of the protein-coding genes in the genome may have been erroneously counted within the repetitive fraction. The Authors need to clarify this point.
Page 8, lines 166-169: The Authors report that the estimated repeat content of the yellowhorn genome is "higher than that of other species of the Malvaceae", but some of the species that are being compared with yellowhorn (i.e. Citrus sinensis and Dimocarpus longan) are not in the Malvaceae. Also, I'm not clear what the rationale is for specifically comparing the repeat content of yellowhorn with species from the Malvaceae; why not also compare it with species from the Brassicaceae, which are equally closely related? The Authors need to explain the reasoning behind their comparison.
Also, the Authors should make clear that they have estimated the percentage of repetitive DNA in the genome assembly, i.e. the amount of repetitive DNA is expressed as a percentage of the assembly size, not the genome size. If the assembly is incomplete then the percentage of repetitive DNA in the actual genome may be different.
Genome annotation
Please provide details of specific parameter settings used for each piece of software mentioned in this section.
Page 8, lines 174-175: "The obtained ORFs were used for training ab initio predictors on repeat-masked genome". If a hard-masked version of the genome assembly is being used here, and the repeat masking did not account for high copy numbers genes and the possibility of captured gene fragments (see comment in section above), then some protein coding genes may be missed because they have been erroneously masked as repeats. This could lead to an underestimation of the number of genes within the X. sorbifolium genome.
Page 9, line 178: "To predict homology genes"; should really read "To predict genes based on similarity".
Page 9, lines 178-180: The Authors list a number of plant species whose proteins were used to aid gene prediction. Although there is a list of URLs at the end of the manuscript that indicates where these data were obtained from, this isn't actually referred to here. Also, the Authors need to state the exact versions of assemblies and annotations that were used for each of the species. Also, full species names should be given upon their first mention in the text; I don't think the full name for A. occidentale is given

anywhere.
Page 9, lines 186-187: Please provide details of any parameter settings used for the database search, and also give references for the databases.
Comparative phylogenomics â€¨
Page 9, lines 191-192: Please specify what software was used to filter the protein sequences; if a custom script was used, this should be provided. Also, were organellar sequences checked for and removed from the protein sets?
Page 9, line 193: Please state which version of BLAST was used. Also, change "were used to ortholog by OrthoMCL" to "were used to predict putative orthologs with OrthoMCL". Also, specify any parameter settings used with OrthoMCL (e.g. the inflation parameter setting).
Page 9, lines 193-194: "Orthogroups of 27,347 were constructed, followed by 9,905 species specific groups and 17,442 paralogs"; this needs rephrasing. I don't understand the distinction that is being made between "orthogroups" and "paralogs". It is important to recognise that, even if an OrthoMCL cluster is single copy it does not necessarily mean that all of the sequences in this group are orthologs. Both single and multi-copy "orthogroups" can contain a mixture of orthologous and paralogous sequences.
Page 9, line 196: Please provide more details for the GO enrichment analysis. I.e. which test was used, which algorithm was run and what was used as the background.
Page 10, line 199: "The protein sequences of 198 single copy orthogroups"; please clarify why these 198 groups in particular were selected for phylogenetic analysis. E.g. were these the only groups with a single sequence from each species? Also, as noted above, single-copy clusters from OrthoMCL are not necessarily comprised solely of orthologous sequences, and the combining of orthologs and paralogs may confound phylogenetic inference of species relationships.
Page 10, line 201: Please provide details of the settings used with GBLOCKS.
Page 10, line 204: Change "Divergent time" to "Divergence time".
Page 10, line 206-207: "The evolutionary timescale of O. sativa and A. thaliana was obtained from TimeTree database and was used as calibrate point" would be better written as "The divergence time of O. sativa and A. thaliana was obtained from the TimeTree database and was used as calibration point". Also, please specify the exact value that was used for the calibration and include a reference for the database. Moreover, the use of a secondary calibration point, rather than fossil calibrations, is not ideal because this will likely already have a significant degree of uncertainty (see for example https://doi.org/10.1371/journal.pone.0148228). Another issue is whether the divergence time estimation results are based on a single MCMCtree run, which is not recommended, or whether multiple runs were performed and the results compared to ensure that they converged on similar mean divergence time estimates for each of the nodes. The Authors need to clarify this point.
Page 10, lines 208-209: Suggest rephrasing "suggested that yellowhorn diverged from the common ancestral of D. longan at approximately 58.63 million years ago" to "suggested that yellowhorn and D. longan diverged from their most recent common ancestor approximately 58.63 million years ago". Also, as well as the points raised above regarding use of secondary calibration points and the need to perform multiple runs in MCMCtree, the divergence time estimate for Xanthoceras and Dimocarpus differs significantly from those obtained from other studies (as reported in TimeTree database, all of which come up with much older dates). How can the Authors explain this disparity?

Transcriptome analysis of tissue-specific expression
Please provide details of specific parameter settings used for each piece of software mentioned in this section.
Page 10, line 213: Change "ratio of 75.68%" to "rate of 75.68%".
Page 11, line 224: Change "were the mostly enriched functions" to "were the most enriched functions". Also, this line refers to "Fig. 6", but this figure is missing from the manuscript, so I have not been able to review it.
Discussion
Page 11, line 230: "the other reported species of the Malvaceae family"; as already noted above, some of the species mentioned are not in the Malvaceae.
Page 11, line 231-232: "repeats in yellowhorn genome appeared to have expanded as compared to T. cacao"; how do these assemblies compare in terms of contiguity/completeness and how do the methods of repeat analysis compare? Differences in these aspects could contribute to the apparent difference in repeat content between these two species.
Page 11, line 236-237: "A new Xanthoceraceae family was published to alteration of family limits for Sapindaceae"; this sentence needs rephrasing.
Page 11, line 236-237: "The result of comparative phylogenomics suggested that yellowhorn diverged fromâ€¨the common ancestral of D. longan within Sapindaceae". I cannot see how this conclusion can be drawn, as yellowhorn and D. longan are the only representatives of the Sapindaceae included in the analysis. â€¨
Figure and Table Legend
Figure 4: Please provide details of the software used to generate this figure.
Figure 5: Rephrase "Orthologue clustering"; as commented above, OrthoMCL clusters sequences into groups of putative orthologs and paralogs. Also, change "Phylogenetic tree and divergence time" to "Phylogenetic tree and estimated divergence time". Also, change "numbers beside the branching nodes" to "numbers above the branches". Moreover, more explanation of the labels on the tree is needed, i.e. state what the numbers in brackets represent (credibility intervals?) and explain the scale-bar. Bootstrap support values also need to be added
Table 4: Correct spelling of "yellowgorn" to "yellowhorn".
Reference dataâ€¨URLs
Some of the URLs just point towards a general site (e.g. jgi) and do not link to the specific data used.
Tables
Table 1: The "Library type" and "Insert size" columns are basically redundant and could be combined. Also, I'm not sure it makes sense to include the stats for the BioNano data in this table, because it is not actual sequence data. Remove "(bp)" from the heading for the "No. of reads" column. Also, please double-check all values for total base pairs and reads retained after trimming, because appear to be wrong.
Figures
Figure 5: In part A, change the labels that say "Multi-copy orthologs", "Single-copy orthologs" etc., to "Multi-copy OrthoMCL clusters" or "Multi-copy gene clusters" etc. In part B, bootstrap support values need to be added to the phylogenetic tree. Also, the layout needs to be improved because some of the

labels are on top of branches and cannot be read properly.
Figure 6 is mentioned in the text, but was missing from the manuscript.

Level of Interest

Please indicate how interesting you found the manuscript: Choose an item.

Quality of Written English

Please indicate the quality of language in the manuscript: Choose an item.

Declaration of Competing Interests

Please complete a declaration of competing interests, considering the following questions:

- Have you in the past five years received reimbursements, fees, funding, or salary from an organisation that may in any way gain or lose financially from the publication of this manuscript, either now or in the future?
- Do you hold any stocks or shares in an organisation that may in any way gain or lose financially from the publication of this manuscript, either now or in the future?
- Do you hold or are you currently applying for any patents relating to the content of the manuscript?
- Have you received reimbursements, fees, funding, or salary from an organization that holds or has applied for patents relating to the content of the manuscript?
- Do you have any other financial competing interests?
- Do you have any non-financial competing interests in relation to this paper?

If you can answer no to all of the above, write 'I declare that I have no competing interests' below. If your reply is yes to any, please give details below.

I declare that I have no competing interests

I agree to the open peer review policy of the journal. I understand that my name will be included on my report to the authors and, if the manuscript is accepted for publication, my named report including any attachments I upload will be posted on the website along with the authors' responses. I agree for my report to be made available under an Open Access Creative Commons CC-BY license (http://creativecommons.org/licenses/by/4.0/). I understand that any comments which I do not wish to be included in my named report can be included as confidential comments to the editors, which will not be published.

I agree to the open peer review policy of the journal

To further support our reviewers, we have joined with Publons, where you can gain additional credit to further highlight your hard work (see: https://publons.com/journal/530/gigascience). On publication of this paper, your review will be automatically added to Publons, you can then choose whether or not to claim your Publons credit. I understand this statement.

Yes Choose an item.
