# Supplementary material for: The genome assembly and annotation of yellowhorn (Xanthoceras sorbifolium Bunge)
Source: Gigascience. 2019 Jun 26;8(6):giz071. doi: 10.1093/gigascience/giz071 (PMC6593362; doi:10.1093/gigascience/giz071)
Supplement: giz071_Reviewer_3_Report_Original_Submission — Pedro Martinez Garcia -- 11/28/2018 Reviewed [file giz071_reviewer_3_report_original_submission.pdf]

Reviewer Report

Title: The genome assembly and annotation of yellowhorn (Xanthoceras sorbifolium Bunge)

Version: Original Submission **Date:** 11/28/2018

Reviewer name: Pedro Martinez Garcia

Reviewer Comments to Author:

This paper reports the genome sequence of yellowhorn, a woody oil-bearing plant of a great economy value in China. The genome assembly presented in this MS after using different sequencing and assembling methodologies, can be a important tool for future studies, as the authors had commented. According to the results seem that the authors have obtained a high quality genome. However, I cannot find the BioProject ID or the BioSample, I think that the authors must clarify it before the MS can be accepted, also some minor comments must be addressed.
Pag6 Line 112, add some references to the formula or a better explanation about the use of the formula.
Page 8 Line 8, I can understand that N50 is a well-known term in genomics but NG50 for the general audience can be unclear, please describe it shortly.
Page 1 Line 141, improve and change the sentence "leading to 6015 gaps were addressed".
Page 11 Line 237, please don't use acronym for APG IV as the first time that it is referenced, change it by the whole name.

Level of Interest

Please indicate how interesting you found the manuscript: Choose an item.

Quality of Written English

Please indicate the quality of language in the manuscript: Choose an item.

Declaration of Competing Interests

Please complete a declaration of competing interests, considering the following questions:

- Have you in the past five years received reimbursements, fees, funding, or salary from an organisation that may in any way gain or lose financially from the publication of this manuscript, either now or in the future?
- Do you hold any stocks or shares in an organisation that may in any way gain or lose financially from the publication of this manuscript, either now or in the future?
- Do you hold or are you currently applying for any patents relating to the content of the manuscript?
- Have you received reimbursements, fees, funding, or salary from an organization that holds or has applied for patents relating to the content of the manuscript?

- Do you have any other financial competing interests?
- Do you have any non-financial competing interests in relation to this paper?

If you can answer no to all of the above, write 'I declare that I have no competing interests' below. If your reply is yes to any, please give details below.

'I declare that I have no competing interests'

I agree to the open peer review policy of the journal. I understand that my name will be included on my report to the authors and, if the manuscript is accepted for publication, my named report including any attachments I upload will be posted on the website along with the authors' responses. I agree for my report to be made available under an Open Access Creative Commons CC-BY license (http://creativecommons.org/licenses/by/4.0/). I understand that any comments which I do not wish to be included in my named report can be included as confidential comments to the editors, which will not be published.

I agree to the open peer review policy of the journal

To further support our reviewers, we have joined with Publons, where you can gain additional credit to further highlight your hard work (see: https://publons.com/journal/530/gigascience). On publication of this paper, your review will be automatically added to Publons, you can then choose whether or not to claim your Publons credit. I understand this statement.

Yes Choose an item.
